# Association of High-Dose Erythropoietin With Circulating Biomarkers and Neurodevelopmental Outcomes Among Neonates With Hypoxic Ischemic Encephalopathy: A Secondary Analysis of the HEAL Randomized Clinical Trial

**DOI:** 10.1001/jamanetworkopen.2023.22131

**Published:** 2023-07-07

**Authors:** Sandra E. Juul, Emily Voldal, Bryan A. Comstock, An N. Massaro, Theo K. Bammler, Dennis E. Mayock, Patrick J. Heagerty, Yvonne W. Wu, Adam L. Numis

**Affiliations:** 1 University of Washington, Seattle; 2 Children’s National Health Systems, Washington, DC; 3 University of California, San Francisco

## Abstract

**Importance:**

The ability to predict neurodevelopmental impairment (NDI) for infants diagnosed with hypoxic ischemic encephalopathy (HIE) is important for parental guidance and clinical treatment as well as for stratification of patients for future neurotherapeutic studies.

**Objectives:**

To examine the effect of erythropoietin on plasma inflammatory mediators in infants with moderate or severe HIE and to develop a panel of circulating biomarkers that improves the projection of 2-year NDI over and above the clinical data available at the time of birth.

**Design, Setting, and Participants:**

This study is a preplanned secondary analysis of prospectively collected data from infants enrolled in the High-Dose Erythropoietin for Asphyxia and Encephalopathy (HEAL) Trial, which tested the efficacy of erythropoietin as an adjunctive neuroprotective therapy to therapeutic hypothermia. The study was conducted at 17 academic sites comprising 23 neonatal intensive care units in the United States between January 25, 2017, and October 9, 2019, with follow-up through October 2022. Overall, 500 infants born at 36 weeks’ gestation or later with moderate or severe HIE were included.

**Intervention:**

Erythropoietin treatment 1000 U/kg/dose on days 1, 2, 3, 4 and 7.

**Main Outcomes and Measures:**

Plasma erythropoietin was measured in 444 infants (89%) within 24 hours after birth. A subset of 180 infants who had plasma samples available at baseline (day 0/1), day 2, and day 4 after birth and either died or had 2-year Bayley Scales of Infant Development III assessments completed were included in the biomarker analysis.

**Results:**

The 180 infants included in this substudy had a mean (SD) gestational age of 39.1 (1.5) weeks, and 83 (46%) were female. Infants who received erythropoietin had increased concentrations of erythropoietin at day 2 and day 4 compared with baseline. Erythropoietin treatment did not alter concentrations of other measured biomarkers (eg, difference in interleukin [IL] 6 between groups on day 4: −1.3 pg/mL; 95% CI, −4.8 to 2.0 pg/mL). After adjusting for multiple comparisons, we identified 6 plasma biomarkers (C5a, interleukin [IL] 6, and neuron-specific enolase at baseline; IL-8, tau, and ubiquitin carboxy-terminal hydrolase-L1 at day 4) that significantly improved estimations of death or NDI at 2 years compared with clinical data alone. However, the improvement was only modest, increasing the AUC from 0.73 (95% CI, 0.70-0.75) to 0.79 (95% CI, 0.77-0.81; *P* = .01), corresponding to a 16% (95% CI, 5%-44%) increase in correct classification of participant risk of death or NDI at 2 years.

**Conclusions and Relevance:**

In this study, erythropoietin treatment did not reduce biomarkers of neuroinflammation or brain injury in infants with HIE. Circulating biomarkers modestly improved estimation of 2-year outcomes.

**Trial Registration:**

ClinicalTrials.gov Identifier: NCT02811263

## Key Points

**Question:**

Can circulating biomarkers improve estimations of neurodevelopmental outcomes at 2 years compared with clinical information alone?

**Findings:**

In this secondary analysis of a randomized clinical trial of 180 neonates with hypoxic ischemic encephalopathy and complete biomarker and outcome data, erythropoietin treatment did not affect the measured circulating biomarker concentrations. A combination of inflammatory mediators and brain-specific proteins drawn at baseline, day 2, and day 4 significantly improved accuracy for estimation of neurodevelopmental outcomes vs that obtained with clinical data alone.

**Meaning:**

These findings suggest that circulating biomarkers may improve projection of 2-year outcomes for newborns with hypoxic ischemic encephalopathy.

## Teaser

This secondary analysis of a randomized clinical trial develops and evaluates a panel of circulating biomarkers to improve the projection of 2-year neurodevelopmental impairment among neonates with hypoxic ischemic encephalopathy.

## Introduction

Neonatal hypoxic-ischemic encephalopathy (HIE) refers to the clinical presentation of a baby that has experienced reduced blood and oxygen flow to the brain near the time of birth. The diagnosis is typically made using a combination of clinical examination and laboratory findings within the first hours after birth.^1^ Therapeutic hypothermia (TH) is the only proven treatment, currently indicated for moderate or severe HIE in high-resource settings.^2,3^ However, despite TH treatment, approximately 50% of infants diagnosed with moderate or severe HIE still experience severe sequelae, including epilepsy, cerebral palsy, cognitive impairment, or death.^4,5,6,7,8^ Since new adjunctive therapies are sought, biomarkers that accurately diagnose the severity of HIE, evaluate response to treatment, and predict short- and long-term neurodevelopmental outcomes are needed.

The High-Dose Erythropoietin for Asphyxia and Encephalopathy (HEAL) Trial was a prospective, randomized, placebo-controlled phase 3 trial in which newborns with moderate or severe HIE were randomized to receive erythropoietin or placebo in conjunction with TH.^9,10^ The primary outcome of death or neurodevelopmental impairment (NDI) showed no beneficial effect of adding erythropoietin to TH. A secondary aim for this trial was to evaluate circulating plasma biomarker concentrations to assess whether (1) erythropoietin decreases inflammation and (2) a combination of biomarkers improves the estimation of neurodevelopmental outcomes at 2 years when compared with using clinical information alone (2 years was defined as 22-36 months in the context of this study). We hypothesized that (1) erythropoietin treatment would result in lower circulating pro-inflammatory cytokines and markers of brain injury compared with placebo; (2) a combination of 1 or more brain specific proteins or cytokines would be associated with death or NDI at 2 years; and (3) adding these biomarkers to clinically available information would improve models for risk of death or NDI.

## Methods

### Study Population

In this preplanned secondary analysis of prospectively collected data from infants enrolled in the HEAL Trial, 500 infants (≥36 weeks’ gestation, with moderate or severe encephalopathy and evidence of perinatal depression, treated with TH) were enrolled and randomized to either TH alone or erythropoietin with TH. Infants were enrolled into the HEAL trial between January 25, 2017, and October 9, 2019, at 17 sites comprising 23 hospitals.^9^ Follow-up was completed October 2022. Parents or legal guardians provided written informed consent for their child to participate in the study. This was a preplanned analysis of the parent trial and follows the Consolidated Standards of Reporting Trials (CONSORT) reporting guideline reporting guideline for randomized studies.^11^ The trial protocol appears in Supplement 1. Erythropoietin concentrations were measured in all infants with plasma at baseline (within 24 hours after birth). A subset of 180 infants was identified for additional biomarker analyses who met the following inclusion criteria: adequate volumes of plasma at baseline, day 2, and day 4; magnetic resonance imaging (MRI) data during the initial hospitalization; and 2-year Bayley Scales of Infant and Toddler Development, Third edition (BSID-III) with standardized neurologic examination and Gross Motor Function Classification System (GMFCS) completed or who had died during the study period. The first 180 infants who met the inclusion criteria were included in this analysis. The trial was approved by the institutional review boards at all participating sites and was registered with the US Food and Drug Administration (Investigational New Drug application 102 138). Maternal race and ethnicity was self-reported and was assessed in this study because such reporting is important to determine whether there were differences in severity of HIE or in outcomes based on race, and whether the results are generalizable.^12^

### Biomarker Determinations

A sample size of 86 neonates per group is required to detect a 0.5-SD difference on the log scale with a single observation per neonate, with 80% power while controlling for a type I error of .0167 (3 markers, Bonferroni). Whole blood specimens (1.5 mL) were collected in heparinized tubes at 3 timed intervals: (1) baseline (median [IQR] 17.0 [12.9-21.8] hours), (2) day 2, drawn 24 ± 2 hours after the first study dose was given, and (3) day 4, 72 ± 2 hours after the first study drug dose. Specimens were centrifuged at 6000 rpm for 10 minutes and plasma collected and stored at −80 °C within 4 hours of collection.

Biomarkers of inflammation included both pro-inflammatory and anti-inflammatory mediators. The MILLIPLEX MAP Human High Sensitivity T Cell Panel from Millipore-Sigma was used to measure 12 analytes: Interleukin (IL) 1b, IL-6, IL-8, IL-10, IL-12p70, IL-13, IL-17A, macrophage inflammatory protein (MIP)–1a/chemokine ligand (CCL) 3, MIP-1b/CCL4, interferon (IFN)-γ, fractalkine/CX3CL1, and tumor necrosis factor (TNF)–α. This high sensitivity panel uses Luminex xMAP technology that allows for multiplex analyte detection with adequate recovery rates in children with neurological disorders.^13^ Putative biomarkers of brain injury in neonatal HIE and other growth factors involved in neuronal development and repair were measured using a Luminex Multiplex assay from R&D Systems and included 14 analytes: brain-derived neurotrophic factor (BDNF), complement component (C5a), intercellular adhesion molecule 1 (ICAM-1)/cluster of differentiation (CD) 54, IL-1 receptor antagonist (IL-1RA), IL-33, monocyte chemoattractant protein 1 (MCP-1)/CCL2, neural cell adhesion molecule (NCAM-1)/CD56, neuroregulin-1-b-1 (NGB), neuron-specific enolase (NSE), S100 calcium-binding protein B (s100b), vascular cell adhesion molecule 1 (VCAM-1)/CD106, ubiquitin carboxy-terminal hydrolase-L1 (UCHL1), and vascular endothelial growth factor (VEGF). Erythropoietin levels were determined with the Human Erythropoietin/EPO Quantikine ELISA Kit from R&D Systems. Glial fibrillary acidic protein (GFAP) was measured using the R-PLEX Human GFAP Assay from Meso Scale Diagnostics. Tau levels were determined with the R-PLEX Human Tau Assay from Meso Scale Diagnostics.

All assays were performed in duplicate, and results were averaged for analyses. All assays were performed according to the manufacturers’ recommended protocols.

### Neurodevelopmental Assessment

NDI was defined as any of the following: cerebral palsy determined by standardized physical examination,^14^ GMFCS score of 1 or greater (ie, unable to walk 10 steps independently),^15^ or cognitive score less than 90 on BSID-III. All neurologic and BSID-III examiners were centrally trained and certified on an annual basis. A GMFCS score was assigned to all participants, regardless of presence of cerebral palsy. Outcome was assessed at 22 to 36 months of age (referred to as 2-year outcome).

### Statistical Analysis

We used descriptive statistics to compare the demographic and baseline maternal and infant characteristics of the infants enrolled in HEAL included in this analysis (180 infants) with the 320 remaining enrolled participants not included in this substudy. Descriptive statistics (medians, IQR, and boxplots) were used to characterize the log2-distribution of each biomarker at baseline, day 2, and day 4. Statistical analyses were performed with the use of R software, version 4.0.2 (R Project for Statistical Computing).

#### Association Between Biomarkers and Treatment

We used an unadjusted rank-sum test to evaluate whether biomarker concentrations differed between infants receiving erythropoietin compared with those receiving placebo. After correcting for multiple comparisons using the Holm method,^16^ only the association between treatment assignment and erythropoietin was statistically significant (see Results section). We therefore used the entire biomarker cohort (n = 180) to examine associations between biomarkers and the 2-year outcome, excluding erythropoietin concentrations from days 2 and 4 samples since they were affected by the treatment assignment.

#### Univariate Associations

To reduce the skewness and influence of outliers, we log2-transformed and 95% winsorized the biomarker concentrations. Due to the very small amount of missing data in each biomarker, we used a complete case analysis. The primary outcome was a composite outcome of death or NDI at 2 years of age. We tested associations between each biomarker from each of the 3 time points with the primary outcome using a generalized linear model with log link regression with robust standard errors and adjusted for baseline HIE severity, treatment assignment, and site. Associations were quantified using relative risk (RR) and 95% CIs; the RR represents the change in risk of death or NDI associated with a doubling in the biomarker concentration. We defined statistical significance as *P* < .05 after adjusting for multiple comparisons using the Holm-Bonferroni method.^16^

#### Multivariable Associations Within Time Points

We constructed 3 multivariable models (one each for baseline, day 2, and day 4) using least absolute shrinkage and selection operator (LASSO).^17^ All LASSO models were adjusted for HIE severity and treatment assignment; site was also included and allowed to be penalized. We used multiple imputation with chained equations to account for missing data and used bootstrap sampling at the participant level to address model stability.^18^ Biomarkers were included in the multivariable model if they were selected by LASSO in at least half of the bootstrapped samples. The final models were fit and pooled over the imputed data sets, adjusted for HIE severity, treatment assignment, and site.

Twenty-eight biomarkers were initially included in our assessment. To develop a sparser model, we used postselection inference to fit reduced models for each time point that included only the 5 that were selected most often by LASSO in each model.^19^

#### Multivariable Associations Across Time Points

We next examined the biomarkers selected by LASSO within each time point, with the goal of assembling a model that leverages information across time points while minimizing the number of biomarkers involved. For the sake of parsimony, we included only the 3 most selected biomarkers from the baseline and day 4 models (we excluded day 2 biomarker concentrations from the final model as they generally performed less well than the baseline and day 4 concentrations). Because many biomarkers were highly correlated across time points, we did not include repeated measures of any biomarker. The model was adjusted for HIE severity, treatment assignment, and site.

To determine whether adding the information from circulating biomarkers improved our ability to project outcomes compared with data available clinically at the time of study entry, we fit our adjusted model with the following clinical measurements: 5-minute Apgar score, lowest blood pH, highest base deficit, whether there was resuscitation after 10 minutes, and sex. To evaluate how these models performed, we calculated area under the receiver operating characteristic (ROC) curve (AUC) using repeated 10-fold cross-validation. We compared paired ROC curves using the DeLong method using the algorithm of Sun and Xu.^20,21^

## Results

**Figure 1.**
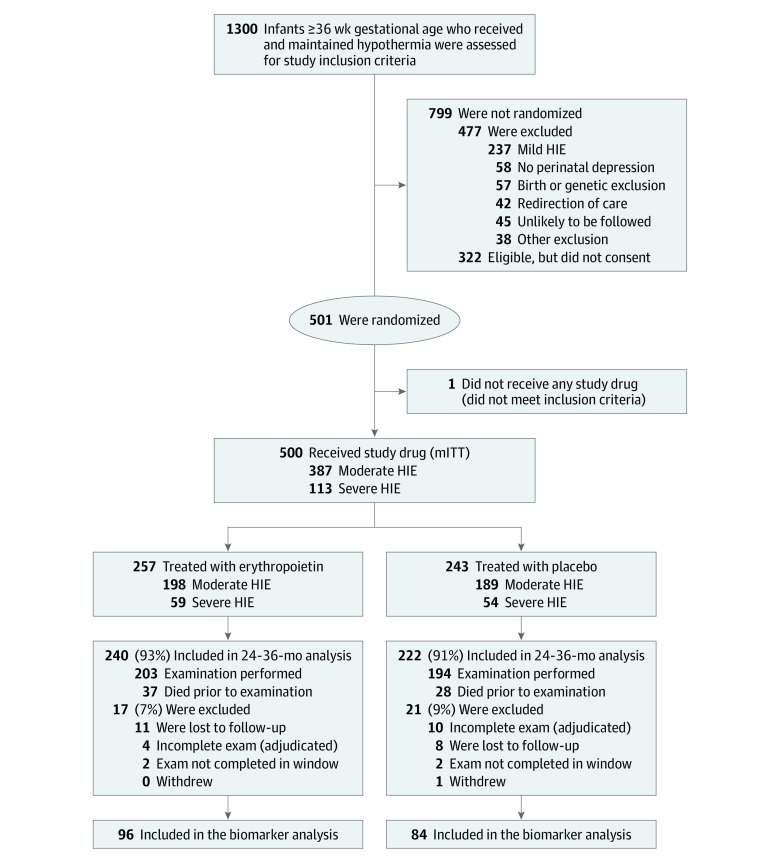
Study Flow Diagram HIE indicates hypoxic ischemic encephalopathy; mITT, modified intention to treat.

Among 180 neonates included in this preplanned analysis of the HEAL trial, there were 83 females (46%) and the mean (SD) gestational age at birth was 39.1 (1.5) weeks. The remaining baseline characteristics of mothers and infants and any complications related to pregnancy and delivery are shown in Table 1. There were no differences between participants included in this analysis and the remaining HEAL participants who did not meet study inclusion criteria (n = 320), obviating the need to adjust for baseline characteristics due to an unbalancing of potential confounders compared with initial randomization. Enrollment into the HEAL Trial and the subset included in this analysis are shown in the study flow diagram (Figure 1).

**Table 1.** Maternal, Pregnancy, and Infant Characteristics at Baseline

Characteristic	Participants, No. (%)		
	Overall (N = 500)	Not included in biomarker analysis (n = 320)	Included in biomarker analysis (n = 180)
Maternal characteristics			
Race and ethnicity^a^			
African American or Black	66 (13)	43 (13)	23 (13)
Asian	33 (7)	25 (8)	8 (4)
Hispanic or Latinx	122 (24)	73 (23)	49 (27)
White	356 (71)	226 (71)	130 (72)
Multiple, other, or unknown^b^	45 (9)	26 (8)	19 (11)
Age, mean (SD), y	29.64 (6.35)	29.32 (6.06)	30.23 (6.80)
Education, high school or less	185 (37)	112 (35)	73 (41)
Parity of 1, including trial infant	286 (57)	183 (57)	103 (57)
Pregnancy and delivery complications			
Maternal chorioamnionitis or fever	65 (13)	37 (12)	28 (16)
Preeclampsia or eclampsia	45 (9)	32 (10)	13 (7)
Gestational diabetes	58 (12)	34 (11)	24 (13)
Obesity: body mass index >30^c^	89 (18)	58 (18)	31 (17)
Sentinel event			
Any^d^	143 (29)	85 (27)	58 (32)
Shoulder dystocia	32 (6)	19 (6)	13 (7)
Placental abruption	71 (14)	47 (15)	24 (13)
Prolapsed cord	23 (5)	11 (3)	12 (7)
Uterine rupture	24 (5)	13 (4)	11 (6)
Cesarean delivery	329 (66)	205 (64)	124 (69)
Outborn delivery	415 (83)	264 (82)	151 (84)
Infant characteristics			
Sex			
Female	225 (45)	142 (44)	83 (46)
Male	275 (55)	178 (56)	97 (54)
Birth weight, mean (SD), g	3372 (594)	3380 (569)	3359 (636)
Gestational age, mean (SD), wk	39.1 (1.5)	39.1 (1.5)	39.1 (1.5)
5-min Apgar, median (IQR)	3.00 (2.00-5.00)	3.00 (2.00-5.00)	3.00 (2.00-5.00)
10-min Apgar, median (IQR)	5.00 (3.00-7.00)	5.00 (3.00-7.00)	5.00 (3.00-6.25)
Continued resuscitation at 10 min^e^	460 (92)	297 (93)	163 (91)
Lowest pH, mean (SD)^f^	6.93 (0.17)	6.92 (0.17)	6.94 (0.18)
Worst base deficit, mean (SD)^f^	−18.32 (6.17)	−18.63 (6.15)	−17.78 (6.18)
Severe encephalopathy^g^	113 (23)	65 (20)	48 (27)
Erythropoietin treatment	257 (51)	161 (50)	96 (53)

^a^ Race and ethnic group were reported by the mother or father.

^b^ Other race includes Native Hawaiian or other Pacific Islander and American Indian or Alaska Native.

^c^ Body mass index is calculated as weight in kilograms divided by height in meters squared.

^d^ A sentinel event was defined as shoulder dystocia, placental abruption, prolapsed cord, or uterine rupture.

^e^ Ongoing resuscitation with chest compressions, mechanical ventilation, or both was warranted at 10 minutes of age.

^f^ Shown is the lowest pH or worst base deficit among cord arterial, cord venous, and arterial blood gas samples obtained before 60 minutes of age.

^g^ Severe encephalopathy was defined according to Sarnat criteria (eTable 1 in Supplement 2).

Table 2 shows the primary 2-year NDI outcome stratified by severity of HIE (as determined by the modified Sarnat examination done at study entry). It is notable that clinical categorization of HIE severity was associated with later NDI: of the 132 infants with moderate HIE, 94 (72%) were either normal or mildly impaired at 2 years of age compared with only 16 of the 48 infants (33%) with severe HIE (*P* < .001). Similarly, HIE clinical classification was associated with death, with 44% mortality in the severe group (21 of 48) compared with 2% mortality in the moderate HIE group (3 of 132; *P* < .001). Adjusted for treatment assignment and site, the RR for death associated with HIE severity was 1.89 (95% CI, 1.44-2.46; *P* < .001).

**Table 2.** Outcomes for Biomarker Cohort, Both Overall and Subset by Baseline HIE Severity

Outcome	Infants, No. (%)		
	Overall (n = 180)	Moderate HIE (n = 132)	Severe HIE (n = 48)
Primary outcome			
No death or NDI	91 (51)	80 (61)	11 (23)
Death or NDI	89 (49)	52 (39)	37 (77)
Ordinal outcome			
Normal	91 (51)	80 (61)	11 (23)
Mild NDI	19 (11)	14 (11)	5 (10)
Moderate to severe NDI	46 (26)	35 (27)	11 (23)
Died	24 (13)	3 (2)	21 (44)

Abbreviations: HIE, hypoxic ischemic encephalopathy; NDI, neurodevelopmental impairment.

We next evaluated whether erythropoietin treatment affected biomarker concentrations. As expected, erythropoietin concentrations were increased from baseline at days 2 and 4 among infants who received erythropoietin treatment. Other biomarkers that nominally appeared to differ between treatment groups included: BDNF in the baseline sample and tau, fractalkine, and IL-17A in the day 4 sample. However, when adjusted for multiple comparisons, no significant differences remained (eg, difference in interleukin [IL] 6 between groups on day 4: −1.3 pg/mL; 95% CI, −4.8 to 2.0 pg/mL) (eTable 5 in Supplement 1). We therefore proceeded to use the entire biomarker cohort (excluding erythropoietin concentrations at days 2 and 4) to examine associations between biomarkers and the primary outcome.

**Figure 2.**
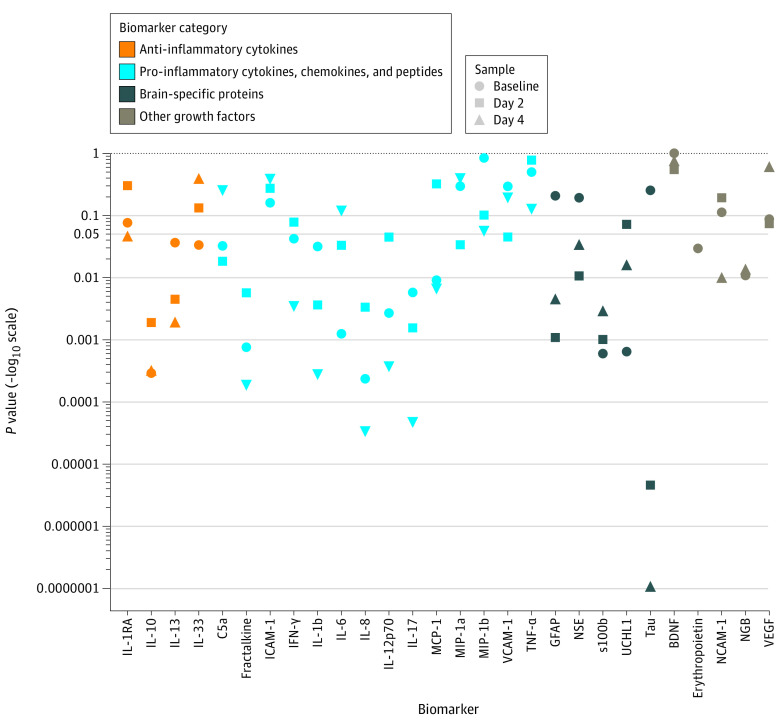
Association Between Biomarkers and Death or Neurodevelopmental Impairment Y-axis shows unadjusted *P* values from univariate analyses, with individual biomarkers along the x-axis. Relative risks and confidence intervals for these *P* values are in eTable 1 in Supplement 2. Biomarkers across all categories (anti-inflammatory, pro-inflammatory, brain-specific, and growth categories) at each time point were associated with death or neurodevelopmental impairment at 2 years. *P* values are not indicative of effect size. BDNF indicates brain-derived neurotrophic factor; GFAP, glial fibrillary acidic protein; ICAM-1, intercellular adhesion molecule 1; IFN-γ, interferon-γ; IL, interleukin; MCP, monocyte chemoattractant protein; MIP, macrophage inflammatory protein; NCAM-1, neural cell adhesion molecule; NGB, neuroregulin-1-b-1; NSE, neuron-specific enolase; s100b, S100 calcium-binding protein B; TNF-α, tumor necrosis factor–α; UCHL1, ubiquitin carboxy-terminal hydrolase-L1; VCAM, vascular cell adhesion molecule; VEGF, vascular endothelial growth factor.

Figure 2 shows a Manhattan plot of the unadjusted *P* values for the univariate associations of biomarker concentrations with death or NDI at 2 years. Baseline erythropoietin was measured in 444 infants. The median (IQR) erythropoietin concentration at baseline in those infants with no death or NDI was 26.6 (13.4-53.0) mU/mL compared with 44.0 (14.4-287.3) mU/mL in those with death or NDI (RR 1.05 (95% CI, 1.00-1.10). After adjusting for multiple comparisons, 4 of 28 biomarker concentrations (14%; IL-8, IL-10, s100b, and UCHL1; eg, IL-10: RR, 1.13; 95% CI, 1.06-1.21; *P* < .001) at baseline were associated with death or NDI, 1 of 27 (4%; tau: RR, 1.32; 95% CI, 1.17-1.49; *P* < .001) at day 2, and 7 of 27 (26%; fractalkine, IL-b, IL-8, IL-10, IL-12p70, IL-17A, tau; eg, IL-17A: RR, 1.25; 95% CI, 1.12-1.40; *P* < .001) at day 4 (eTable 1 in Supplement 2).

**Figure 3.**
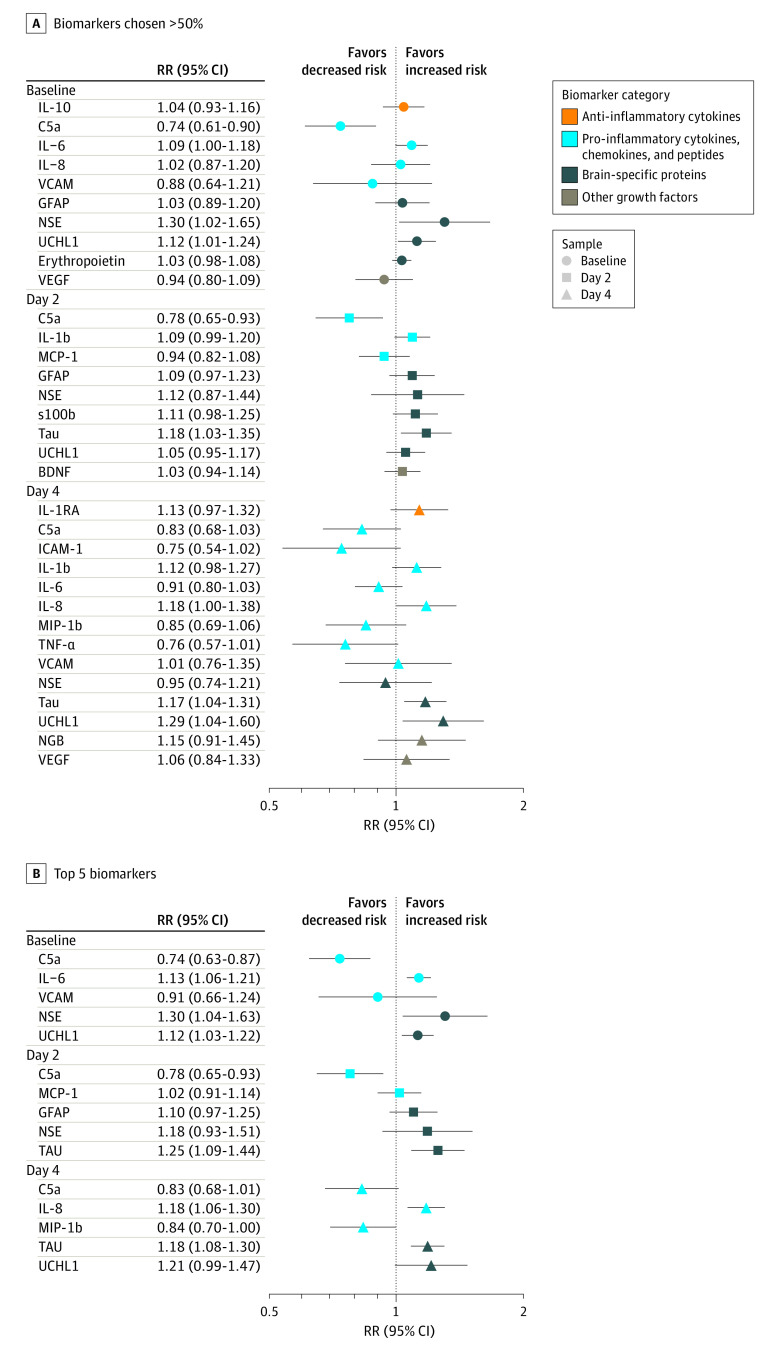
Forest Plot of Relative Risks (RRs) From Least Absolute Shrinkage and Selection Operator–Selected Multivariable Models A, All biomarkers selected at least 50% of the bootstrapped least absolute shrinkage and selection operator models. B, The 5 most selected biomarkers by bootstrapped least absolute shrinkage and selection operator samples. A set of 9 biomarkers at baseline, day 2, and day 4 remained significant in multivariable analyses after adjustment for hypoxic ischemic encephalopathy severity, treatment assignment, and site. BDNF indicates brain-derived neurotrophic factor; GFAP, glial fibrillary acidic protein; ICAM-1, intercellular adhesion molecule 1; IL, interleukin; MCP, monocyte chemoattractant protein; MIP, macrophage inflammatory protein; NGB, neuroregulin-1-b-1; NSE, neuron-specific enolase; s100b, S100 calcium-binding protein B; TNF-α, tumor necrosis factor–α; UCHL1, ubiquitin carboxy-terminal hydrolase-L1; VCAM, vascular cell adhesion molecule; VEGF, vascular endothelial growth factor.

We next evaluated biomarker concentrations and their association with death or NDI using a multivariable model with LASSO. Figure 3A is a forest plot of the RRs and 95% CIs for biomarkers that were chosen in 50% or more bootstrapped LASSO samples at each time point, and Figure 3B is a reduced model with only the 5 most-selected biomarkers at each time point.^19^ Of the biomarkers identified by LASSO, changes in concentrations of C5a (RR, 0.74; 95% CI, 0.61-0.90), IL-6 (RR, 1.09; 95% CI, 1.00-1.18), NSE (RR, 1.30; 95% CI, 1.02-1.65), and UCHL1 (RR, 1.12; 95% CI, 1.01-1.24) at baseline; C5a (RR, 0.78; 95% CI, 0.65-0.93) and tau (RR, 1.18; 95% CI, 1.03-1.35) at day 2; and IL-8 (RR, 1.18; 95% CI, 1.00-1.38), tau (RR, 1.17; 95% CI, 1.04-1.31), and UCHL1 (RR, 1.29; 95% CI, 1.04-1.60) at day 4 were independently associated with death or NDI at 2 years. The percentage of LASSO samples including each biomarker is shown in eFigure 1 in Supplement 2.

To leverage biomarker data across time points, we selected a curated set of biomarkers from the LASSO-based multivariable models that included the pro-inflammatory proteins C5a and IL-6 at baseline, the anti-inflammatory protein IL-8 at day 4, and brain-specific proteins NSE (baseline), tau (day 4), and UCHL1 (day 4). We developed a cumulative model that included our biomarker data sets (baseline, day 2, day 4, and curated set) as well as pertinent clinical data known at study enrollment.^22^ We then evaluated whether the biomarker data sets increased our ability to estimate 2-year NDI or death compared with clinically available data alone (sex, 5-minute Apgar score, resuscitation at 10 minutes, worse base deficit, lowest pH, and severity of HIE based on Sarnat examination), shown in eTable 2 in Supplement 2. eFigure 2 in Supplement 2 shows the comparison of ROC curves derived from each model. Compared with a model with clinical data alone (AUC, 0.73; 95% CI, 0.70-0.75), the addition of biomarker data from the baseline time point (AUC, 0.77; 95% CI, 0.75-0.80; *P* = .04), day 4 (AUC, 0.79; 95% CI, 0.77-0.81; *P* = .003), or the curated set across times points (AUC, 0.78; 95% CI, 0.75-0.80; *P* = .03) increased model performance. In eTable 3 in Supplement 2, we show the AUC from the various models, and eTable 4 in Supplement 2 includes adjusted *P* values for comparisons of AUC between models.

## Discussion

In this ancillary study of a contemporary multicenter randomized, placebo-controlled trial in newborns born at full term with HIE receiving TH, we found that, contrary to our first hypothesis, treatment with erythropoietin was not associated with changes in 18 inflammatory mediators at baseline, day 2, or day 4. This finding was surprising given that erythropoietin-associated neuroprotection demonstrated in preclinical studies has been partly attributed to anti-inflammatory mechanisms in animal models^23,24^ and may in part account for the lack of neuroprotective effect of erythropoietin treatment observed in the HEAL Trial. We also did not find evidence that erythropoietin treatment reduced other brain injury biomarker levels, consistent with the lack of benefit from erythropoietin treatment in the HEAL Trial^9^ and also consistent with our prior reports that evaluated the effect of erythropoietin on circulating biomarkers in the Phase 2 NEATO Study (NCT01913340)^25^ and in the Preterm Epo Neuroprotection Trial (NCT01378273).^26^

Our second hypothesis, that a combination of 1 or more brain-specific proteins or cytokines would be associated with the presence and severity of brain injury, was confirmed. We demonstrated an association of several circulating biomarkers with death or NDI at 2 years of age across all biomarker categories (pro-inflammatory, anti-inflammatory, brain-specific proteins, and other growth factors) and at all time points. We then selected putative plasma biomarkers of brain injury from an array of candidate inflammatory cytokines, brain-specific proteins, and growth factors into a final curated model. After adjusting for multiple comparisons, we identified 6 plasma biomarkers (C5a, IL-6, and NSE at baseline; IL-8, tau, and UCHL1 at day 4) that significantly improved estimation of death or NDI at 2 years compared with clinical data alone. However, the improvement was only modest, increasing the AUC from 0.73 (95% CI, 0.70-0.75) to 0.79 (95% CI, 0.77-0.81, *P* = .01), corresponding to a 16% (95% CI, 5%-44%) increase in our ability to correctly classify participants’ risk of death or NDI at 2 years.

Our findings confirm prior, largely single-center, observational studies suggesting that circulating inflammatory cytokines may provide insights into the propagation of secondary brain injury after hypoxia-ischemia leading to NDI.^25,27,28,29,30,31^ Given the complexity of the inflammatory response in both number of potential mediators, positive and negative feedback associations, and variable time evolution (eg, some with biphasic response),^32^ results of prior studies have been inconsistent in identifying which cytokines are predictive of adverse outcomes and when they should be measured. We leveraged this study’s large data set and used a data-driven approach to refine our selection of key inflammatory biomarkers among a robust list of potential mediators measured over time. We developed models individually for each timed sample and then assessed a cumulative model leveraging data across all 3 time points. This pragmatic approach allowed us to provide model outputs evaluating data at each individual time point, as well as an overall model incorporating all available data.

Our curated model included 3 pro-inflammatory proteins: IL-6, IL-8, and C5a. Our findings support early measurement (ie, in the first 24 hours of life) of pro-inflammatory cytokine IL-6, consistent with prior studies that have evaluated comparative predictive abilities of serial IL-6 measurements.^27,31^ We also observed that elevated IL-8 measured on day 4 was associated with additive risk for adverse outcome, consistent with ongoing inflammation even after rewarming. Of interest, higher concentrations of C5a at baseline were associated with lower risk for NDI. This was somewhat surprising given that activation of the complement cascade with release of C5a as a pro-inflammatory effector molecule is known to occur after perinatal asphyxia.^33^ Noncanonical effects of complement activation in the brain may provide a mechanism for neuroprotection in the developing brain through its effect on proliferation of neural progenitor cells.^34,35,36^ Prolonged complement inhibition may interfere with its reparative role and may be deleterious beyond the acute postinjury phase,^37^ given that C5a has been demonstrated to play an important role in astrogliosis and repair after neural injury.^38^

Our curated set of biomarkers also included the brain specific proteins UCHL1,^25,28,39^ NSE,^40,41^ and tau,^25,30,31^ all of which have been reported as promising biomarkers of brain injury in prior neonatal HIE studies. UCHL1 (also known as neuronal-specific gene product 9.5)^42^ and NSE^43^ are proteins concentrated in neuronal cytoplasm, while tau is a microtubule-associated protein found in the axons of neurons.^44^ It is important to note that while other previously reported candidate biomarkers GFAP^30^ and s100B^41^ demonstrated associations with 2-year outcomes in unadjusted analyses, LASSO selected UCHL1, NSE, and tau most consistently for a combinative multivariable model. The association of tau protein levels with neurodevelopmental outcomes is consistent with recent studies suggesting tau as a leading brain-injury biomarker in HIE.^25,31^

To address our third hypothesis, that adding selected biomarkers to clinically available information would improve projection of 2-year neurodevelopmental outcomes, we assessed the added value of biomarker determinations in combination with baseline and clinical characteristics. While we chose factors that have been previously reported as indicators of HIE severity and risk for adverse outcomes,^22^ we acknowledge that our models may not include all relevant clinical covariates. We focused on clinical factors readily assessable at or shortly after birth and at the bedside, as our goal was to assess the added value of biomarker determinations in this setting. We did not aim to assess whether biomarkers improved projections over electroencephalogram and MRI findings, which have been associated with risk for adverse neurodevelopmental outcomes in this population.^45,46,47,48^ Our curated set of biomarkers enhanced projection of death or NDI over clinically available data alone. Early projection (ie, baseline) is critical for early risk stratification and identification of patients appropriate for neurotherapeutic interventions, while incorporation of cumulative data over time may provide more accurate estimation for prognostication and direction of later rehabilitative therapies. We have recently published that MRI or magnetic resonance spectroscopy done on days 4 to 6 after rewarming was poorly predictive of degree of NDI in the absence of severe injury.^49^ Whether biomarkers combined with neurophysiological and neuroimaging studies can improve overall prediction of adverse outcomes is an area for future study. Given the high AUC observed using just clinically available data, these data can be used in lower-resource settings where neuromonitoring and laboratory capabilities are limited or unavailable. Combining our biomarker data with clinically available data increases the accuracy incrementally and may be useful for clinical trials.

To our knowledge, this is the largest multicenter study evaluating biomarker data from a contemporary cohort of cooled neonates with HIE. Strengths of our study include biomarker and neurodevelopmental outcome assessments performed within the rigor of a multicenter randomized clinical trial. Our data-driven analytical approach and adjustments for multiple comparisons allowed us to screen a robust array of candidate biomarkers and select the most promising biomarkers to develop a cumulative model.

### Limitations

This study has limitations. While blood sample collection was performed according to the HEAL Trial manual of operations and we adjusted for site variability in our analyses, we cannot exclude the possibility that variability within sites with regards to handling and processing of samples may have affected results. The strength of associations between biomarkers and outcomes may also differ if other clinical covariates and neurodiagnostic assessment tools were considered in our analyses. Furthermore, only 180 of 500 potential samples were evaluated as guided by our power calculations and cost, and this may have increased the risk of bias. Future prospective validation of our models is warranted prior to incorporation of biomarker measurements into clinical care for neonates with HIE.

## Conclusions

In this study, erythropoietin treatment did not attenuate biomarkers of neuroinflammation or brain injury in neonates with HIE. In the sample of 180 infants, biomarkers measured at baseline (C5a, IL-6, and NSE) and day 4 (IL-8, tau, and UCHL1) incrementally improved estimation of 2-year outcomes when added to clinical characteristics in this high-risk population.

## Notes

Supplement 1.
Trial Protocol

Supplement 2. **eTable 1.** Univariate Relative Risks (RRs) for the Association Between Each Biomarker Measure and the Primary Outcome of Death or Neurodevelopmental Impairment **eFigure 1.** Selection of Biomarkers by LASSO Within Each Time Point **eFigure 2.** ROC Curves **eTable 2.** Predictive Ability of Biomarker Data Sets **eTable 3.** AUC From Multivariable Models of Biomarker Concentrations and Their Association With Death or NDI **eTable 4.** Adjusted *P* Values for Comparisons of AUC Between Multivariable Models of Biomarker Concentrations and Their Association With Death or NDI **eTable 5.** Median Biomarker Values by Treatment Group and Rank-Sum Differences Between Groups

Supplement 3.
Nonauthor Collaborators

Supplement 4.
Data Sharing Statement
