# Supplementary material for: Association of High-Dose Erythropoietin With Circulating Biomarkers and Neurodevelopmental Outcomes Among Neonates With Hypoxic Ischemic Encephalopathy: A Secondary Analysis of the HEAL Randomized Clinical Trial
Source: JAMA Netw Open. 2023 Jul 7;6(7):e2322131. doi: 10.1001/jamanetworkopen.2023.22131 (PMC10329214; doi:10.1001/jamanetworkopen.2023.22131)
Supplement: Supplement 1. — Trial Protocol [file jamanetwopen-e2322131-s001.pdf]

HIGH DOSE ERYTHROPOIETIN FOR ASPHYXIA AND ENCEPHALOPATHY

A randomized, placebo-controlled, double-masked 500 - subject clinical trial

of erythropoietin for the treatment of

neonatal hypoxic-ischemic encephalopathy (HIE)

Study Chairs:

Yvonne Wu, MD, MPH (Multi-PI CCC)
Professor of Neurology and Pediatrics
Division of Child Neurology
University of California, San Francisco

Sandra Juul, MD, PhD (Multi-PI CCC)
Professor of Pediatrics
Division of Neonatology
University of Washington, Seattle

Patrick Heagerty, PhD (PI of DCC)
Professor and Chair of Biostatistics
University of Washington, Seattle

Funded by:

The National Institute of Neurological Disorders and Stroke (NINDS)
1U01NS092764-01
5U01NS092553-02

Sponsor of active IND:

Yvonne Wu, MD, MPH
IND 102,138

KEY STUDY PERSONNEL

**<u>Study Chairs</u>**	
Yvonne Wu, MD, MPH (Multi-PI CCC)<br>Professor of Neurology and Pediatrics<br>Division of Child Neurology<br>University of California, San Francisco	Sandra Juul, MD, PhD (Multi-PI CCC)<br>Professor and Chief<br>Division of Neonatology<br>University of Washington, Seattle
Patrick Heagerty, PhD (PI of DCC)<br>Professor and Chair of Biostatistics<br>University of Washington, Seattle	
**<u>HUB Leaders</u>**	
Fernando Gonzalez, MD<br>University of California San Francisco<br>San Francisco, CA	Dennis Mayock, MD<br>University of Washington<br>Seattle, WA
**<u>Neuroimaging Core</u>**<br>Jessica Wisnowski, PhD<br>University of Southern California<br>Los Angeles, CA	Amit Mathur, MD<br>St. Louis University<br>St. Louis, MO
Robert McKinstry, MD, PhD<br>Washington University<br>St Louis, MO	Stefan Bluml, PhD<br>University of Southern California<br>Los Angeles, CA
Ashok Panigrahy, MD<br>University of Pittsburgh Medical Center<br>Pittsburgh, PA	Yvonne Wu, MD MPH<br>University of California San Francisco<br>San Francisco, CA
**<u>Follow-Up Core</u>**	
Karl Kuban, MD<br>Boston Medical Center/Boston University<br>Boston, MA	Jean Lowe, PhD<br>University of New Mexico<br>Albuquerque, New Mexico
Elizabeth Rogers, MD<br>University of California, San Francisco<br>San Francisco, CA	Michael O'Shea, MD, MPH<br>University of North Carolina<br>Chapel Hill, NC
**<u>Biomarker Core</u>**	
Sandra Juul, MD, PhD<br>University of Washington<br>Seattle, WA	An Massaro, MD<br>Children's National Medical Center<br>Washington, DC
**<u>Medical Monitor</u>**<br>Michael Schreiber, MD<br>University of Chicago Medicine<br>Chicago, IL	**<u>NINDS Representatives</u>**<br>Adam Hartman, MD, and<br>Scott Janis, MD<br>NIH NINDS, Bethesda, MD

SUMMARY

Study Name	HEAL Trial: <u>H</u>igh-dose <u>E</u>rythropoietin for <u>A</u>sphyxia and Encepha<u>l</u>opathy
Study Drug	Erythropoietin (Epo), Epoetin alfa, 4000 Units/mL
Subject Population	500 infants with moderate to severe HIE undergoing therapeutic hypothermia
Objectives	1) To determine if 5 doses of Epo 1000 U/kg (birth weight) intravenous (IV) reduces the rate of death or neurodevelopmental impairment (mild, moderate, or severe) at 24 months of age.<br>2) To assess safety of Epo.<br>3) To determine whether Epo decreases the severity of HIE-induced brain injury as evidenced by early MRI and plasma biomarkers of brain injury.
Design	Prospective, randomized, double-masked, placebo-controlled study
Arms	Treatment: Hypothermia (standard of care) plus Epo<br>Placebo: Hypothermia (standard of care) plus normal saline
Procedures	- Screen all cooled infants to evaluate eligibility for HEAL<br>- Determine severity of encephalopathy using modified Sarnat exam<br>- Consent and randomize patients<br>- Randomized subjects receive five doses of either study drug or placebo<br>- First dose administered within 24+2 hours of age (Study Day 1); subsequent doses given at the same time of day on Study Days 2, 3, 4, and 7<br>- Brain MRI and MR spectroscopy ideally performed between 96 and 144 hours post-birth, as part of routine clinical care<br>- Blood collected prior to administration of first study drug dose, and on Study Days 2 and 4 (3 samples total, 1.5 mL each)<br>- Urine (2 samples) collected on Study Days 0-1, and after completion of rewarming on Study Days 3-4<br>- Phone follow-up at 4, 8, 12, 18, and 24 months: parental questionnaires regarding intervening medical and developmental history, and developmental milestones (Warner Initial Developmental Evaluation of Adaptive and Functional Skills (WIDEA), 12, 18, and 24 months only); All phone interviews may be done in person if desired<br>- 24-month in-clinic evaluation includes a standardized neurologic exam, Bayley III, Gross Motor Function Classification System (GMFCS) evaluation, Child Behavior Checklist (CBCL), and growth parameters<br>- Extended contact phone calls every 6 months from 2.5 through 8 years of age
Sites	At least 15, and up to 30 centers in the United States

TABLE OF CONTENTS

SYNOPSIS **8**

1 STUDY OBJECTIVES **9**

1.1 SPECIFIC AIMS 9

1.1.1 Aim 1: Efficacy *9*

1.1.2 Aim 2: Safety *9*

1.1.3 AIM 3: Biomarkers of Neonatal Brain Injury *10*

1.2 ENDPOINTS 10

1.3 OVERVIEW OF STUDY DESIGN 10

1.3.1 Definition of Study Day *10*

2 BACKGROUND **13**

2.1 RATIONALE 13

2.1.1 Patient population *13*

2.1.2 Mechanisms of Epo Neuroprotection *13*

2.1.3 Pre-clinical studies of Epo in neonatal HI brain injury *14*

2.1.4 Dosing regimen *14*

2.1.4.1 Why multiple and late doses? 15

2.1.4.2 Dose selection 15

2.1.4.3 Route of administration 16

2.1.5 Need, relevance and priority for the study *16*

2.2 SUPPORTING DATA 16

2.2.1 Clinical studies of Epo and HIE *16*

2.2.2 Safety *17*

2.2.3 Pilot studies *18*

3 STUDY DESIGN **21**

4 SELECTION AND ENROLLMENT OF SUBJECTS **21**

4.1 INCLUSION CRITERIA 21

4.1.1 Prior therapy *22*

4.1.2 Demographic characteristics *22*

4.2 EXCLUSION CRITERIA 22

4.3 STUDY ENROLLMENT PROCEDURES 22

4.3.1 Identification and recruitment *22*

4.3.2 Screening for eligibility and monitoring of recruitment targets *23*

4.3.3 Consent procedures *23*

4.3.4 Stratification, Randomization and Masking Procedures 23

5 STUDY INTERVENTIONS 23

5.1 STUDY DRUG ADMINSTRATION AND DURATION 23
5.2 HANDLING OF STUDY DRUG 24
5.3 CONCOMITANT AND REQUIRED INTERVENTIONS 25
5.4 PROHIBITED AND PRECAUTIONARY INTERVENTIONS 25
5.5 ADHERENCE ASSESSMENT 25

6 CLINICAL AND LABORATORY EVALUATIONS 26

6.1 SCHEDULE OF EVALUATIONS 26
6.2 TIMING OF EVALUATIONS 29
6.2.1 Pre-Randomization Evaluations – Timing 29
6.2.2 Birth Hospitalization Evaluations – Timing 29
6.2.3 Follow-Up Evaluations – Timing 29
6.2.4 Final Endpoint Evaluation 30
6.2.5 Future Studies 30
6.3 INSTRUCTIONS AND DEFINITIONS OF EVALUATIONS 30
6.3.1 Informed Consent 30
6.3.2 Clinical assessment 30
6.3.2.1 Screening clinical assessment (<24+2 hours): 30
6.3.2.2 Demographics 32
6.3.2.3 Day five clinical assessment 32
6.3.2.4 Newborn hearing screening 32
6.3.3 EEG assessments 33
6.3.3.1 EEG interpretation 33
6.3.4 Laboratory Evaluations 33
6.3.5 Plasma and urine biomarkers; baseline Epo measurement 33
6.3.6 Neuroimaging: Brain MRI/MRS 34
6.3.7 Phone questionnaires 34
6.3.8 Neurodevelopmental examinations (24 months) 34
6.3.8.1 Standardized neurologic examination 35
6.3.8.2 Gross Motor Function Classification Scale (GMFCS)[131] 36
6.3.8.3 Bayley Scales of Infant Development III (Bayley-III) 37
6.3.8.4 Developmental history 37
6.3.8.5 Medical and social history 37

6.3.8.6 Growth parameters ... 37
6.3.8.7 Child Behavior Checklist (CBCL) ... 37
6.3.8.8 Warner Initial Developmental Evaluation of Adaptive and Functional Skills (WIDEA) 38
6.3.9 Extended Contact Phone Calls ... 38
7 MANAGEMENT OF SERIOUS ADVERSE EVENTS AND COMPLICATIONS OF HIE ... 38
7.1 SERIOUS ADVERSE EVENT DEFINITIONS ... 39
7.2 SAE RELATEDNESS & SEVERITY ... 40
7.3 SAE AND UNEXPECTED, RELATED EVENT REPORTING ... 40
7.4 ANTICIPATED MEDICAL COMPLICATIONS (ADVERSE EVENTS) IN HIE PATIENTS ... 43
7.5 CRITERIA FOR MODIFICATION OF INTERVENTION REGIMEN ... 43
7.5.1 Criteria for Withholding/Stopping the Study Drug ... 43
7.5.2 Restarting Study Drug ... 43
7.6 PROTOCOL VIOLATION/ DEVIATION REPORTING ... 43
7.6.1 Protocol Violations: ... 43
7.6.2 Protocol Deviations may include the following items, among others: ... 44
8 CRITERIA OF INTERVENTION DISCONTINUATION ... 44
9 STATISTICAL CONSIDERATIONS ... 44
9.1 GENERAL ... 44
9.2 OUTCOMES ... 45
9.2.1 Primary outcome ... 45
9.2.1.1 Motor Deficit – Cerebral Palsy ... 45
9.2.1.2 Motor Deficit: Gross Motor Functional Classification System (GMFCS) ... 46
9.2.1.3 Cognitive Deficit: Bayley III Cognitive Score < 90 ... 46
9.2.1.4 Adjudication of Primary Outcome ... 46
9.2.2 Secondary outcomes ... 47
9.3 DATA ANALYSES ... 47
9.3.1 Primary outcome analyses ... 47
9.3.2 Secondary Outcomes ... 48
9.3.3 Exploratory Analyses ... 48
9.3.4 Analyses of Safety Data ... 48
9.3.5 Neuroimaging and circulating biomarker analyses ... 49
9.3.6 Compliance, Retention, and Missing Data ... 49
9.3.6.1 Missing data and dropouts. ... 49
9.4 SAMPLE SIZE AND ACCRUAL ... 50
9.4.1 Primary Outcome Sample Size Calculations: ... 50

9.4.2 Sample size for efficacy secondary outcomes ... 52
9.4.3 Sample size for MRI/MRS biomarkers ... 53
9.4.4 Sample size for plasma biomarkers of brain injury and inflammation ... 53
9.4.5 Achieving the sample size. ... 54
9.5 DATA MONITORING ... 54
9.6 DATA AND SAFETY MONITORING PLAN (DSMP) ... 54
10 DATA COLLECTION, SITE MONITORING, AE REPORTING. ... 56
10.1 RECORDS TO BE KEPT ... 56
10.1.1 Subject identifiers ... 56
10.1.2 Documentation ... 56
10.2 ROLE OF DATA MANAGEMENT ... 57
10.2.1 The Clinical Site responsibilities in data collection and management ... 58
10.2.1.1 Data Collection Protocol ... 58
10.2.1.2 Description & Flow of Case Report Forms (CRFs) ... 58
10.2.2 Data Security and Confidentiality ... 58
10.3 QUALITY ASSURANCE ... 58
10.3.1 Data Audit ... 59
10.4 ADVERSE EXPERIENCE REPORTING ... 59
11 HUMAN SUBJECTS ... 59
11.1 INSTITUTIONAL REVIEW BOARD REVIEW AND INFORMED CONSENT ... 59
11.2 SUBJECT CONFIDENTIALITY ... 59
11.3 STUDY MODIFICATION/DISCONTINUATION ... 59
12 PUBLICATION OF RESEARCH FINDINGS ... 60
13 REFERENCES ... 61
14 APPENDICES ... 70
14.1 ACRONYMS ... 70

SYNOPSIS

Hypoxic-ischemic encephalopathy (HIE) is a significant cause of neonatal encephalopathy. Although neonatal infection, stroke, and metabolic abnormalities can also cause NE, HIE accounts for about 60% of cases.[1] The incidence of HIE ranges from 1-7 per 1000 births in developed countries, depending on the definition used, with most estimates ranging between 1-3 per 1000. An estimated 12,000 infants are affected each year in the US.

Neonatal HIE accounts for 22% of annual neonatal deaths worldwide, totaling 814,000 deaths in 2008.[2] HIE is unpreventable in most cases, and therapies are limited. Hypothermia initiated within 6 hours improves outcome,[3-7] yet despite this therapy 44-53% of infants with HIE die or suffer moderate to severe disabilities including cerebral palsy (CP), intellectual disability, epilepsy, and visual impairment. When a normally developing fetus suffers brain injury leading to lifelong neurologic disability, there is excruciating heartbreak for all involved. Each year, infants who develop CP as a consequence of neonatal HIE will impose an economic burden of $1.7 billion in their lifetime medical and non-medical costs in the U.S. This figure does not include the costs incurred by children with cognitive impairment, epilepsy, visual impairment and other adverse neurodevelopmental outcomes due to HIE. Additional neuroprotective therapies are urgently needed.

The hematopoietic cytokine erythropoietin (Epo) has remarkable neuroprotective and neuroregenerative effects in the brain in animal models of neonatal brain injury.[8-13] Over 70 pre-clinical studies have tested the neuroprotective effects of Epo following hypoxic-ischemic brain injury, and these have produced impressive histologic and functional evidence for benefit ranging from 40 to 78% improvement in infarct size.[11,14-20] In non-human primates, Epo reduces the rate of CP and improves neurologic function in animals undergoing hypothermia for HIE.[21]

Two clinical trials suggested that infants with HIE treated with 5-7 doses of Epo experienced improved neurologic outcomes compared to infants who received placebo.[22,23] In a pilot phase I study, we found that Epo was safe, exhibited desirable pharmacokinetics, and resulted in a surprisingly low rate of moderate/severe disability even among infants with significant brain injury seen on MRI.[24] In a phase II study, we found that infants randomized to receive multiple doses of Epo had less brain injury on MRI and better 12-month motor outcomes than those who received placebo.[25] Given the compelling preclinical data, the suggestive findings from human trials, the favorable safety and pharmacokinetic data, and the unacceptable rate of adverse long-term neurologic outcomes in HIE, we will now perform a phase III trial to determine whether high-dose Epo improves neurodevelopmental outcomes following HIE.

1 Study Objectives

We *hypothesize* that high-dose Epo given to cooled infants with moderate/severe HIE will reduce the primary outcome of death or neurodevelopmental impairment (mild, moderate or severe) at 24 months of age from 49% to 33% or less. We further hypothesize that neonatal Epo will be safe, will decrease brain injury severity on neonatal MRI, and will decrease serial inflammatory cytokines and biomarkers of brain injury. We expect this finding will change clinical practice. In a randomized, double-masked, placebo-controlled efficacy trial of 500 infants with HIE, we have **three specific aims**, which will be evaluated by the endpoints detailed below:

- Aim 1: Efficacy
- Aim 2: Safety
- Aim 3: Biomarkers of neonatal brain injury

1.1 SPECIFIC AIMS

1.1.1 Aim 1: Efficacy

To determine whether Epo therapy (1000 U/kg (birth weight) given intravenously (IV) on Study Days 1, 2, 3, 4, and 7) reduces the composite primary outcome of death or neurodevelopmental impairment at 24 months of age. Since death is a competing outcome, it is critical to include it in the primary outcome measure.

Neurodevelopmental impairment (mild, moderate, or severe) is defined as any of the following:

- Gross Motor Function Classification System (GMFCS) level ≥1, or
- GMFCS = 0 or 0.5 AND CP (any type), or
- Bayley Scales of Infant and Toddler Development 3rd Edition (Bayley III) Cognitive Score <90

Secondary analyses: we will examine the effect of Epo on CP, severity of motor impairment, Bayley III cognitive and language scores, epilepsy, and behavioral abnormalities. We will also evaluate how Epo shifts the distribution of outcomes by evaluating a 4-level outcome measure: 1) normal; 2) mild motor and/or cognitive impairment; 3) moderate/severe motor and/or cognitive impairment; and 4) death.

Overall severity will consist of the *worst* severity observed in either motor or cognitive outcomes. Severity of motor impairment is defined by type of CP and GMFCS level. Severity of cognitive impairment is determined by Bayley III cognitive score, where Severity is defined as:

Severe: <70
Moderate 70-84
Mild: 85-89
None: ≥90

1.1.2 Aim 2: Safety

To establish the safety of high dose Epo treatment in the setting of HIE and hypothermia, by comparing Epo-related safety measures in infants treated with Epo vs. placebo.

1.1.3 AIM 3: Biomarkers of Neonatal Brain Injury

Aim 3a. To determine whether early high dose Epo decreases the severity of brain injury as evidenced by early neonatal MRI and MR spectroscopy.

Aim 3b. To determine whether early high dose Epo decreases the severity of brain injury as evidenced by serial circulating biomarkers of inflammation and brain injury.

1.2 ENDPOINTS

Primary Efficacy Endpoint

1. *Death or neurodevelopmental impairment at 24 months of age, compared between the two groups.*

Secondary Efficacy Endpoints

2. *Comparison of CP, motor impairment, Bayley III cognitive and learning scores, epilepsy, and behavioral abnormalities at 24 months, compared between the two groups.*

Safety Endpoints

3. *Rates of Epo-related reported adverse events through hospital discharge, compared between the two groups.*
4. *Rates of Epo-related reported adverse events through 24 months, compared between the two groups.*

Exploratory Analyses of Biomarkers of Neonatal Brain Injury

5. *Evaluation of serial circulating biomarkers of inflammation/brain injury, and MR evidence of brain injury obtained during the first week of life, compared between the two groups.*
6. *Evaluation of predictive values of serial circulating biomarkers of inflammation/brain injury, and MR evidence of brain injury obtained during the first week of life, for predicting 24 -month neurodevelopmental outcomes.*

1.3 OVERVIEW OF STUDY DESIGN

1.3.1 Definition of Study Day

To ensure study procedures are consistent across sites, the following definition will be used:

Study Day: Study Day 1 is defined as the calendar day on which Intervention 1 (first administration of study drug) occurs. If the study drug is first administered on the calendar day following the day of birth, then the day of birth will be called Study Day 0.

We propose a multicenter, randomized, double-masked, placebo-controlled Phase III clinical trial. We will enroll 500 infants at a minimum of 15, and a maximum of 30 sites. Patients will be randomized to receive either IV Epo 1000 U/kg/dose based on birth weight, or an equal volume of IV normal saline (NS). Study drug will be administered on study days 1, 2, 3, 4, and 7. All enrolled infants will receive standard clinical care including 72 hours of therapeutic hypothermia. The initial neonatal intervention and safety monitoring will begin at time of study consent (prior to 24 hours of age), and will be continued to hospital discharge. Follow-up evaluations will be performed at 4, 8, 12, 18, and 24 months via phone questionnaire, and at 24 months via in person neurodevelopmental examination.

Primary endpoint at age 24 months ± 56 days (i.e., ± 2 months) will be determined by presence of death or neurodevelopmental impairment.

Blood samples will be obtained from *all subjects* at 3 time points: baseline, Study Day 2, and Study Day 4. Urine samples will be obtained from *all subjects* at 2 time points: after consent on Study Day 0-1, and after completion of rewarming on Study Day 3-4. We will measure a baseline plasma Epo concentration prior to the administration of the first study drug dose. A subset of 200 patients identified post-hoc after meeting pre-defined criteria for quality of MRI/MRS data will have their samples measured for circulating and urinary biomarkers of brain injury and inflammation. All infants will have a brain MRI and MRS, ideally between 96 and 144 hours post-birth, as part of the standard clinical management of cooled infants. The study neuroradiologists will perform masked centralized reading of these neonatal brain MRI and MRS scans.

Sample Size and Population. We will enroll 500 infants ≥ 36 weeks gestational age with ***moderate or severe*** HIE. Eligible subjects (≥ 36 weeks of gestation, admitted to a participating site Neonatal Intensive Care Unit for therapeutic hypothermia) must meet the following criteria from 1 *AND* 2.

1. *Perinatal depression = at least one of the following:*
    a. *Apgar < 5 at 10 minutes of age, or*
    b. *Need for resuscitation at 10 minutes (i.e., chest compressions, or positive pressure respiratory support including endotracheal, mask ventilation, or CPAP), or*
    c. *pH < 7.00 in a cord gas (arterial or venous) or in an infant gas (arterial or venous) obtained at < 60 minutes of age, or*
    d. *Base deficit ≥ 15mmol/L in a cord gas (arterial or venous) or in an infant gas (arterial or venous) obtained at < 60 minutes of age*
2. *Moderate/severe encephalopathy = at least 3 of 6 modified Sarnat criteria present between 1-6 hours of age (see* ***Table 4****).*[3,5,26]

Assuming 10% loss of follow-up at 24 months, we will enroll 500 infants, in order to evaluate 450 infants (225 in each arm) at 24 months of age. We plan to apply for additional funding to allow us to follow these children to age 8.

Table 1. Study Procedure Chart

<table>
<tr><th rowspan="2">Age</th><th rowspan="2"><24 hours</th><th rowspan="2">Entry/ Day 1*</th><th colspan="6">Study Day</th><th rowspan="2">Discharge</th><th colspan="5">Months</th></tr>
<tr><th>2</th><th>3</th><th>4</th><th>5</th><th>6</th><th>7</th><th>4</th><th>8</th><th>12</th><th>18</th><th>24</th></tr>
<tr><td>Informed consent</td><td>X</td><td></td><td></td><td></td><td></td><td></td><td></td><td></td><td></td><td></td><td></td><td></td><td></td><td></td></tr>
<tr><td>Randomization</td><td>X</td><td></td><td></td><td></td><td></td><td></td><td></td><td></td><td></td><td></td><td></td><td></td><td></td><td></td></tr>
<tr><td>Epo or placebo IV</td><td></td><td>X</td><td>X</td><td>X</td><td>X</td><td></td><td></td><td>X</td><td></td><td></td><td></td><td></td><td></td><td></td></tr>
<tr><td>Hypothermia</td><td colspan="3">X</td><td></td><td></td><td></td><td></td><td></td><td></td><td></td><td></td><td></td><td></td><td></td></tr>
<tr><td>Blood sample</td><td>X</td><td></td><td>X</td><td></td><td>X</td><td></td><td></td><td></td><td></td><td></td><td></td><td></td><td></td><td></td></tr>
<tr><td>Urine sample</td><td colspan="2">X</td><td></td><td colspan="2">after rewarming</td><td></td><td></td><td></td><td></td><td></td><td></td><td></td><td></td><td></td></tr>
<tr><td>MRI/MRS</td><td></td><td></td><td></td><td></td><td colspan="2">96-144 hours post-birth</td><td></td><td></td><td></td><td></td><td></td><td></td><td></td><td></td></tr>
<tr><td>Sarnat exam</td><td>X</td><td></td><td></td><td></td><td></td><td>X</td><td></td><td></td><td></td><td></td><td></td><td></td><td></td><td></td></tr>
<tr><td>Phone interview</td><td></td><td></td><td></td><td></td><td></td><td></td><td></td><td></td><td></td><td>X</td><td>X</td><td>X</td><td>X</td><td>X</td></tr>
<tr><td>Neurodevelopmental exam</td><td></td><td></td><td></td><td></td><td></td><td></td><td></td><td></td><td></td><td></td><td></td><td></td><td></td><td>X</td></tr>
<tr><td colspan="15">* Study Day 1 is defined as the calendar day on which Intervention 1 is administered. If the study drug is administered on the calendar day following the day of birth, then the day of birth will be called Study Day 0.</td></tr>
</table>

<table>
<tr><th></th><th colspan="12">Months</th></tr>
<tr><td>Age</td><td>30</td><td>36</td><td>42</td><td>48</td><td>54</td><td>60</td><td>66</td><td>72</td><td>78</td><td>84</td><td>90</td><td>96</td></tr>
<tr><td>Extended Contact phone calls</td><td>X</td><td>X</td><td>X</td><td>X</td><td>X</td><td>X</td><td>X</td><td>X</td><td>X</td><td>X</td><td>X</td><td>X</td></tr>
</table>

Each of the enrolling sites has extensive experience with neonatal clinical research, performs passive or active hypothermia during transport, performs whole body cooling, and has an active neonatal follow-up program.

2 BACKGROUND

2.1 RATIONALE

In 2008, it is estimated that birth asphyxia (lack of oxygen and perfusion to the brain and other vital organs) caused 814,000 deaths worldwide, and contributed to 22% of neonatal deaths. Hypoxic-ischemic brain injury remains a significant problem in the U.S., affecting 1-3 per 1000 births.[1,27-29] Therapeutic hypothermia has proven to be neuroprotective, but neonates who received hypothermia in clinical trials still experience unacceptably high rates of death (mean 28%, range 24-38); cognitive dysfunction (24%, range 21-25); CP (22%, range 13-28) and death or moderate/severe disability (48%, range 44-53).[4-7,30] Other neurologic disabilities after HIE despite hypothermia include epilepsy (19%, range 15-24) and cortical visual impairment (6%, range 1-10). New complimentary therapies, to further improve outcomes, are desperately needed, and these must be tested in the context of therapeutic hypothermia. Epo is one such promising neuroprotective therapy, with compelling preclinical data, supportive phase I and II clinical trials, and a reassuring safety profile in neonates. The pharmacokinetics of Epo in the setting of HIE and hypothermia have been defined,[31] and it is now ready for evaluation of safety and efficacy in a phase III trial.

2.1.1 Patient population

Newborn infants 36 weeks of gestation or greater, with laboratory and physical exam findings compatible with moderate or severe HIE who have started therapeutic hypothermia by 6 hours of age. Male and female infants of all races and ethnicities cared for at participating centers are eligible.

2.1.2 Mechanisms of Epo Neuroprotection

Epo, a glycoprotein originally identified for its role in erythropoiesis, has neuroprotective and reparative effects in the central nervous system (CNS).[32-37] Epo functions by binding to its homodimeric cell surface receptor (Epo-R). Epo-Rs are expressed by a variety of cell types in the CNS,[38,39] including neuronal progenitor cells,[35] subsets of mature neurons,[40] astrocytes,[41] oligodendrocytes,[42] microglia,[43] and brain endothelial cells.[35] Epo and Epo-R expression in the brain is high during fetal development but declines rapidly after birth. In the setting of hypoxia ischemia, Epo-R expression in neurons, astrocytes, and microglia is rapidly upregulated. Increased Epo expression follows, mediated via hypoxia-mediated stabilization of neuronal transcription factor hypoxia-inducible factor-1α, if the insult is of sufficient duration.[38,39,44] In the absence of Epo-Epo-R binding, cells are predisposed to apoptosis, while in the presence of Epo, cells are preserved.[80,87] This creates an important rationale for exogenous Epo administration, given that brain injury can occur after brief but catastrophic insults such as placental abruption or cord accidents, which are insufficient to stimulate an increase in Epo synthesis.[88]

Epo signaling targets several <u>acute intracellular mechanisms</u> important in newborn hypoxic-ischemic brain injury.[45] Epo exerts direct neuroprotective effects on neurons by activating anti-apoptotic pathways,[46-48] but also decreases inflammation,[49,50] increases anti-oxidant activity,[51,52 53] and reduces excitotoxic cell injury.[54] Epo protects neuronal progenitor cells from interferon-γ, lipopolysaccharide (LPS) and hypoxic-ischemic injury,[18,55,56] protects oligodendrocytes and improves white matter survival assessed by MRI and pathologic analysis. [18,57,58]

In addition to acute effects, Epo promotes <u>neurogenesis and long-term repair</u>.[59-62] *In vitro*, Epo increases the number of newly generated neuronal precursor cells and directs stem cells to differentiate into neurons.[41,63,64] Epo also stimulates production of growth factors such as vascular

endothelial growth factor (VEGF), brain-derived neurotrophic factor (BDNF), and glial cell derived neurotrophic factor (GDNF) that have important neurogenic and pro-angiogenic effects.[35,65] Overall, Epo not only decreases cell death but also enhances neurogenesis and angiogenesis in a number of *in vitro* and *in vivo* models of brain injury, which may be necessary for long-term improvement in brain histology and functional performance.[17,59-62,66]

Epo signaling is mediated by binding to Epo-R and activation of several important intracellular signaling pathways, including the phosphatidylinositol 3-kinase (PI3K)/Akt, Janus kinase 2 (JAK2)/STAT5, and extracellular signal-regulated kinase (ERK)1/2 pathways. Akt limits inflammation,[67] decreases apoptotic cell death and increases angiogenesis,[68] while STAT5 plays a role in cell survival.[69] The ERK pathway has anti-apoptotic and anti-inflammatory effects *in vitro* and is critical in neurogenesis and cell fate commitment.[70,71]

2.1.3 Pre-clinical studies of Epo in neonatal HI brain injury

Over 70 pre-clinical studies have tested the neuroprotective effects of Epo following hypoxic-ischemic brain injury, and these have produced overwhelming histologic and functional evidence for benefit.[10,20,72] Pre-treatment with Epo prior to hypoxic-ischemic injury is neuroprotective in neonatal rodents.[73-78] A single dose of Epo administered immediately after hypoxia-ischemia in the P7 rat (comparable to human infant at term[79]) significantly reduces infarct volume, improves short-term spatial memory[19] and decreases hemispheric brain loss 6 weeks after injury, with increased neurogenesis seen in the subventricular zone.[17] Multiple doses of Epo after neonatal stroke reduce infarct volume in a dose-dependent manner,[15] with improvement in sensorimotor function.[16] Three doses of Epo given after neonatal brain injury result in improved histologic outcomes at 1 week,[80] and marked preservation of neurologic function at 3 months, including improved learning and memory, swim speed, balance and forepaw use, as well as improved hemispheric and regional brain volumes.[81] Epo results in decreased neuronal loss and learning impairment following neonatal hypoxic ischemic brain injury.[11,14] When Epo therapy is initiated as late as 48 hours after hypoxic-ischemic event, one sees improved behavioral outcomes, enhanced neurogenesis and reduced white matter injury at 14 days.[18,59] In a rat model of focal ischemic injury, we recently found that Epo 1000 U/kg starting as late as 7 days after injury leads to significant preservation of brain volume and improved sensorimotor function.[82] This finding emphasizes the late neuroregenerative effects of Epo. Finally, in non-human primates, Epo reduces the rate of CP and improves neurologic function in animals undergoing hypothermia for HIE.[21]

2.1.4 Dosing regimen

We will administer 5 IV doses of Epo 1000 U/kg or NS. The first dose will be given at < 24+2 hours of age, and subsequent doses on study days 2, 3, 4, and 7. Our dosing regimen is justified by the following points, each of which is expanded upon below:

- <u>Multiple doses</u> over 1 week produce maximal neuroprotection (see 2.1.4.1)
- <u>Epo 1000 U/kg IV produces total drug exposure (AUC) and maximum concentration levels</u> that produce optimal neuroprotection in animal models (see 2.1.4.2)
- <u>Epo 1000 U/kg falls within the range of doses</u> (500-2500 U/kg) reported in clinical studies to improve outcomes in neonatal HIE[22,23] (see 2.2.1)
- <u>Epo 1000 U/kg IV appears well-tolerated</u> in newborn infants (see 2.2.2)

2.1.4.1 Why multiple and late doses?

Pre-clinical studies show that multiple doses of Epo administered over 1-2 weeks produce maximal neuroprotection. Whereas a single Epo dose had no long-term histological or behavioral effects after neonatal ischemia injury, rats receiving 3 doses of Epo (immediately after injury, 24 hours, and 7 days after injury) demonstrated improved function and regional brain volumes into adulthood.[81] Further, a multiple dose Epo treatment protocol initiated one week following injury in the rat model was associated with significant behavioral improvements and preservation of brain volume.[82] These data provide the rationale for a dose at 7 days following injury, or prior to discharge. Prolonged administration of Epo increases oligodendrogenesis and neurogenesis while decreasing astrogliosis.[18] Rats given multiple doses of Epo received a smaller cumulative dose than did the single dose treated rats, suggesting that neuroprotection and repair are dependent on a prolonged course of administration. Multiple-dose Epo treatment protocols that are initiated between 48 hours and 7 days after early brain injury also improve short-term sensorimotor performance and decrease brain injury, increasing the number of oligodendrocyte precursor cells, and enhancing the reorganization of white matter in both mouse and rat models of immature or mature brain injury.[13,82,83]

2.1.4.2 Dose selection

Dose selection was informed by our pilot Phase I and Phase II pharmacokinetic data.[31,84] Among infants being cooled for HIE, Epo 1000 U/kg IV produced overall exposure (AUC) and maximum concentration levels that most closely mimic established neuroprotective levels from preclinical studies.[31]

Sandra Juul (CCC Multi-PI) has shown that multiple doses of Epo 5000 U/kg administered subcutaneously (SC) or intraperitoneally (IP) afford the greatest amount of neuroprotection in a rodent model.[80] At this dose, the mean AUC ranged from 117,677 (SC) to 140,331 Uh/L (IP) and the mean Cmax ranged from 6,224 U/L (SC) to 10,015 U/L (IP)[85]

From our phase I study (see 2.2.3),[84] we know that Epo 1000 U/kg IV, given in conjunction with hypothermia, produces drug exposure levels (AUC 131,054 ±17,083 Uh/L and Cmax 13,780 ± 2,674 U/L) that most closely mimic the target neuroprotective levels described above. In contrast, Epo 500 U/kg produced insufficient plasma elevations, and doses of 2500 U/kg produced AUC and Cmax values that exceeded the optimal neuroprotective range by 3-fold.

Although the upper safety limit of Epo is unknown, doses ranging from 300 to 3,000 U/Kg/dose have been studied in randomized controlled trials of preterm and term neonates with no resulting safety issues. Preclinical data suggest that too much Epo (30,000 U/kg/dose) can lead to diminished efficacy,[80] and may be harmful.[86] Epo 1000 U/kg/dose IV is a moderately high dose that establishes Epo exposure levels within the optimal neuroprotective range, while also minimizing risks associated with giving too much Epo.

Low doses of Epo (200-400 U/kg) used to treat anemia do not raise CSF Epo concentrations in the conditions of an intact blood brain barrier, however, high dose

Epo administered systemically has been shown to increase measured Epo in the CSF or brain tissues of both experimental models and human infants, particularly in the setting of acute brain injury.[22,85,87-91] In a phase II trial, we confirmed that for neonates with HIE receiving hypothermia, Epo 1,000 U/kg every 24 hours resulted in consistent achievement of target exposures associated with neuroprotection in animal models.[31]

In summary, Epo 1000 U/kg/dose IV produces drug exposure levels that afford optimal neuroprotection. This dose is likely to minimize risks associated with giving too much Epo, especially in the setting of hypothermia, which may slow drug clearance (see 2.2.3).

2.1.4.3 Route of administration

We will administer Epo IV. Our pilot data[84] suggest that Epo 1000 U/kg given IV is safe and provides pharmacokinetics consistent with neuroprotective effects in animal models (i.e., rats and monkeys). For patients who have lost IV access, SC administration will be permitted.

2.1.5 Need, relevance and priority for the study

HIE affects up to 12,000 infants annually in the U.S., and accounts for 22% of neonatal deaths worldwide, totaling 814,000 deaths in 2008.[2] Although hypothermia improves outcomes, 44-53% of affected newborns die or experience neurologic disability despite this therapy.[3-7,30] New treatments are urgently needed. Epo has neuroprotective actions and regenerative effects that go beyond the effects of hypothermia alone.

The proposed research has the potential to significantly reduce suffering from life-long neurologic disabilities, and to lead to enormous societal cost savings. In 2012 currency, the lifetime cost of CP is estimated at 1.15 million dollars per affected individual.[92] Using a conservative estimate of 20% CP rates in infants with HIE and hypothermia,[3,5,7,93] and a conservative HIE incidence of 2 per 1000, each year babies born with HIE introduce an economic burden that will ultimately total $1.7 billion in lifetime costs due to CP alone. Similar calculations using CDC cost data[92] and rates of disability derived from hypothermia studies[3,5,7] suggest that each year, HIE produces additional lifetime costs of $1.6 billion for intellectual disability. If no benefits of Epo are found, the trial will nevertheless generate important information about clinical and laboratory antecedents of neurodevelopmental disabilities due to newborn brain injury, and the data will inform the next generation of neonatal neuroprotection trials.

2.2 SUPPORTING DATA

2.2.1 Clinical studies of Epo and HIE

To date, five phase I or II studies have suggested that Epo improves outcomes after HIE.[22,23,94]

In an Egyptian study, 15 infants were given 5 daily SC doses of Epo 2500 U/kg starting at 1 day of age.[23] Compared to placebo controls, those who received Epo had improved electroencephalogram (EEG) backgrounds and reduced biomarkers of oxidative stress at 2 weeks of age, and better neurodevelopment at 6 months of age.

In a randomized trial performed in China, researchers treated 84 infants with HIE with Epo 300-500 U/Kg IV every other day for two weeks. Compared to infants who received placebo, those who received Epo were less likely to die or have moderate to severe disability at 18 months of age (44% vs. 25%, P=0.02).[22] No side effects of Epo therapy were seen.

The Egyptian and Chinese studies are limited by small size, inadequate masking, deviation from intention to treat analysis, and lack of hypothermia. There is currently insufficient evidence to support the clinical use of Epo in treating HIE.

In our **phase I pilot trial** of Epo for HIE, neurodevelopmental outcomes were available for 22 of 24 treated infants.[24] For 22 infants who received multiple doses of Epo at 250, 500, 1000, or 2500 U/kg/dose, mean age at last assessment was 22 months (range 8-34). There were no deaths. Eight (36%) had moderate to severe brain injury on neonatal MRI. Moderate to severe disability occurred in only 1 child (4.5%).[24]

In a phase II randomized, multi-center, double-masked, placebo controlled trial of Epo for HIE (NEATO, Wu PI), 50 newborns undergoing hypothermia for moderate/severe HIE were randomized to Epo 1000 U/kg/dose or placebo (NCT# 01913340). Infants who were randomized to receive Epo exhibited less brain injury on MRI and better 12-month motor outcomes than those who received placebo.[25] Further studies are needed to definitively establish the efficacy of Epo in treating HIE.

2.2.2 Safety

Reported Epo side effects in adults with renal failure include hypertension, thrombosis, red cell aplasia, myocardial infarction, stroke, congestive heart failure, seizures, tumor progression and increased death.[95-99] It is important to note that these side effects have been observed with long term chronic therapy, and none of these side effects have been reported in neonates.

Based on the adult experience, potential risks of Epo are listed in the Consent form as follows:

Rare but serious:

- Increased blood pressure (reported in adults; not reported in infants)
- Increased clotting (reported in adults; not reported in infants)
- Increased risk of seizures (reported in adults on dialysis; not reported in infants)
- Increased risk of death (reported in adults with cancer or stroke; not reported in infants)
- Polycythemia (reported in adults with long term use; not reported in infants)

There is an extensive literature of Epo therapy in preterm infants. Between 1991 and 2006, over 2400 infants were enrolled in 30 randomized controlled trials of Epo for anemia of prematurity, with Epo therapy ranging from 70 to 5000 U/kg/week (35 – 750 U/kg/dose) lasting 2 weeks to several months in duration.[100] Although a concern has been raised that chronic Epo therapy in infants under 32 weeks of gestation might increase the risk of retinopathy of prematurity[101] and skin hemangiomas,[102] these findings have not been reported in prospective randomized controlled trials in preterm or term infants. [103,104]

No adverse events have been reported in prospective studies of high-dose Epo in neonates:

1. Phase II studies of HIE: **300-2500** U/kg Epo (N=182) (see 2.2.1)[22,23,25,94,105]
2. Phase I study of HIE: up to 6 doses of Epo **250–2500** U/kg (N=24)[84] (see 2.2.3)
3. Phase III study of preterm infants (ongoing): 6 doses of **1000** U/kg followed by maintenance doses of 400 U/kg three times a week until 32-6/7 corrected weeks of gestation (N=941) (PI Juul, PENUT study)[106]

4. Phase III study of HIE (ongoing): 3 doses of Epo **1000 U/kg** (n=115) (Patkai PI, Neuroepo study)
5. Phase I/II study of preterm infants: 3 daily doses **500**-**2,500** U/kg (N=30)[107]
6. Phase III trial of preterm infants: 3 daily doses **3000** U/kg (N=30)[108]
7. Phase I/II study of congenital heart disease: 3 daily doses **1000** U/kg (N=33)[109]
8. Phase II study of perinatal stroke: 3 daily doses **1000** U/kg (N=25)[110]
9. Phase II of preterm infants: **400** U/kg 3 times a week (N= 56)[111,112]
10. Phase III of preterm infants: 3 daily doses **3000** U/kg (N=448)[108,113]

Therefore, high-dose Epo appears to be safe when administered to neonates in the first 1-2 weeks after delivery. Epo 1000 U/kg/dose falls well within the range of high dose Epo treatment regimens that have been studied in a number of neonatal neuroprotection trials, none of which have raised concerns regarding safety. Polycythemia is a theoretical concern, but our experience in term HIE (see 2.2.3) and in preterm infants[107] suggests that hematocrit in fact decreases over time because of phlebotomy losses in these critically ill infants.

2.2.3 Pilot studies

Phase I dose escalation and pharmacokinetic study (IND 102,138)

We performed a phase I study of the safety and pharmacokinetics of Epo + hypothermia.[84] This was a multicenter, open label, dose escalation study involving 5 sites (UCSF, Oakland Children's Hospital, University of Washington, Children' National Medical Center, Santa Clara Valley Medical Center). Patients had moderate/severe HIE with acidosis (mean cord pH = 6.84, SD 0.14), neonatal resuscitation > 10 minutes (88%) or 10 minute Apgar < 5 (63%).

We tested 4 doses of Epo IV: 250 (N=3), 500 (N=6), 1000 (N=7) and 2500 U/kg/dose (N=8). Patients received up to 6 doses every 48 hours, with the first dose given within 24 hours of age. Average age at consent was 15.4 hours (SD 5.7), and average length of hospital stay was 13.5 days (SD 7.2). Patients received an average of 4.8 doses. Reasons for not completing the 6-dose regimen include being discharged from the hospital and loss of IV access.

Epo followed nonlinear pharmacokinetics, consistent with previous reports.[85,114,115] As the dose of Epo increased 4 and 10-fold (from 250 to 1000 and 2500 U/kg), the overall exposure to circulating Epo (AUC) increased 7.1 and 17.8 times, respectively (Table 2). However, there was no excessive accumulation of drug following multiple doses, at any of the doses studied. Steady-state plasma Epo concentrations were attained by the second dose for all four dosages, and peak and trough concentrations were stable across doses. Plasma Epo concentrations demonstrated fairly limited variability across individual patients, with an average coefficient of variation of 26% for Cmax. Compared to premature infants given identical doses of IV Epo,[107] our patients demonstrated about a 2-fold reduced rate of Epo elimination. Possible explanations for the slower drug elimination observed include hypothermia treatment, renal compromise, older gestation, hypoxia-ischemia, or a combination of these factors.

Epo 1000 U/kg achieved a mean AUC and Cmax levels that most closely mimic the target neuroprotective levels derived from pre-clinical studies (Table 2). In contrast, Epo 500 U/kg/dose produced insufficient plasma elevations, and doses of 2500 U/kg produced AUC and Cmax values that exceeded the optimal neuroprotective range by about 3-fold.

Table 2. Single dose pharmacokinetic parameters of 4 different Epo doses given in conjunction with hypothermia, in neonates with HIE.

PK parameter	250 U/kg (n=3)	500 U/kg (n=6)	1000 U/kg (n=7)	2500 U/kg (n=8)	Target level*
AUC ([U*h]/L)	18426 ± 8976	50306 ± 7426	131,054 ± 17,083	328002 ± 61945	117,677-140,331
Cmax (mU/mL)	3156 ± 1615	7046 ± 814	13,780 ± 2,674	33316 ± 7377	6,224 - 10,015
Cl (ml/h/kg)	15.6 ± 6.3	10.1 ± 1.5	7.7 ± 0.9	7.9 ± 1.5	
t ½ (h)	7.6 ± 6.9	7.2 ± 1.9	15.0 ± 4.5	18.7 ± 4.7	

*Target levels are AUC and Cmax levels that produce optimal neuroprotection in animal models.

No deaths or serious adverse effects were noted in the pilot trial. No patients experienced polycythemia. Mean hematocrit decreased from 45.6 to 41.5% between day 1 and final day of testing (5-14 days). Brain MRI performed at a median of 6 (range 4-13) days of age revealed no intracranial hemorrhages or sinovenous thromboses. MRI was normal in 13 (54%), demonstrated watershed injury in nine (42%), basal ganglia injury in one (4%), and focal arterial infarction in one (4%). Among infants with HIE who have undergone hypothermia, normal MRI/MRS scans have been reported in 17-54%.[116-118] Outcomes were available for 22 of 24 infants. For these 22 infants, mean age at last assessment was 22 months (range 8-34). There were no deaths. Eight (36%) had moderate to severe brain injury on neonatal MRI. Moderate to severe disability occurred in 1 child (4.5%), in the setting of moderate to severe basal ganglia/thalamic injury.

Figure 1. Epo pharmacokinetics in 24 infants undergoing hypothermia for HIE.

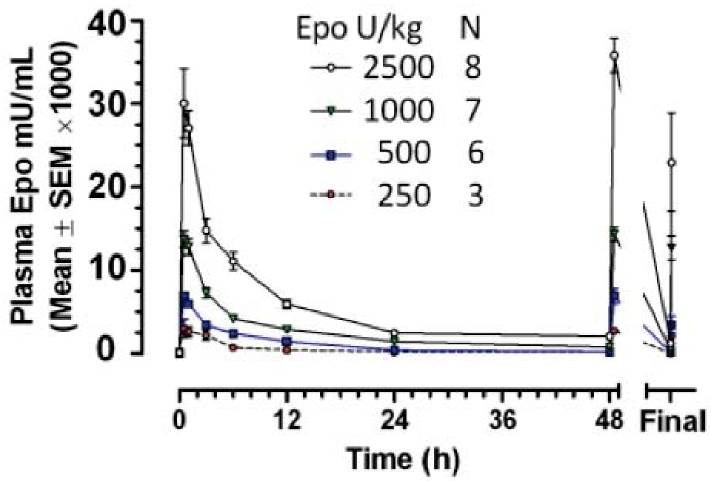

Phase II Randomized Controlled Trial of Epo for HIE (NEAT-O study, IND 102,138)

In a phase II randomized, multi-center, double-masked, placebo controlled trial of Epo for HIE (NEATO, Wu PI), 50 newborns undergoing hypothermia for moderate/severe HIE were randomized to Epo 1000 U/kg/dose or placebo (NCT# 01913340). Consent rate was 79%, and enrollment was completed ahead of schedule. The first study drug dose was given at mean 16 hours of age. Follow up rate at 6 months of age was 97%. No safety concerns were raised by the Data Safety Monitoring Board (DSMB).

Our phase II trial (N=50) was not designed to show efficacy; yet the results are promising.[25] **Neonatal brain MRI** performed at mean 5.1 (SD 2.3) days was independently reviewed by 2 masked evaluators, and scored using the Washington University MRI scoring system.[119] The MRI brain injury severity score was significantly lower in the Epo-treated than placebo group (4.0 vs. 16.4, P = 0.003). Similarly, moderate/severe brain injury was less common (5% vs. 44%), and normal brain MRIs were more common (36% vs. 12%) in infants who received Epo vs. placebo. Subcortical injury (i.e., injury to the basal ganglia, thalamus or posterior limb of the internal capsule), a well-known predictor of adverse neurologic outcome,[117,120] was significantly reduced in the Epo treated group (36% vs. 68%, P=0.04). At 12 months of age, we assessed development using the Alberta Infant Motor Scale (AIMS) and the Warner Initial Developmental Evaluation of Adaptive and Functional Skills (WIDEA),[121,122] a questionnaire that assesses self-care, motor function, communication and social cognition in young infants and children. Infants who received Epo scored better on the AIMS score (53.2 vs. 42.8, P=0.03), and on the WIDEA motor subscore (28.6 vs. 23.8, P=0.05) than infants who received placebo.[25]

3 STUDY DESIGN

Overview: We propose a multicenter, randomized, double-masked, placebo-controlled trial to test the hypothesis that Epo given to infants with moderate-severe HIE will safely lead to improved neurodevelopmental outcome at 24 months. We will enroll 500 newborns ≥ 36 weeks gestational age with moderate/severe HIE determined by neurologic symptoms, Apgar score, level of acidosis, and need for resuscitation. Study drug (Epo or placebo) will be given IV on study days 1, 2, 3, 4, and 7.

Primary outcome will be assessed at 24 months. The primary endpoint is the composite of death or neurodevelopmental impairment. Impairment (mild, moderate, or severe) is defined as of any of the following: 1) GMFCS level ≥1, or 2) GMFCS = 0 or 0.5 AND CP (any type), or 3) Bayley III <90. Since death is a competing outcome, it is critical to include it in the primary outcome measure.

Secondary analyses at age 24 months: we will assess the effect of Epo on a) presence of CP, b) severity of motor impairment, c) Bayley III cognitive and language scores, d) epilepsy (i.e., ≥ 2 afebrile, unprovoked seizures), and e) behavioral abnormalities (i.e., attention problems or aggressive behavior) based on the Child Behavior Checklist (CBCL) externalizing score.

Additional exploratory analyses include the effect of Epo on sensory deficits that can result from HIE. We will collect 24-month data regarding the presence of cortical visual impairment diagnosed by an ophthalmologist, and hearing impairment requiring hearing aids. To elucidate the effect of Epo on all severities of impairment, we will perform a secondary analysis of the effect of Epo on the following 4-level outcome: 1) normal, 2) mild motor and/or cognitive impairment, 3) moderate/severe motor and/or cognitive impairment, and 4) death. Severity of motor impairment will be determined by type of CP and GMFCS level. Severity of cognitive impairment will be determined by the Bayley III Cognitive Score.

4 SELECTION AND ENROLLMENT OF SUBJECTS

4.1 INCLUSION CRITERIA

1. *Newborns ≥ 36 weeks gestational age*
2. *Receiving active or passive whole body cooling/hypothermia since < 6 hours of age*
3. *Perinatal depression based on at least one of the following:*
    a. *Apgar score < 5 at 10 minutes, or*
    b. *Need for resuscitation at 10 minutes (i.e., chest compressions, or positive pressure respiratory support including endotracheal, mask ventilation, or CPAP), or*
    c. *pH < 7.00 in a cord gas (arterial or venous) or in an infant gas (arterial or venous) obtained at < 60 minutes of age, or*
    d. *Base deficit ≥ 15 mmol/L in a cord gas (arterial or venous) or in an infant gas (arterial or venous) obtained at < 60 minutes of age*
4. *<u>Moderate to severe encephalopathy</u>, based on presence of <u>at least 3 of 6</u> Sarnat criteria present between 1-6 hours after birth:*
    a. *Reduced consciousness*
    b. *Decreased spontaneous activity*
    c. *Posture*
    d. *Tone*
    e. *Primitive reflexes (suck and Moro)*

f. Autonomic abnormality (pupils and respirations)

4.1.1 Prior therapy

All enrolled infants will have started hypothermia by 6 hours of age, which is standard of care at each of the study sites. Hypothermia will consist of whole body cooling at all study sites.

4.1.2 Demographic characteristics

Enrollment is irrespective of gender, race and ethnicity.

4.2 EXCLUSION CRITERIA

1. *Study drug unlikely to be administered within 26 hours of birth*
2. *Infant has living twin (or higher order multiple) who is also being cooled.*
3. *Birth weight < 1800 g (i.e., intrauterine growth restriction)*
4. *Genetic or congenital condition that affects neurodevelopment or requires multiple surgeries (e.g., congenital viral infection, hydrops, complex congenital heart disease, severe dysmorphic features, etc.)*
5. *Head circumference <30 cm*
6. *Redirection of care is being considered due to moribund condition*
7. *Patient anticipated to be unavailable for evaluation at age 2*
8. *Polycythemia (hematocrit > 65.0%)*
9. *Parents/legal guardians with diminished capacity or autonomy*[1]
10. *Infant is participating or intends to participate in another interventional study during the birth hospitalization (note: does not include observational studies)*
11. *Sentinel event and encephalopathy occurred only after birth*[2]
12. *Unable to consent in primary language of parent(s)*

4.3 STUDY ENROLLMENT PROCEDURES

4.3.1 Identification and recruitment

Infants ≥ 36 weeks gestational age who are undergoing hypothermia for HIE will be screened by the study investigators for eligibility. A study investigator will discuss the child's condition with the attending physician. With the attending physician's approval, the parents will be approached by a study investigator and/or study coordinator who will explain the study and obtain informed consent. Parents who are at an outside hospital may be given study written materials by the transport team, after which a study investigator may obtain consent by phone, given the time sensitive nature of the study. A brochure and consent form explaining the study in lay language will be provided to the family either in person or via fax if the parents are at an outside hospital.

[1] Please refer to your individual IRB requirements regarding permission to enroll an infant in the study if only one parent/legal guardian meets this exclusion criterion.

[2] Some infants may experience a sentinel event *after* birth leading to a poor blood gas before 1 hour of age, but have no prior signs of low Apgars, acidosis, prolonged resuscitation, or encephalopathy. These infants will be **excluded** from the study.

Once written consent is obtained (in person or by phone), the infant will be enrolled and randomized to receive Epo study drug or placebo. Consent rate is expected to be 80% or more.

4.3.2 Screening for eligibility and monitoring of recruitment targets

Data will be collected on all patients who are screened for enrollment. These data will include the inclusion/exclusion criteria in Sections 4.1 and 4.2 above, and hence confirm eligibility or provide the reasons for ineligibility, as well as reasons for nonparticipation of eligible subjects. Non-HIPAA related data on all screened patients will be submitted to the Data Coordinating Center (DCC), and summary reports will be reviewed by the site Principal Investigators (PIs) and Clinical Steering Committee. All sites are anticipated to enroll at least 12-20 infants a year. We will assess site enrollment on a monthly basis, and if enrollment at an individual site falls below expected levels (i.e., enrolling only 3 infants in 6 months, or 1.5 infants every 3 months), the Executive Committee and DSMB will evaluate performance. The multi-PI's will give the site warning, and if underperformance continues, the site may be dropped from the trial.

4.3.3 Consent procedures

A study investigator and/or coordinator will obtain parental informed consent. For out-born infants whose parent(s) are not physically present, we will obtain written consent over the phone. That is, if the parent(s) are at an outside hospital, we will provide them with a copy of the consent form, either from the Transport team, or via fax. The consenting study personnel will then read through the consent form with both parents (when possible) over the phone, or in person if possible. Consent will be documented according to local and national regulations.

4.3.4 Stratification, Randomization and Masking Procedures

The DCC will provide each site pharmacy with a randomization look-up table that pairs each study ID with a treatment allocation. Randomization is stratified by site and HIE severity level. See 6.3.2.1 for definitions of "moderate" and "severe" HIE, based on modified Sarnat score. The site pharmacy will then draw up the assigned study drug dose into a syringe, based on the randomization list. The appearance of the syringes of Epo and placebo are identical, thus the bedside staff remain masked. Only the research pharmacist and DCC biostatistician will be un-masked to treatment assignment. All parents, study and clinical staff, as well as all Clinical Coordinating Center (CCC) members, will remain masked to treatment assignment throughout follow-up. To ensure balance within clinical centers, the randomization will be implemented using a randomly permuted blocks design to ensure approximate balanced assignments within site and HIE severity level. The sequence of assignments for each site and stratum will be prepared in advance by the DCC.

After all final follow-up evaluations have been completed, which may be at age 8 or older if additional funding is obtained to follow subjects beyond 24 months of age, an effort will be made to inform participating families of the completion of the study, and families will be offered an opportunity to learn the treatment group assignment for their child.

5 STUDY INTERVENTIONS

5.1 STUDY DRUG ADMINSTRATION AND DURATION

Patients will be randomized to receive either IV Epo 1000 U/kg/dose (based on birth weight), or an equal volume of IV NS. Study drug (Epo vs. NS) will be administered on study days 1, 2, 3, 4, and 7, with the

same dose/volume used for each administration. We will administer Epoetin alpha for injection (4000 U/mL), manufactured by Amgen Inc., prepared and used according to the package insert. Study drug will be infused via IV push over 1-2 minutes, followed by 1 mL normal saline flush. If an institution does not allow medications to be given to neonates via IV push, study drug may be infused via pump infusion over 5 minutes, followed by 1 mL NS flush. Potential side effects of the drug administration include introduction of infection, and bleeding from the line site. All subjects will be treated in a level III or IV neonatal intensive care unit. The initial study drug dose should be given as soon as possible after randomization and no later than 24 +2 hours of age. Each subsequent dose should be given within +/- 2 hours of the time that the first dose was given (i.e., within a 4 hour window). This window provides some flexibility so that study drug does not have to be given during change of nursing shift, or while the infant is undergoing a procedure, for example. Patients will not be kept in the hospital solely for completion the study intervention. If patients are discharged from the hospital prior to day 7, they may receive the final study drug dose on the day of discharge, provided that it given at least 20 hours following the prior dose. If the patient loses IV access prior to receiving all study drug dose(s), then the remaining dose(s) may be given SC. That is, placement of a new IV for the sole purpose of giving study drug will not be required.

5.2 HANDLING OF STUDY DRUG

Each participating hospital will order Epo 4000 unit/mL, 1-ml vials, from a commercial wholesaler. Sites will generally purchase sufficient Epo to treat 2-4 patients randomized to the Epo arm. Each participating site pharmacy will be responsible for appropriately storing, accounting for and dispensing the study drug. Pharmacy accountability will be monitored closely. The research supply of Epo will be kept separate from other commercial stocks of Epo at the participating sites. Epo or placebo will be dispensed for IV administration as a patient-specific unit-dose syringe upon receipt of an investigator-physician order for a research subject. Depending on the subject's randomized treatment assignment, the un-masked pharmacy personnel will dispense a syringe containing the prescribed dose of Epo or an equivalent volume of 0.9% Sodium Chloride for Injection, USP (NS). Both Epo and placebo syringes will be labeled, packaged, transported, stored and administered in an identical manner so as to maintain the masking. Labeling will be adapted to meet local labeling requirements, but will not un-mask the drug assignment. The site pharmacies shall maintain accountability logs for all research supplies of Epo. Accountability logs will be reviewed by the DCC.

The sites will maintain accountability logs for the Epo inventory. These logs will include the purchase date (date received), lot number, expiration date, and quantity of all Epo purchased for the trial. In addition, every dose dispensed will also be recorded in a subject-specific accountability log. The Epo lot number for each dose dispensed will be recorded the inventory balance will also be noted on the accountability log. Individual subject drug accountability logs should be sent to the HEAL financial analyst on a regular basis and these logs will be reviewed by the DCC to ensure appropriate drug administration per the randomization log.

Epoetin alfa purchased for the HEAL trial will be kept refrigerated, separate from the regular medication inventory. The refrigerator will be monitored and alarmed and will have access to emergency backup power. Epoetin alfa vials must be stored in a refrigerator between 35.6°F to 46.4°F (2°C -8°C).

Epo will be labeled with stickers marked "For Investigational Use Only". The invoices for the purchase of the Epoetin will be retained at the local site in the same manner that a study drug shipping receipt would be retained.

Epo vials must be stored in the refrigerator between 36°F to 46°F (2°C to 8°C). **Do not freeze** vials and do not use a vial of Epo that has been frozen in the past. Keep vials away from direct light. **Do not shake Epo vials.** Single use vials of Epo should be used only one time. Only one dose of study drug may be drawn up at a time.

If the wrong study drug is administered to a subject (i.e., Epo is given to a Control patient, or normal saline is given to a Treatment patient), the error should be reported in a blinded manner to the CCC as a protocol violation, and all future doses should be corrected to the original randomization assignment. The study violation must also be reported to the local IRB per institutional policy.

5.3 CONCOMITANT AND REQUIRED INTERVENTIONS

Ideally, all infants enrolled in HEAL will be treated as consistently as possible across sites, so the major difference between subjects is their treatment with Epo vs. placebo. To this end, we have developed a set of recommended Clinical Guidelines regarding the treatment of infants with HIE, and sites will be encouraged to follow these guidelines when possible. These guidelines address 1) hypothermia therapy duration and goal temperature, as well as use of sedatives; 2) monitoring of laboratory tests during the neonatal period; 3) EEG brain monitoring; and 4) general approach to treatment of clinical and sub-clinical seizures.

5.4 PROHIBITED AND PRECAUTIONARY INTERVENTIONS

There are no prohibited and precautionary interventions for this study.

5.5 ADHERENCE ASSESSMENT

Study drug will be administered by a nurse (i.e., bedside nurse, charge nurse, etc.) in the neonatal intensive care unit. A detailed record of study drug administration will be kept by Investigational Drug Services Pharmacy, and documentation of study drug administration will be completed by the nurse administering the drug.

6 CLINICAL AND LABORATORY EVALUATIONS

6.1 SCHEDULE OF EVALUATIONS

Table 3. Schedule of Evaluations

Evaluation	Visit Day	Window	Location	Details
Screening	Study Day 0 or 1	Before randomization	NICU	Screening Clinical Assessment (Apgar, resuscitation, acidosis, Modified Sarnat exam, Inclusion/Exclusion assessment)
Therapeutic Hypothermia Begins	Study Day 0 or 1	Begin by <6 hours post-birth	NICU	Begins prior to administration of study drug and at <6 hours of age, continues for 72 hours, per standard of care.
Consent & Enrollment	Study Day 0 or 1	Prior to 24 + 2 hours post-birth	NICU	Informed Consent
Randomization	Study Day 0 or 1	Prior to 24 + 2 hours post-birth	Pharmacy	HEAL Portal
Blood sample (pre-intervention)	Study Day 0 or 1	Before Intervention 1	NICU	1.5 mL blood sample
Urine sample 1	Study Day 0 or 1	1st void after consent	NICU	1-2 mL urine sample, ideally obtained prior to study drug administration (note: urine may be collected after study drug administration if not available earlier, and in this case, does not require a Protocol Deviation)
Intervention 1 (study drug)*	Study Day 1	Prior to 24 + 2 hours post-birth	NICU	Target time is as soon as possible
Intervention 2 (study drug)	Study Day 2	24 ± 2 hours following Intervention 1	NICU	Target time is same time of day as Intervention 1
Blood sample 2	Study Day 2	24 ± 2 hours following Intervention 1	NICU	1.5 mL blood sample. Target time is same time of day as Intervention 2
Intervention 3 (study drug)	Study Day 3	48 ± 2 hours following Intervention 1	NICU	Target time is same time of day as Intervention 1
Urine sample 2	Study Day 3-4	After rewarming completed, and within 24 hours after rewarming	NICU	1-2 mL urine sample

Evaluation	Visit Day	Window	Location	Details
Intervention 4 (study drug)	Study Day 4	72 ± 2 hours following Intervention 1	NICU	Target time is same time of day as Intervention 1
Blood sample 3	Study Day 4	72 ± 2 hours following Intervention 1	NICU	1.5 mL blood sample. Target time is same time of day as Intervention 4
Brain MRI/MRS (routine clinical care)	Study Day 4-5	Ideally between 96-144 hours post-birth	NICU	After rewarming, and ideally after Intervention 4 has been administered
Clinical assessment	Study Day 5	Anytime this calendar day	NICU	Modified Sarnat exam
Intervention 5 (study drug)	Study Day 7	144 ± 2 hours following Intervention 1	NICU	Target time is same time of day as Intervention 1
EEG Summary	Study Days 1-7	N/A	NICU	Upload EEG reports
Clinical Blood Test Summary	Study Days 1-7	N/A	NICU	Record renal function, CBC, liver function labs, as collected per site's clinical standard of care.
Follow-up (month 4)	Month 4	Day of month (±7 days)	Phone	Contact info, intervening medical and developmental history, assess adverse events
Follow-up (month 8)	Month 8	Day of month (±7 days)	Phone	Contact info, intervening medical and developmental history, assess adverse events
Follow-up (month 12)	Month 12	Day of month (±28 days)	Phone	Contact info, intervening medical and developmental history, WIDEA, assess adverse events
Follow-up (month 18)	Month 18	Day of month (±28 days)	Phone	Contact info, intervening medical and developmental history, WIDEA, assess adverse events
Follow-up (month 24)	Month 24	Day of month (±56 days)	Phone (may also be done in clinic)	Contact info, intervening medical and developmental history, WIDEA, assess adverse events
Follow-up (month 24)	Month 24	Day of month (±56 days)	Clinic	Standard Neurologic Exam (Kuban), Bayley III, GMFCS Evaluation, Child Behavior Checklist (CBCL), growth parameters, assess adverse events

Evaluation	Visit Day	Window	Location	Details
Extended Contact Calls	Months 30, 36, 42, 48, 54, 60, 66, 72, 78, 84, 90, 96	Day of month (±56 days)	Phone	Confirm contact information, collect basic information about how child is doing. *Note: failure to complete, or completing these Extended Contact calls outside of window does NOT constitute a study deviation.*
* Study Day 1 is defined as the calendar day on which Intervention 1 is administered. If the study drug is administered on the calendar day following the day of birth, then the day of birth will be called Study Day 0.				

6.2 TIMING OF EVALUATIONS

6.2.1 Pre-Randomization Evaluations – Timing

Screening <24+2 hours post-birth: Screening and pre-entry clinical assessment may occur concurrently. Screening does not involve procedures that are not part of routine patient care. Patients should be screened and evaluated as soon as possible after birth. The Sarnat examination should be performed and recorded between 1-6 hours of age, preferably prior to administration of any sedative drugs. When performed by an outside physician or an individual not trained in the HEAL Sarnat exam, the Sarnat exam should be confirmed by a HEAL study physician (see Section 6.3.2.1).

Consent and Randomization (<24 hours): Consent must occur by 24+2 hours of age (i.e., post-birth). Study entry (i.e. randomization) may occur at any time up to 24+2 hours of age, as long as the first dose of study drug is administered by 24+2 hours (ideally ≤ 24 hours) of age.

Clinical Assessment (<24 hours): For subjects successfully screened, consented, and randomized, a baseline blood sample will be collected prior to giving the first dose of study drug. The 1.5 mL blood sample must be collected within the first 24+2 hours of age, which is the latest time point at which drug administration can be begin. A 1-2 mL urine sample will be collected from the first void after consent is obtained. The urine does not need to be collected prior to giving the first dose of study drug. When possible, EEG monitoring will also begin by 24+2 hours of age, as will laboratory monitoring according to the HEAL Clinical Guidelines and institutional guidelines for care of critically ill newborns. We will request placental pathology reports, as available. To facilitate obtaining this data, clinical pathologic examination of the placenta should be performed when possible.

6.2.2 Birth Hospitalization Evaluations – Timing

Blood and urine samples. During the same four-hour window permitted for delivery of study drug on Study Day 2, a 1.5 mL blood sample will be collected for biomarker analyses (Section 6.3.5). A final blood (1.5 mL) sample for biomarker studies will be performed during the same four-hour window permitted for delivery of study drug on Study Day 4. A urine sample (1-2 mL) will also be collected after rewarming, on Study Day 3-4.

EEG or aEEG. EEG or aEEG will be performed during the first 3 days, consistent with HEAL Clinical Guidelines whenever possible. Continuous EEG monitoring is encouraged, as recommended by the American Clinical Neurophysiology Society in their 2011 published guidelines for care of neonates with HIE [123].

MRI/MRS. The patient will undergo a clinical head MRI/MRS, ideally 96-144 hours post-birth per clinical standard of care. If a subject is transitioning to comfort care and has not received any head imaging (head ultrasound (HUS) or MRI/MRS), it is recommended to obtain a HUS if possible.

Modified Sarnat. A modified clinical Sarnat exam will be repeated, ideally by a physician who has been trained on the HEAL Sarnat exam, when possible, and recorded on Study Day 5.

6.2.3 Follow-Up Evaluations – Timing

The following evaluations will be performed on all surviving subjects who are consented and randomized, regardless of whether they completed the study intervention. Note that subjects who are discharged from the hospital prior to completing the study intervention will still be followed, with all efforts made to retain such subjects in the study for purposes of collecting outcome data.

Interim phone evaluations. Follow-up phone contact will be carried out at 4 months ± 7 days, 8 months ± 7 days, 12 months ± 28 days, 18 months ± 28 days of age, and 24 months ± 56 days of age. The 24-month phone call may be performed in the clinic concurrent with the 24-month visit. At each phone interview, contact information will be confirmed and updated as appropriate, and information about intervening medical and developmental history will be sought. The WIDEA parental questionnaire will also be administered at 12, 18, and 24 months of age.

6.2.4 Final Endpoint Evaluation

24-month neurodevelopmental examination. Formalized testing (Standardized neurologic examination, Bayley III, and GMFCS) will be done in person at 24 months ± 56 days of age (i.e., ± 2 months).

6.2.5 Future Studies

We plan to apply for further funding that will allow us to continue to follow study subjects through age 8.

6.3 INSTRUCTIONS AND DEFINITIONS OF EVALUATIONS

6.3.1 Informed Consent

All newborn infants admitted to the intensive care nurseries at participating sites will be screened for eligibility by the study physicians and/or study coordinators on a daily basis. A study investigator or research coordinator (available by pager or cell phone) will be contacted regarding potential candidates. The parents or legal guardians will first be notified of the study by the treating physician. If they indicate an interest in learning more, then a study investigator or coordinator will approach the parents, describe the study, answer questions, and provide a study brochure and consent form. Infants must be consented, randomized, and receive study drug by 24 + 2 hours of age. In the event that an out-born infant does not have a parent present, we will attempt to obtain written consent over the phone when permitted. When an infant with HIE at an outside hospital is identified for transport to the study hospital, a study investigator and/or coordinator will be notified immediately of the impending transfer, so that necessary steps can be taken to achieve enrollment in the required timeframe. When possible, both parents will review, discuss, and sign the consent form with a study investigator and/or coordinator. At least one parent with legal authority to do so will be asked to sign the consent form and HIPAA form. In some cases, the form may be faxed, scanned and emailed, or texted as a photo to the investigator and/or coordinator. The signed Consent form will need to be provided per the enrolling hospital's requirements. A copy of the signed consent and HIPAA forms will be given to the family. A copy of the signed consent form will be placed in the medical record, as well as kept in the study records. The original signed consent form will be kept by the local site PI. HIPAA language may be included in the consent form as directed by local authorities.

6.3.2 Clinical assessment

Each of the clinical characteristics listed below will be recorded on the Case Report Forms (CRFs) at the time points indicated.

6.3.2.1 Screening clinical assessment (<24+2 hours):

1. Apgar scores at 5 and 10 minutes

2. Resuscitation: presence of endotracheal ventilation, mask ventilation, CPAP, or chest compressions at 10 minutes after delivery.

3. Acidosis: pH and base deficit in a cord, arterial, or venous blood sample taken before 60 minutes of age.

4. Sarnat Exam Procedures. The modified Sarnat Exam should be performed between 1-6 hours of age prior to administering sedative medications, by a neonatologist, neurologist, pediatrician or neonatal nurse practitioner. The qualifying Sarnat exam should be the worst exam before sedative medications are administered to the infant. The exam cannot be evaluated in an infant who is under the influence of paralytic medications.

A Sarnat Screening Worksheet (1 page, hard copy) must be filled out for all enrolled infants. We will record the age of the infant (in hours) at the time of the qualifying exam.

The examiner should be trained in the HEAL Sarnat examination whenever possible. If the examiner has not undergone HEAL Sarnat training, the exam should be *confirmed* by a HEAL physician by 24 hours of age, by at least one of the following mechanisms: 1) The HEAL research physician performed the qualifying exam; 2) HEAL research physician discussed the findings with the outside physician/transport team member who performed the exam; or 3) HEAL research physician corroborated the exam findings with a treating physician who spoke with the Sarnat examiner.

The Sarnat Screening Sheet must be **signed by the HEAL Physician** who confirmed the exam. It is permissible for signature to occur after the subject has been enrolled, however, whenever possible, the Sarnat should be verbally confirmed prior to enrollment.

If the qualifying Sarnat exam is not done prior to receiving sedating medications, and/or cannot be confirmed by a HEAL physician by 24+2 hours of age, the infant could still qualify for the study. However, this will be noted on the Sarnat Screening Sheet. If a HEAL physician determines that an enrolled infant did not actually meet Sarnat criteria for eligibility, and the infant has already been enrolled, then this should be reported as a protocol violation.

All items on the Sarnat Screening Sheet must also be entered into the HEAL Portal in order for the infant to be randomized and enrolled into the study.

The Sarnat Screening Sheet must be kept on file at each enrolling site, along with the infant's signed consent form. The Sarnat Screening Sheet will be used as a source document during monitoring visits, to ensure accuracy of data entry into the HEAL Portal.

5. Sarnat Exam Findings (**Table 4**).[3,5,26] The HEAL study will incorporate a Modified Sarnat Exam to determine the degree of encephalopathy. “Severe” encephalopathy refers to the presence of more symptoms classified in the severe category than in the moderate category. If signs are equally distributed between the two categories, then designation of severity is based on level of consciousness.[3] Degree of encephalopathy will be calculated within the HEAL Portal, based on the exam findings that are entered into the screening form.

Table 4. Modified Sarnat Encephalopathy[3,26]

Category	Normal	Mild Abnormality	Moderate Abnormality	Severe Abnormality	Unable to assess?
1. Level of consciousness	Normal	Hyperalert or irritable	Lethargic or poorly responsive	Minimal or no responsiveness	N/A
2. Spontaneous activity	Normal	Slightly decreased	Decreased	Absent	N/A
3. Posture	Normal	Mild distal flexion	Distal flexion, complete extension	Decerebrate	N/A
4. Tone	Normal	Hypertonic	Hypotonic	Flaccid	N/A
5. Primitive reflexes<br>a. Suck	Normal	N/A	Weak or bite	Absent	Unable to assess
b. Moro	Normal	Low threshold to elicit	Weak or incomplete	Absent	Unable to assess
6. Autonomic<br>a. Pupils	Normal	N/A	Constricted	Dilated and either fixed or sluggishly reactive; asymmetric	Unable to assess
b. Respiration	Normal	N/A	Periodic Breathing	Intubated and ventilated	N/A

6.3.2.2 Demographics

Demographic information: maternal and paternal race and ethnicity; maternal education will be collected prior to discharge. Variables from the NINDS Common Data Elements will be used.

6.3.2.3 Day five clinical assessment

Modified Sarnat score (**Table 4**). At Study Day 5, the infant will undergo another Sarnat examination. Unlike the initial Sarnat exam, the Sarnat scoring sheet does not need to be retained for this follow-up exam. Exam findings will be entered into the HEAL Portal. The exam should be conducted (or confirmed) by a Physician who has been trained on the HEAL Sarnat exam, when possible.

6.3.2.4 Newborn hearing screening

If performed, results from the newborn hearing screening will be recorded.

6.3.3 EEG assessments

Brain monitoring via conventional video-EEG should start as soon as possible after NICU admission (see Clinical Guidelines). For sites where video-EEG is not available 24/7, amplitude-integrated EEG (aEEG) monitoring should be started as soon as possible. If patient develops suspected clinical or aEEG seizures, conventional video-EEG should be initiated and continued until the patient has been free from all seizures for 24 hours. Sites that perform continuous EEG (cEEG) throughout hypothermia should save de-identified cEEG data for analysis in future ancillary studies.

6.3.3.1 EEG interpretation

EEG interpretation will be based on the clinical EEG report.

The following data will be recorded on the CRFs:

- Electrographic seizures or status epilepticus
- Clinical seizures

6.3.4 Laboratory Evaluations

Standard of care for infants with HIE includes intermittent monitoring for glucose instability, renal and hepatic injury. Other labs such as complete blood counts, thyroid function, and cardiac function may also be obtained if clinically indicated. We will record a subset of these results to determine the presence or absence of common complications of HIE. HEAL guidelines have recommended time windows for these laboratory studies, and these timings are consistent with existing institutional protocols at HEAL enrolling sites.

6.3.5 Plasma and urine biomarkers; baseline Epo measurement

Three blood samples (1.5 mL each) will be collected in EDTA tubes from each enrolled patient over the course of their hospital stay to be used to study circulating biomarkers of inflammation and brain injury. The first blood sample (collected prior to the administration of study drug) will also be used to measure a baseline Epo level. A baseline Epo level may be helpful to determine the chronicity of hypoxic-ischemic stress *in utero*, a potential confounder of the relationship between Epo therapy and 2-year outcome. Additional samples are collected on Study Days 2 and 4.

Samples may be refrigerated for up to 4 hours prior to processing and freezing. As soon as possible after drawing, samples should be spun for 8 min at 2000 G, plasma removed into a separate container, and plasma and pellet stored in labeled tubes at -70°C or below. RNALater will be added to one pellet to preserve RNA for future studies. Please refer to the Laboratory SOP for further details. Plasma Epo concentrations will be measured in duplicate using Meso Scale Discovery (MSD) technology at the UW Laboratory Core.

We will assay blood samples for circulating biomarkers of brain injury and inflammation in a subset of infants (N = 200; 100 treated and 100 control infants with balanced numbers of moderate and severe HIE. These subjects will all have MRI scans that have met pre-defined acceptance criteria). We will assay putative biomarkers of brain injury severity in neonates including: S100B, glial fibrillary acidic protein (GFAP), Tau, neuron-specific enolase (NSE), and ubiquitin C-terminal hydrolase-L1 (UCH-L1). Given the role of inflammation in HIE, we will also measure the following inflammatory markers: interleukin (IL)-1beta (β), IL-6, IL-8, and tumor necrosis factor-alpha (TNF-α). All samples will be run in duplicate using MSD technology in the UW core laboratory. This combination of factors will allow us to evaluate the subject's inflammatory

state over time, provide insight as to the timing and severity of brain injury, and potentially, provide insight as to why some individuals might respond more optimally to Epo.[124-127]

Residual plasma and cell pellets will be banked (with parental consent) and used for future mechanistic studies such as epigenetic or microRNA associations with certain outcomes. Two urine samples (1-2 mL each) will be banked for future studies to better understand consequences of HIE and/or Epo therapy (separate funding).

6.3.6 Neuroimaging: Brain MRI/MRS

At each of the participating sites, patients will be imaged on a 3T MRI, ideally between 96-144 hours post-birth. This timing is consistent with the American Academy of Neurology practice parameter which suggested imaging before 8 days of age.[128] In cooled infants, the MRI/MRS is most sensitive and specific if performed at < 8 days of age.[117] Since therapeutic hypothermia ends at 4 days of age (study day 3-4), we anticipate that brain imaging can be performed after completion of therapeutic hypothermia and between 96-144 hours post-birth in study patients, as part of routine clinical care. All study sites have agreed to follow a specific study protocol including collection of high quality MR spectroscopy data (see below); to demonstrate excellent signal to noise ratio; and to have a MRI physicist and/or neuroradiologist oversee the study procedures. Infants may receive more than one brain MRI, and some may also receive a spine MRI. All brain and spine MRI studies will be anonymized and transmitted electronically in a secure fashion to the Neuroimaging Core.

Any patients discharged prior to 96-144 hours post-birth may have their MRI performed prior to discharge, even if this timing is outside of the pre-specified study window. Similarly, if a subject is unable to have an MRI done during the desired window due to clinical instability such as being on ECMO, the MRI should be done as soon as is clinically appropriate. Out-of-window MRIs will not be considered protocol deviations.

All sites will perform the following set of sequences: A) 3D T1-weighted (isotropic 1x1x1 acquisition); B) Axial T2 weighted (1x1x2 with TE >=120); C) Diffusion tensor imaging (DTI) (25-32 directions with b value of 1000 s/mm2; isotropic 2x2x2); and D) single voxel PRESS magnetic resonance spectroscopy (MRS), TE 35ms, with 1 voxel in the left thalamus/basal ganglia, and 1 voxel in the left parietal white matter. In addition, one long TE spectrum (TE=144 ms) will be acquired from the identical left thalamus/basal ganglia ROI. The Neuroimaging Core will work with sites to ensure quality and consistency of MRI and MRS data optimized for each model of MRI, will perform centralized interpretation of these neonatal brain scans, and will process the MR spectroscopy data and provide masked interpretation of these data.

6.3.7 Phone questionnaires

Interim data collection will occur via telephone contact at 4, 8, 12, 18, and 24 months of age. The phone call interviews may also be done in person if the patient is being seen in the clinic during any of these times. During these follow-ups, an interval medical and developmental history questionnaire will be administered. At 12, 18, and 24 months, the WIDEA, a standardized parental questionnaire regarding infant development will be included in the assessment.

6.3.8 Neurodevelopmental examinations (24 months)

At 24 months ± 56 days (i.e., ± 2 months), we will perform the following in-person evaluations to determine the primary and secondary outcomes. This section provides a brief description of each of these evaluations. For a description of the primary outcome itself, see Section 9.2.1.

To ensure that complete primary endpoint data can be obtained in as many subjects as possible, it is strongly recommended that the 24-month neurodevelopmental examinations be performed in the following order:

1. Bayley III exam
   a. Cognitive
   b. Language
   c. Motor
2. Standardized neurologic exam
3. GMFCS

The follow-up examination may be performed by the following individuals, provided they have received the appropriate training through HEAL. The preferred order is as follows:

- Pediatric neurologist
- Developmental pediatrician
- Other pediatrician involved in infant follow-up
- PNP involved in infant follow-up
- Pediatric physical therapist (who is trained in the HEAL standardized assessments)

Primary outcome measurement:

a) Standardized neurologic examination, based on the examination used in the ELGAN study protocol[129,130]

b) GMFCS[131]

c) Bayley III cognitive score[132]

Secondary outcomes:

a) Developmental history

b) Medical history including seizure history

c) Bayley III cognitive and language score

d) CBCL externalizing score

6.3.8.1 Standardized neurologic examination.

The presence and type of CP will be determined using the broadly accepted and standardized examination described and used in the ELGAN study (the examination will be housed on our HEAL portal).[129] This software and training program developed by our co-investigator Dr. Karl Kuban provides a method of neurologic testing of subjects in a formalized, systemized method that is highly reproducible. This examination is designed specifically to determine the presence and classification of CP in a 2-year old child. The follow-up examiners at each study site will undergo in-person training and certification. To standardize the quality of data regarding neurological exams, examiners will attend a half-day workshop at the annual PAS meeting or at a central location (e.g., Minnesota or Missouri) or virtual location, view a training video, and then classify neurological findings illustrated on an assessment video.[133] Inter-observer variability assessments will be done to determine agreement with gold standard responses. Annotated feedback will be given to examiners regarding items

that had a less than 85% correct rate, and, based on experience in the ELGAN and PENUT studies, we expect agreement rate to rise to over 90%.[133,134] Training will entail participation in a training session with Dr. Kuban, followed by the review of a training video, and submission of a set of answers regarding scoring of the neurologic exam that will be used to certify the examiner.[130] A re-certification process will be performed every 18 months to ensure that primary outcome examiners remain adequately trained throughout the duration of the study. The neurologic examination will produce one of the following neurologic diagnoses: no CP, diparetic CP, hemiparetic CP, or quadriparetic CP.[135] These categorizations correlate highly with long-term neurodevelopmental outcomes.

6.3.8.2 Gross Motor Function Classification Scale (GMFCS)[131]

This classification scale focuses on children's functional achievements rather than on their limitations. It places emphasis on the child's routine performance (not necessarily their best capacity) in the home or community setting. The GMFCS system defines 5 levels of function that represent an ordinal scale where the distance between levels is not considered equal. A brief summary of the GMFCS score at age 24 months is as follows:

Descriptions of each level for ages 12 to 24 months

Level 0 = Walks ten steps independently and has symmetric gait.

Level 0.5 = Walks ten steps independently but does not have symmetric gait.

Level 1 = Infants move in and out of sitting and floor sit with both hands free to manipulate objects. Infants creep or crawl on hands and knees, pull to stand and take steps holding onto furniture. Some may creep or bottom shuffle, but are able to "travel" independently. Infants walk between 18 months and 2 years of age without holding on.

Level 2 = Infants maintain floor sitting but may need to use their hands for support to maintain balance. Infants creep on their stomach or crawl on hands and knees. Infants may pull to stand and take steps holding onto furniture.

Level 3 = Infants maintain floor sitting when the low back is supported. Infants roll and creep forward on their stomachs.

Level 4 = Infants have head control but trunk support is required for floor sitting. Infants can roll to supine and may roll to prone.

Level 5 = Physical impairments limit voluntary control of movements. Infants are unable to maintain antigravity head and trunk postures in prone and sitting. Infants require adult assistance to roll.

(Modified from Palisano et al, Med Child Neurol, 1997; 39:214-233. Algorithm adapted by Rosenbaum P and Saigal S for TIPP Trial, and utilized in the NICHD Neonatal Research Network under the direction of Betty Vohr).

To determine the level of gross motor function of the infant, follow the algorithm shown on the Gross Motor Function Work Sheet. The algorithm starts with normal function and progresses to increasing levels of functional limitations.

6.3.8.3 Bayley Scales of Infant Development III (Bayley-III)

The Bayley III is the most widely used developmental measure to determine developmental delay in high-risk infants.[132,136] Since 2005, the Bayley III has been adopted for standard use for the vast majority of follow up programs as well as neonatal clinical trials including the hypothermia trials.[3-5,7] We will measure Bayley III cognitive, motor, and language scores. Only the cognitive score will be part of the primary outcome measure, and as such, this portion of the Bayley III exam should be performed first. We will not use the social-emotional and adaptive behavioral subtests of the Bayley III. All individuals who will perform the Bayley exam will undergo a certification process to ensure reliability of test results. This process will include review of a "gold standard" Bayley III assessment performed by a neuropsychologist, and sending in a videotape of the HEAL examiner performing a Bayley evaluation of a 2-year-old along with the completed test booklet. Dr. Lowe will evaluate this tape and the associated test booklet for completeness and correctness. Only individuals certified by Dr. Lowe on behalf of HEAL will administer the test.

6.3.8.4 Developmental history

We will interview the parents regarding the age of developmental milestones including rolling over, sitting, standing, babbling, speaking, and performing pincer grasp.

6.3.8.5 Medical and social history

General information will be gathered from families, including:

a) Primary language spoken at home
b) Maternal (or primary caregiver) education

We will interview parents at pre-specified time points regarding the following items:

c) Presence of afebrile seizures
d) Use of supplemental oxygen, mechanical ventilation, or tracheostomy
e) Use of gastrostomy or nasogastric tube feedings; ability to self-feed; ability to eat solids
f) Hospitalizations
g) Utilization of rehabilitation services (OT, PT, or ST)
h) Maternal (or primary caregiver) employment
i) Vision or auditory issues
j) Medications, including seizure medications

6.3.8.6 Growth parameters

At 2 years, we will measure head circumference, length, and weight.

6.3.8.7 Child Behavior Checklist (CBCL)

At 2 years, we will administer the CBCL, a parental questionnaire that includes 99 items that describe specific kinds of behavioral, emotional, and social problems. We

will specifically evaluate the externalizing score for evidence of attention problems and aggressive behavior. This checklist will be scored centrally.

6.3.8.8 Warner Initial Developmental Evaluation of Adaptive and Functional Skills (WIDEA)
The WIDEA is a questionnaire developed and standardized to assess the 4 functional domains of self-care, motor function, communication, and social cognition in young children. Normative data are available for this instrument through 36 months of age. Dr. Elizabeth Rogers (follow-up PI) will provide an interactive WIDEA video training session and certification for study personnel who will administer this parental questionnaire.[121,122]

6.3.9 Extended Contact Phone Calls

At the 24-month study visit, or by phone after the visit has been completed, the parent/guardian should be approached for consent for participating in Extended Contact phone calls. If consent is obtained, these calls should be performed at the following intervals:

- 30 months (±56 days)
- 36 months (±56 days)
- 42 months (±56 days)
- 48 months (±56 days)
- 54 months (±56 days)
- 60 months (±56 days)
- 66 months (±56 days)
- 72 months (±56 days)
- 78 months (±56 days)
- 84 months (±56 days)
- 90 months (±56 days)
- 96 months (±56 days)

These phone calls will be used to maintain relationships with participating families, ensure updated contact information is on file, and to gather basic information about how the child is doing. Data may be used to support future applications for follow-up studies on this population.

Failure to complete these follow-up calls, or performing these follow-up calls outside of the requested windows, does not constitute a study deviation.

Any deaths identified during these Extended Contact phone calls must be reported as SAEs.

7 MANAGEMENT OF SERIOUS ADVERSE EVENTS AND COMPLICATIONS OF HIE

The study will be monitored by a **NINDS-appointed DSMB** with independent experts in the areas of pediatric neurology, neonatology and biostatistics. The study will be monitored for safety by the DSMB with reviews for enrollment, follow-up, data quality, overall study conduct, participant safety, and significant adverse event rates. Meeting frequency and conduct will be detailed separately by the DSMB. For this NINDS grant, the DSMB will follow the guidance presented in the NINDS Guidelines for Data and

Safety Monitoring:
http://www.ninds.nih.gov/research/clinical_research/policies/data_safety_monitoring.htm

A neonatologist who is independent of the study will serve as the **Medical Monitor**, and will monitor all serious adverse events concurrently. The Medical Monitor will be masked to treatment allocation.

7.1 SERIOUS ADVERSE EVENT DEFINITIONS

In the HEAL study, the following events, occurring within 30 days of study drug dosing, will be reported as Serious Adverse Events (SAEs). Death occurring any time during the neonatal or follow-up period will always be reported as an SAE.

- **Systemic hypertension**
    - Definition: If blood pressure is elevated enough to require antihypertensive therapy
- **Polycythemia**
    - Definition: Central hematocrit (Hct) > 65.0%, as measured by a central lab on 2 consecutive free-flowing arterial or venous samples.
      (Note: High Hct values measured by point of care tests are not sufficient for meeting polycythemia criteria for purposes of this study)
- **Disseminated intravascular coagulation (DIC)**
    - Clinical bleeding or oozing requiring transfusion of blood products (e.g., FFP, cryoprecipitate or platelets)
      (Note: Laboratory findings may include elevated INR, PTT, PT, or D-dimers, or low PLT or fibrinogen)
- **Major venous or arterial thrombosis (clot): Definition = any of the following 3:**
    - Any thrombosis that is treated with a course of anticoagulation
    - Any venous or arterial thrombosis involving a major vessel **not** related to a central line
    - Any **symptomatic** thrombosis involving a major vessel (e.g., symptoms such as superior vena cava syndrome)
- **Pulmonary hypertension**
    - Elevated pulmonary vascular resistance treated with inhaled nitric oxide or ECMO therapy
- **Intracranial hemorrhage**
    - Intraparenchymal or intraventricular blood visualized by HUS or MRI T1 or T2 sequences
- **Cardiopulmonary arrest**
    - Clinical code event requiring chest compressions or epinephrine bolus from which the infant recovers (does not lead to death), that is not secondary to endotracheal tube (ETT) obstruction or other mechanical issue
- **Other unexpected life-threatening event**
    - Unexpected for HIE, or unexpected based on the Epo drug profile
- **Death**

The definition for an SAE in our population of HIE newborns is consistent with 21 CFR 312.21 (2005).

7.2 SAE RELATEDNESS & SEVERITY

For all Serious Adverse Events (SAE, as noted above), the site investigator will use their best medical judgment and indicate whether the SAE is related to the research (i.e., related to any research procedure including but not limited to the main intervention). The investigator will assign the SAE to one of the four categories below:

- **Definitely related:** The SAE ***is clearly related*** to the intervention
- **Probably related:** The SAE ***is likely related*** to the intervention
- **Possibly related:** The SAE ***may be related*** to the intervention
- **Unlikely:** The SAE ***is doubtfully related*** to the intervention
- **Not related:** The SAE *is clearly* ***NOT*** *related* to the intervention

The investigator will also provide an SAE severity:

- **Mild:** asymptomatic or mild symptoms; clinical or diagnostic observations only; no intervention indicated
- **Moderate:** minimal, local, or noninvasive intervention indicated
- **Severe:** Severe or medically significant, but not immediately life-threatening; hospitalization or prolongation of hospitalization indicated; disabling
- **Life-threatening:** Life-threatening consequences; urgent intervention indicated

7.3 SAE AND UNEXPECTED, RELATED EVENT REPORTING

All SAEs or events that are unexpected and thought to be related to study drug will require expedited reporting to the CCC. These events must be reported to the CCC PIs (Wu/Juul) within 72 hours of the site becoming aware of the event. Full documentation of the event is required within 7 days of becoming aware of the event. For in-hospital SAEs occurring during the neonatal period, it is expected that the site will be made immediately aware of the event. During follow-up, patients will be followed by phone (at 4, 8, 12, and 18 months of age) and in person (at 24 months of age). During these follow-up encounters, a questionnaire will be administered to cover medical complications following discharge from the neonatal intensive care unit. All deaths, SAEs occurring within 30 days of study drug dosing, and events unexpected and thought to be related to study drug occurring after hospital discharge must also be reported to the CCC PIs within 72 hours of the site becoming aware of the event.

Following notification from the site, the CCC will ensure reporting per Table 5 and Figure 2 below.

Table 5. SAE and Unanticipated Related Drug Effects Reporting Timelines

Group responsible for doing notification	Group Notified	Required Notification Timelines for: SAEs	Required Notification Timelines for: Unanticipated Related Drug Effects
Site	CCC PIs	▪ Notification within 72 hours of site becoming aware of event; SAE CRF completed by 7 days*	▪ Notification within 72 hours of site becoming aware of event; SAE CRF completed by 7 days*
Site	Site IRB	▪ Per site IRB requirements	▪ Per site IRB requirements
CCC (Wu/Juul)	Medical Monitor	▪ Provide SAE report within 7 days of site becoming aware of event	▪ Provide SAE report within 7 days of site becoming aware of event
CCC (Wu)	FDA	▪ Death or life-threatening SAES that are **unexpected and Epo-related**: no later than 7 calendar days of sponsor's (CCC) initial receipt of the information<br>▪ Other SAEs that are **unexpected and Epo-related**: as soon as possible, but no later than 15 calendar days of sponsor (CCC) determining the event qualifies for reporting	▪ N/A, unless serious (see SAE column)
Medical Monitor	DSMB	▪ Epo-related SAEs: within 15 calendar days after sponsor's initial receipt of the information	▪ Within 15 calendar days after sponsor's initial receipt of the information

**In some instances, the CCC and/or Medical Monitor may require more expeditious completion of the SAE form*

Figure 2. SAE and Unanticipated Related Drug Effects Reporting Timelines

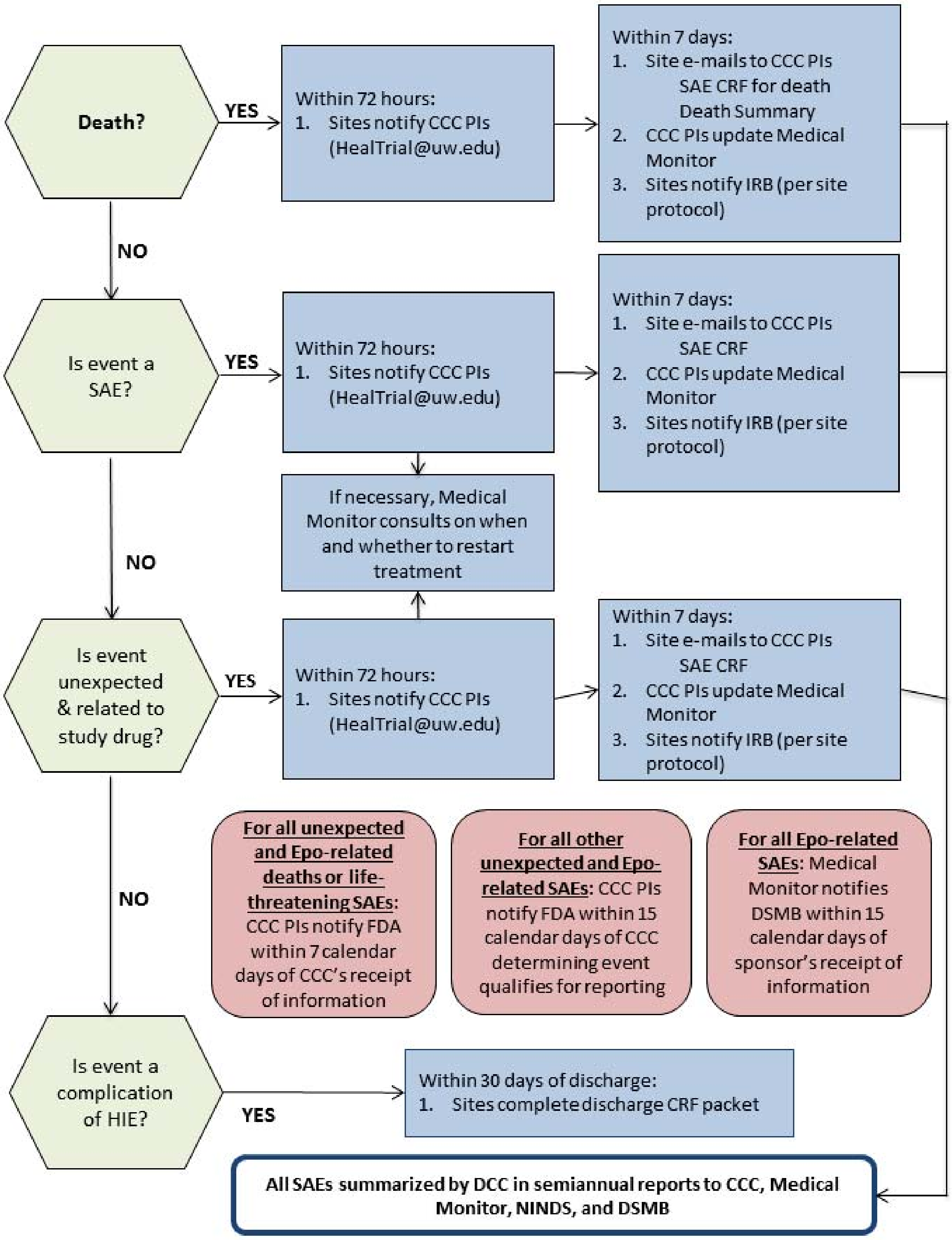

7.4 ANTICIPATED MEDICAL COMPLICATIONS (ADVERSE EVENTS) IN HIE PATIENTS

Critically ill infants with HIE commonly experience multiple medical complications. Medical complications of HIE patients will be recorded on case report forms, entered into the portal, and analyzed to determine if they occur in different frequencies between the treatment and control groups.

7.5 CRITERIA FOR MODIFICATION OF INTERVENTION REGIMEN

7.5.1 Criteria for Withholding/Stopping the Study Drug

- Central HCT > 65.0%
- Systemic hypertension *requiring treatment* as determined by the clinical team.[137,138]

7.5.2 Restarting Study Drug

- Study drug will be restarted when central HCT < 60.0%
- Study drug will be restarted when blood pressure is within the normal range for age for 48 hours, or normotensive on a stable dose of anti-hypertensives.
    - If the study drug is held on Study Days 1-4, <u>one</u> held dose may be made up, as long as it is given >20 hours before and >20 hours after any other study drug doses, and before the Study Day 7 dose window.
    - If the study drug is held on Study Day 7, the held dose may be made up any time though Study Day 10.
    - Only <u>one</u> held dose may be made up, in total.
- Note that any per-protocol blood samples should be drawn at the protocol-specified times. Blood sample times are <u>not</u> adjusted in cases where study drug is held.

<u>Open Label Use of Epo.</u> Open label use of Epo is not permitted in enrolled infants during the initial neonatal hospitalization. It is very unlikely that open label Epo use will pose a problem, since standard treatment for acute anemia in term infants is red blood cell transfusion, and Epo is rarely (if ever) used in this clinical setting.

If study drug is discontinued in any subject, parents will be encouraged to continue to allow their child to participate in the follow-up evaluations.

7.6 PROTOCOL VIOLATION/ DEVIATION REPORTING

Protocol violations require completion of a Protocol Violation Form and notification to the CCC and DCC within 3 working days. Protocol deviations require completion of a Protocol Deviation form, and the CCC and DCC should be notified within 7 days of occurrence.

7.6.1 Protocol Violations:

- Un-masking of study personnel
- Enrollment despite meeting exclusion criteria
- Enrollment despite not meeting inclusion criteria
- Consent not obtained in accordance with IRB guidelines
- Study drug administration or dosing error (incorrect study drug administered)
- Wrong study drug dose administered (>40 units, and >10% off from specified dose [1000 units/kg birthweight])
- Study drug administered when it should have been withheld

- Open label Epo administered
- 24-month clinic visit missed

7.6.2 Protocol Deviations may include the following items, among others:

- Hypothermia performed not in accordance with site's hypothermia protocol (Examples include equipment malfunction, or early discontinuation due to reclassification of a patient as mild severity (i.e., even if a site has an early exit protocol, this early exit should be reported as a protocol deviation). Reasons for early discontinuation consistent with a site's cooling protocol are not considered protocol deviations (e.g., stopping cooling for ECMO, etc.))
- First study drug dose given after 26 hours of life
- Study drug dose is given outside of prescribed time window
- Study drug dose not given
- Wrong study drug dose administered (>40 units, but ≤10% off from specified dose [1000 units/kg])
- Blood samples drawn outside of the accepted time windows
- Blood collection missed
- Blood sample not in accordance with requirements per SOP (processing error, shipping error, other)
- Urine collected outside of the accepted time windows
- Urine collection missed
- Urine sample not processed appropriately
- Lost urine or blood sample
- Day 5 Sarnat not done
- Day 5 Sarnat performed out of window
- Follow-up phone contact missed
- Follow-up performed out of window

8 CRITERIA OF INTERVENTION DISCONTINUATION

If the patient experiences any SAE that might be considered related to study drug, the site investigator should notify the HEAL CCC PI (Drs. Wu/Juul) immediately, and hold study drug until the event can be evaluated by the Medical Safety Monitor. The Medical Monitor will decide whether to continue therapy thereafter. If the remaining doses of study drug are held, all efforts will be made to maintain the subject's participation in follow-up activities at 4, 8, 12, 18, and 24 months of age. The site always maintains the ability to hold study drug or intervene on behalf of the subject for safety reasons.

9 STATISTICAL CONSIDERATIONS

9.1 GENERAL

This study is a randomized parallel group double-masked, placebo controlled trial in neonates with HIE. Eligible neonates will be enrolled and treated with either Epo or normal saline over 7 days and followed for a fixed period of 24 months (±56 days) for survival and neurological outcomes. The follow-up period was chosen to provide meaningful motor and cognitive outcomes, since CP and motor and cognitive

deficits are more reliably diagnosed at 2 years of age. Enrolled children will be randomized, stratified by study site and severity of HIE, with equal likelihood to receive Epo or saline.

Analyses will be based on a modified Intention to Treat (mITT) approach. In this approach, all randomized neonates who received at least one dose of study drug will be included in the analyses. Neonates whose parents withdrew consent after randomization and before the first dose will be excluded from the mITT. Since the first dose is within 24 hours of birth and within at most a few hours after consent and randomization, we do not expect more than a very small number of withdrawals.

Preliminary analyses will compare baseline characteristics to explore whether there are any imbalances that occurred at randomization. These analyses will include comparing demographics and the Sarnat score at study entry.

9.2 OUTCOMES

9.2.1 Primary outcome

Primary outcome is the composite of death or neurodevelopmental impairment.

Neurodevelopmental impairment (mild, moderate, or severe) is defined as any of the following:

- GMFCS level ≥1, or
- GMFCS = 0 or 0.5 AND CP (any type), or
- Bayley III Cognitive Score <90

Since death is a competing outcome, it is critical to include it in the primary outcome measure. We will use standardized, validated neurological and developmental assessments:

- CP diagnosed by Standardized Neurological Examination[113,114]
- Bayley III Cognitive Score
- GMFCS[131]

9.2.1.1 Motor Deficit – Cerebral Palsy

CP will be determined by a Standardized Neurologic Examination[129,130] conducted under the direction of Dr. Karl Kuban (Co-I), using the systematized exam and video-based certification system created for the NINDS-funded ELGAN and PENUT studies.[129,130] This training program provides a formal method of neurologic testing that is highly reproducible. The exam was created specifically to determine the presence and classification of CP (i.e., quadriplegic-QP, hemiplegic-HP and diplegic-DP) at age 2. To standardize the quality of neurologic examination findings, two follow-up examiners at each site will participate in a training session, review training videos, and submit a set of independently scored examinations that will be used for certification, as is being done currently in the PENUT trial.[130] A re-certification process will be performed at least every 18 months to ensure that primary outcome examiners remain adequately trained throughout the study. Inter-observer variability assessments will be done to determine agreement with gold standard responses. Annotated feedback will be given to examiners regarding items that had a < 85% correct rate. Based on experience in the ELGAN study, we expect agreement rates to exceed 90%.[130]

9.2.1.2 Motor Deficit: Gross Motor Functional Classification System (GMFCS)

The GMFCS is a well-accepted and validated tool that is used widely to classify motor functional outcomes. Distinctions between levels of motor function are based primarily on functional ability. The GMFCS was used to determine motor outcome in the NINDS-funded Beneficial Effects of Antenatal Magnesium Sulfate (BEAM) trial, as well as in 6 of 7 hypothermia trials for HIE. We will apply the BEAM trial algorithm and definitions when assigning GMFCS scores. Level 0 = normal gait; Level 0.5 =asymmetric gait; Level 1 = walks independently with abnormal gait or requires ankle-foot orthosis; Level 2 = cruises, pulls to stand, sits hands free; Level 3 = sits propped on hands only, rolls both ways; Level 4 = sits when supported in lower trunk, has head control, can roll to supine; Level 5 = no head or trunk control, no rolling, little or no voluntary movement. These definitions are modified from Palisano et al, Med Child Neurol, 1997; 39:214-233. Algorithm adapted by Rosenbaum P and Saigal S for TIPP Trial, and utilized in the NICHD Neonatal Research Network under the direction of Betty Vohr.

9.2.1.3 Cognitive Deficit: Bayley III Cognitive Score < 90

Bayley III Cognitive Score[112] is a standard test used to evaluate early cognitive outcomes in high-risk infants. Severity of cognitive deficit is defined by number of standard deviations (SD) below the mean (i.e., severe, moderate, and mild deficit = -3, -2 and -1 SDs below the mean). Since the Bayley III yields higher cognitive scores than the previous version of the Mental Development Index on the Bayley II,[136,139-141] we have defined cut-offs on the Bayley III that are 5-15 points shifted to the right compared to the Bayley II. We consider any Bayley III Cognitive Score < 90 as abnormal, with mild, moderate, and severe ranges as defined in Table 7. These definitions are consistent with findings in a contemporary cohort of infants with HIE who underwent hypothermia,[141] and also consistent with cut-offs used in the PENUT trial. Each site will undergo certification by reviewing a “gold standard” Bayley III assessment performed by our research neuropsychologist. Each site psychologist will then record a Bayley III assessment of a 2-year-old child, and submit the test booklet and video recording for review by the research psychologist for feedback. HEAL CCC leadership has experience in performing multicenter Bayley III certification in other NIH funded studies (e.g. PENUT, TOLSURF), and will use the infrastructure in place for these studies. Only Bayley III examiners who have undergone this training and certification will be allowed to perform the 2-year primary endpoint evaluation.

9.2.1.4 Adjudication of Primary Outcome

There may be cases of only a phone follow-up rather than an in-person visit, or otherwise partial data. The CCC will create an Outcomes Adjudication Committee, who will be masked to the treatment assignment of the child. The Committee will receive all available data on the long-term outcome of these toddlers and will assess whether they can definitively assign a primary outcome level and what it is, or they do not have sufficient information to make that determination and therefore the value will be imputed. This model was successfully previously used in the NO CLD clinical trial long-term outcome.[142]

9.2.2 Secondary outcomes

Overall severity will consist of the *worst* severity observed in either motor or cognitive outcomes. Severity of motor impairment will be determined by type of CP and GMFCS level (Table 6). Severity of cognitive impairment will be determined by Bayley III Cognitive Score (Table 7).

At age 2, we will assess the effect of Epo on secondary outcomes: a) presence of CP, b) severity of motor impairment, c) Bayley III cognitive and language scores, d) epilepsy (i.e., ≥ 2 afebrile, unprovoked seizures), and e) behavioral abnormalities (i.e., attention problems or aggressive behavior) based on the CBCL externalizing score.[143] To explore the effect of Epo on sensory deficits that can result from HIE, we will collect 2-year data regarding hearing impairment requiring hearing aids, and if available, information about the presence of cortical visual impairment diagnosed by an ophthalmologist. To elucidate the effect of Epo on all severities of impairment, we will analyze the effect of Epo on the following 4-level outcome: 1) normal, 2) mild motor and/or cognitive impairment, 3) moderate/severe motor and/or cognitive impairment, and 4) death.

Table 6. Motor outcome - 4 level classification

GMFCS

	**0**	**0.5**	**1**	**2**	**3**	**4**	**5**
**No CP**	None	None	Mild	Moderate	Severe	Severe	Severe
**HP or DP**	Mild	Mild	Moderate	Moderate	Severe	Severe	Severe
**QP**	Moderate*	Moderate*	Severe*	Severe	Severe	Severe	Severe

** It is unlikely that a child with quadriparetic CP will have a GMFCS of 0-1. However, this scenario is possible in cases of bilateral hemiparesis in which arms are more affected than legs. In such cases, the bilateral nature of the deficit, and the significant neurologic abnormalities that are noted on a standardized neurologic examination, warrant a designation of moderate/severe neurodevelopmental impairment.*

QP: quadriplegic; HP: hemiplegic; DP: diplegic

Table 7. Cognitive outcome - 4 level classification

**Cognitive Deficit**	**Bayley II MDI score (Hypothermia trials)**	**Bayley III Cognitive score (PENUT, HEAL)**
**Severe (> 3 SD)**	≤ 55	≤ 70
**Moderate (2-3 SD)**	> 55 and ≤ 70	70-84
**Mild (1-2 SD)**	> 70 and ≤ 85	85-89
**None**	> 85	≥ 90

9.3 DATA ANALYSES

9.3.1 Primary outcome analyses

The primary analysis will be a test of equality of the rate of the primary outcome (death or neurodevelopmental impairment (NDI)) across the two randomized investigational groups. Specifically, we will use a likelihood ratio test based on logistic regression, with stratification by

recruitment center and HIE severity. We will perform Intention to Treat analysis and expect minimal non-compliance due to the nature of the intervention in relation to in-patient care. For the primary endpoint, we expect uniform and complete ascertainment of death but may not evaluate all subjects for developmental impairment. We plan to perform a primary analysis based on complete cases and will exclude those subjects for whom vital status is known (alive) but NDI cannot be assessed. Sensitivity analysis will use multiple imputation to evaluate the potential impact of any missing data. Secondary analysis will consider an ordered categorical two-year status measure (death/severe or moderate impairment/mild impairment/normal), and analysis will be based on generalized Wilcoxon tests or regression models for ordered categorical outcomes such as the proportional odds model. Secondary quantitative measures include MRI-based injury score using the Washington University Standardized Scoring System. For these endpoints, a stratified t-test provides inference regarding the mean response across the treatment groups. We will adjust all secondary outcome analyses for recruitment site and HIE severity using regression methods.

9.3.2 Secondary Outcomes

Our key secondary long-term outcome is an ordered categorical 24-month status measure that classifies subjects as: dead; moderate or severe impairment; mild impairment; and normal. Use of this measure allows a detailed assessment of potential shifts in the distribution of outcomes toward improved status associated with treatment. Statistical analysis of this outcome will use a generalization of the Wilcoxon test that controls for recruitment site and HIE severity. Regression models for ordered categorical outcomes can also be used to provide adjusted treatment effect estimates.

9.3.3 Exploratory Analyses

Biomarker Prognostic Analysis. We will consider two main classes of potential predictors of 24-month status: neuroimaging measures and inflammatory markers. Interest is in the prognostic potential of individual and/or combined biomarker measurements. Given that the primary outcome is a binary measure (NDI), we will evaluate the predictive potential of individual quantitative measures using ROC curves showing the full potential of sensitivity and specificity across marker cut points. We will compute ROC curves for the (4) primary neuroimaging measures, and separately for individual inflammatory markers. We will derive two multivariate predictive models: using the inflammatory markers and using the MRI and MRS measures. We will use AIC and 10-fold cross-validation to develop and validate predictive models. A final multivariate model will combine markers from both MR and inflammatory measures, and 10-fold cross-validation will permit inference in the incremental value of adding markers in combination by comparing ROC curves and associated area under the ROC curve (AUC). Evaluation of whether treatment modifies the prognostic potential of biomarkers can be conducted by testing for the interaction between treatment status and individual biomarkers in predictive models for 2-year outcomes.

9.3.4 Analyses of Safety Data

Clinical safety data includes SAEs and clinical laboratory markers both from the hospitalization and study intervention period and from the long-term follow-up period. Safety event rates will be tabulated by study group and compared separately for each SAE using a multivariable logistic regression model, with adjustment for randomized treatment group, and randomization stratification factors of clinical recruitment site and HIE severity (moderate/severe). We will

additionally create a per-patient aggregate count of SAEs and evaluate the rates between groups using a Poisson regression model with robust standard errors and adjustment for randomization stratification factors. For any safety events that occur infrequently (<2%), we will use Fisher's exact test to compare the rates between treatment groups. Because these are critically ill newborns, we anticipate that the majority of safety events will occur during the initial inpatient and treatment period. As a secondary analysis, we will therefore summarize and analyze safety events that occur during the initial hospitalization and post-discharge time periods separately. Statistical significance is defined conservatively at the alpha=0.05 level with no correction for multiple outcomes.

Laboratory tests of organ injury are measured at baseline and at age 2-3 days as part of routine care. We will compare laboratory test data measured between study day 2 and 3 between treatment groups using analysis of covariance (ANCOVA) regression model, with adjustment for treatment group, the laboratory value measured at baseline, and the time between laboratory measurements. We will use a similar analytic approach to compare vital signs and growth parameters between treatment groups.

9.3.5 Neuroimaging and circulating biomarker analyses

Circulating biomarkers of inflammation and brain injury. Inflammation is thought to play an important role in HIE and CP.[144-147] Biomarkers of inflammation that we measure include: interleukin (IL)-1β, IL-6 and IL-8 and TNF-α. Putative biomarkers of brain injury in neonatal HIE include: glial fibrillary acidic protein (GFAP), ubiquitin C-terminal hydrolase-L1 (UCH-L1), S100B, Tau, and neuron-specific enolase (NSE). We will select a random subset of 200 subjects (100 treated and 100 controls with each group, for example, including both moderate and severe HIE) to measure circulating biomarkers of inflammation and brain injury. We will collect 3 plasma samples from each infant at the following time points based on hour of age: < 24 hours; Study Day 2; Study Day 4. Our analysis will focus on time-specific comparisons of the mean biomarker measure across treatment groups using appropriate regression methods while controlling for site and HIE severity. In addition, we will conduct longitudinal analysis using linear mixed models[148] that permit an omnibus test across all four measurement times, and allow inference on differential rates of change across treatment groups.

9.3.6 Compliance, Retention, and Missing Data

Adherence and retention. Our major analyses are based on the modified ITT principle. We do not anticipate that non-adherence will be a major issue since the treatment is directly observed during a short in-hospital time frame. We will assess non-compliance, with particular focus on study treatment dosing and timing. If there are more than minimal issues, that will justify quantifying and characterizing non-adherence and doing a per-protocol analysis.

In addition, site selection included having a committed neonatology follow-up program, and we expect >90% retention.

9.3.6.1 Missing data and dropouts.

Prevention: We will strive to sustain excellent participant involvement throughout the study and we have achieved 90%+ follow-up rates in numerous prior studies. The UW DCC will generate automated nightly reports, available to staff at the study sites, identifying these fields with a request to discuss and prevent further missingness.

Important data elements will be prospectively monitored to examine patterns of missingness.

Handling: We will conduct a missing data analysis to describe and characterize enrolled participants who do not provide further response due to attrition or dropout. We will use inverse probability weighting in secondary analysis for each response regression model (e.g. Generalized Estimating Equations) to inflate the weights of cases that are under-represented in the analysis due to selective attrition and/or non-participation. We will also conduct sensitivity analyses using 10-fold multiple imputation to assess the robustness of the results when missing data are imputed.

Statistical uncertainty: Given the type of missing data we expect in the proposed study, missing completely at random (MCAR) or missing at random (MAR), both methods we propose to utilize for missing data properly account for statistical uncertainty due to missingness and will provide accurate confidence interval coverage.

Tracking and reporting: The study web-based portal identifies when patients enter the follow-up interview window and when interviews are complete. Reasons for dropout will be systematically documented in the study database. In final manuscripts and analyses, the number of non-responders will be enumerated by study arm according to CONSORT guidelines.

Sensitivity analysis: We will assess the sensitivity of inferences made from missing data methods first by using the two previously described methods for dealing with missing data, and secondly by imputing missing data under both pessimistic and optimistic scenarios to provide bounds on the statistical uncertainty. The characteristics of non-responders will be summarized in our final report and we will present the sensitivity of the treatment effect due to missing data.

9.4 SAMPLE SIZE AND ACCRUAL

9.4.1 Primary Outcome Sample Size Calculations:

Our proposed HEAL sites report an overall mortality rate of 14%. Using three large sites that participated in the phase II study (UCSF, Wash U, CNMC) we can also estimate the rates of neurodevelopmental impairment: death = 14%; moderate-severe impairment = 18%; mild impairment = 17%; and normal = 51%. Therefore, we anticipate a **control primary outcome rate of 49%** (death or NDI).

Non-human data informing treatment effect size: A recent study with nonhuman primates (*Macaca nemestrina)* compared animals experiencing 15-18 minutes of umbilical cord occlusion that were then treated with either saline (n=14), therapeutic hypothermia (n=9), or therapeutic hypothermia and multiple doses of Epo.[21] Among animals treated with saline 8/14 = 57% died or had NDI. Among animals treated with hypothermia (HT) alone 7/9 = 78% died or had NDI, while among animals treated with hypothermia and Epo (HT+Epo) only 5/12 = 42% were observed to die or have NDI. Results from Figure 2 of Traudt et al.[149] (2013) show the number of animals in each outcome category by treatment group. These data suggest a risk ratio of 0.53 (95% CI: 0.25, 1.14) comparing HT+Epo to HT alone, and a risk ratio of 0.73 (95% CI: 0.32, 1.64) comparing HT+Epo

versus saline. Therefore, animal data support an Epo effect that optimistically corresponds to a 50% reduction, and that is conservatively associated with a 27% reduction in the primary outcome rate.

Phase I data informing treatment outcome rates: Given that the primary outcome is based on a 22-26 month assessment we can rely on a recently completed long-term follow-up from a phase I study[84] in which 24 cooled infants were given multiple doses of Epo ranging from 250U/kg to 2500U/kg[24]. At 22-26 months of age n=22 subjects were followed, and 0/22 subjects died, 1/22 had moderate-severe NDI, and 6/22 had mild NDI. This study suggests an **overall primary outcome rate of 7/22 = 31.8%** (exact confidence interval = 14%-55%) for our planned intervention group. We recognize that the dosing of Epo was not optimized in the phase I trial, and our proposed study will use multiple doses of 1000U/kg for all subjects which has been shown to yield plasma concentrations observed to be neuroprotective in animal studies.

Phase II data informing treatment outcome rates: In our phase II NEATO trial, a total of n=50 subjects were randomized to Epo (n=24) or placebo (n=26). We find substantial differences in the MRI injury severity distribution with 95% of Epo treated subjects having no injury or mild injury as compared to only 56% of control subjects. Using a recently submitted study from Trivedi, et al., we can then link the MRI injury severity category to expected Bayley III Cognitive scores at 24 months. Using our NEATO data and Trivedi's data we calculate a predicted mean (SD) of 97.9 (11.7) among Epo treated subjects, and a mean (SD) of 91.1 (15.2) among controls. The predicted 6.8-point mean difference for Bayley III Cognitive scores is consistent with our observed 6.3-point difference in mean WIDEA scores at 6 months among NEATO subjects. Predicted Bayley III Cognitive distributions lead to an expected 25.1% of subjects with a cognitive score of < 90 among Epo treated subjects, and 47.1% among controls. Incorporating expected death rates of 12% and 14% respectively for Epo treated and controls leads to an **expected primary outcome rate of 34.1% among treated** and 54.5% among controls. We acknowledge that our primary outcome is based on both Bayley III Cognitive scores, and clinical assessments of CP and GMFCS level, but expect Bayley status to be the major case indicator.

Table 8. Distribution of outcomes in previous studies.

	**NEATO Epo (n=22)**	**NEATO Placebo (n=25)**	**Bayley III Cognitive*****	***** Trivedi et al. (submitted)**
**Injury Severity**			Mean	Std. Dev
**None (0)**	8 (36%)	3 (12%)	96	(8)
**Mild (1-11)**	13 (59%)	11(44%)	100	(13)
**Moderate (12-32)**	1 (5%)	6 (24%)	85	(18)
**Severe (>32)**	0 (0%)	5 (20%)	76	(19)
**Predicted Bayley III Mean (S.D)**	97.9 (11.7)	91.1(15.2)		
Predicted %<90	25.1%	47.1%		
Predicted death/NDI	34.1%	54.5%		

In addition, Cheong et al. (2012)[150] evaluated the correlation between MRI findings obtained within 10 days of birth and 2-year clinical status outcomes for participants in the ICE trial (Infant Cooling Evaluation). Specifically, basal ganglia and thalamus (BGT) injuries were classified as abnormal if moderate/severe abnormalities were noted on T1- and T2-weighted images. In our phase II trial, we find only 4.5% (1/22) among Epo treated subjects have BGT injury as compared to 20% (5/25) among control subjects. Cheong et al. (2012) estimates that the probability of death/NDI at two years is 88% (PPV) for BGT abnormal subjects as compared to 32% (1-NPV) for BGT normal subjects. Applying these rates to our phase II BGT results yields **expected death/NDI rates of 34.5% among Epo treated**, and 43.2% among controls. For the ICE trial, the overall death/NDI rate for ICE hypothermia subjects was 51%, which is approximately the same as our expected control rate.

Therefore, based on animal data, phase I data, and projections from phase II data we expect primary outcome rates from 31-35% among Epo treated subjects with a protective relative risk of 0.65 to 0.71.

<u>Power and Sample Size for Primary Outcome</u>: In order to determine the necessary sample size for efficacy evaluation we need to formulate assumptions for the primary outcome rate in the Epo treated and control groups. The primary outcome measure is the composite rate of death or NDI, and current cohort studies suggest that the primary outcome occurs among 49% of infants treated with hypothermia alone (standard of care). Based on human data presented in Rogers et al.[24] (2014), animal studies including Traudt et al.[149], and the NEATO phase II data we assume that 33% of treated infants will die or have NDI, corresponding to a relative risk of 0.67. Assuming an intervention rate of 33% yields greater than 90% power, while we have 88% power for an alternative of 34%. In order to compute power, we assume a 90% follow-up rate with n=225/250 subjects evaluated in each arm.

Table 9. Power analysis for the primary outcome assuming n=500 patients randomized and 10% loss to follow-up.

Control	Intervention	Relative Risk	Power
49%	32%	0.65	95%
49%	33%	0.67	92%
49%	34%	0.69	88%
49%	35%	0.71	83%

9.4.2 Sample size for efficacy secondary outcomes

Our key secondary long-term outcome is an ordered categorical 24-month status measure that classifies subjects as: dead; moderate or severe impairment; mild impairment; and normal. Use of this measure allows a detailed assessment of potential shifts in the distribution of outcomes toward improved status associated with treatment. Statistical analysis of this outcome will use a generalization of the Wilcoxon test that controls for recruitment site and HIE severity. Regression

models for ordered categorical outcomes can also be used to provide adjusted treatment effect estimates.

To consider power we have used 2013 data from three large sites participating in the current phase II trial (UCSF, Wash U, CNMC) to estimate the distribution of outcomes within the control group. We calculate power for the stratified Wilcoxon test assuming a set of alternatives that are consistent with the assumption of a 33% rate of death or impairment (Scenarios 1 and 2) or a 32% rate (Scenarios 3 and 4) associated with intervention that was used to power the primary analysis. We assume a small effect on the death rate, and compute power for alternative shifts in the distribution of outcomes. For example, in Scenario 1 we assume no reduction in the death rate, and an 8% reduction in the moderate/severe category (relative risk = 0.55), and an 8% reduction in the rate of mild impairment (relative risk = 0.50) which yields 81% power using a two-sided alpha=0.05 test. Scenario 3 considers a larger reduction in the rate of moderate/severe and a smaller reduction in the mild category and yields 90% power. Scenarios 3 and 4 consider a 32% overall rate of impairment or death and yield 90% or greater power. Therefore, our study is adequately powered to detect modest but clinically important shifts in the outcome distribution.

Table 10. Power analysis for a four-level outcome measure with n=500 patients randomized and assuming 10% loss to follow-up.

	**Neurodevelopmental Impairment**				
	**Normal**	**Mild**	**Moderate/Severe**	**Mortality**	**Power**
**Placebo Arm**	51%	17%	18%	14%	--
**Epo Arm**					
Scenario 1	67%	9%	10%	14%	81%
Scenario 2	67%	11%	10%	12%	86%
Scenario 3	68%	9%	9%	14%	86%
Scenario 4	68%	11%	9%	12%	92%

Given the a priori hypothesis that treatment effect may differ according to gender or HIE severity we will conduct a pair of subgroup analyses that assesses treatment effects separately for males and for females, and separately for moderate and severe HIE. Subgroup specific treatment effects will be computed and inference will be based on a single Covariate-by-Treatment test for interaction using logistic regression.

9.4.3 Sample size for MRI/MRS biomarkers

For imaging measures, we will have data for all subjects. Assuming a 10% missing data rate we have 80% power to detect a difference in the mean of secondary outcomes across treatment groups of 0.26 SDs. For the inflammation and brain injury biomarkers, we will have a total of 200 subjects and have 80% power to detect a mean difference across the treatment groups of 0.4 SDs.

9.4.4 Sample size for plasma biomarkers of brain injury and inflammation

A sample size of 91 neonates per group would have been required to detect a 0.5 SD difference on the log scale with a single observation per neonate with 80% power while controlling for a Type I

error of 0.0125 (4 markers, Bonferroni). However, since we have 4 samples per neonate, a sample size of 100 neonates per treatment group (with 400 samples per group, but correlated) will be more than sufficient to attain >80% power.

9.4.5 Achieving the sample size

The plan is to enroll and randomize 500 patients over a 36-month period. The 17 sites that were included in the original study proposal cooled a total of 530 HIE infants in the year 2014. From the NEATO phase II randomized controlled trial study, we estimate that at least 40-45% of these infants would be eligible and will consent for a phase III trial, yielding at least 16 enrolled infants per month. With a further conservative estimate of 14 per month allowing for slower enrollment during start-up, we can enroll 500 patients in 36 months.

9.5 DATA MONITORING

We will monitor the accuracy of data entry by the sites both internally and externally. We will review study data on arrival for completeness. We will then subject each submitted data set to a set of preliminary checks to search for values that are out-of-range or otherwise inappropriate. Using the Patient Monitoring Report, a subset of all data points in the CRFs will be compared with the medical record for 20-25% of enrolled subjects. Any outstanding data queries will be resolved with the research coordinator at the time of the site-monitoring visit. After each study site monitoring visit, a report will be prepared and copies sent to the Study File, the study PIs (Y. Wu and S. Juul), the site PI, and the site coordinator. The quality and completeness of other deliverables (blood samples, MRIs) will be monitored.

9.6 DATA AND SAFETY MONITORING PLAN (DSMP)

The DSMB will review the accruing data to: 1) ensure that the study is adequately enrolling; 2) to ensure that there are no serious safety concerns; and 3) to assess whether the study efficacy appears overwhelming. The DSMB will be assigned by NINDS. The research coordinator at each site will monitor each subject weekly for the presence of any complications. Serious adverse events will be brought to the attention of the DSMB, and if appropriate, the IRB, in writing. An independent medical monitor will review all cases of serious adverse events.

As part of this DSMP, we will perform continuous and interim analysis of accruing safety data. We have defined potentially treatment (Epo) related serious adverse events (SAEs) that will be monitored throughout the course of the study. Specifically, for SAEs we will compare absolute rates to expected rates based on published data for similar newborns, and will seek careful DSMB review and guidance when observed rates exceed pre-specified thresholds. In addition, at planned interim analysis we will formally compare the event rates across the two treatment groups using appropriate small sample methods such as Fisher's exact test. The DCC PI will remain masked to assignment while the study staff statistician will not.

The primary outcome of the study is a composite endpoint of mortality or NDI at 24 months of age. Therefore, monitoring the primary outcome for treatment efficacy or futility is challenging. Based on enrollment plans, the majority of patients will have been randomized by the time NDI is assessed at 24 months for any participants, and therefore we do not expect to be able to conduct a first interim analysis prior to the completion of enrollment. We expect to have the primary outcome evaluated on the first quarter of subjects (n=125) after 34 months of recruitment at which point we expect to have randomized n=450 of the total n=500 subjects (90% enrollment completed). Therefore, any actions that a DSMB might take to prevent subsequent patients from receiving an ineffective treatment (futility) or

to make available a useful treatment (efficacy) will not have a direct impact on patients participating in this study. As a surrogate for long-term 24-month clinical efficacy on NDI, one alternative would be to monitor directly early MRI results as intermediate outcomes or surrogates. However, a large study published by Cheong et al.,[150] found that early MRI measurements had poor sensitivity (27-60%) for accurately predicting death or NDI at 2 years. Therefore, we do not recommend using early MRI measures as a surrogate for the long-term outcome for monitoring treatment efficacy. Given that we are not conducting interim analysis directly on the primary outcome measure for efficacy or futility, we do not make adjustments to sample size or statistical power to account for interim alpha spending.

Our primary objective for interim analyses is therefore to allow for careful and continued monitoring of mortality and safety outcomes. We propose to conduct formal statistical analysis and inference for mortality at three interim and one final analysis time. We will continue to present mortality and SAE data from all available follow-up data, but we expect most treatment-related safety events to occur within the first three months of follow-up. We will conduct formal safety evaluation at 6, 12, 18, and 24 months following the start of enrollment, where approximately 25, 50, 75, and 100% of the study cohort will have been randomized and followed for at least 3 months. As part of each interim analysis, we plan to monitor mortality as a primary safety endpoint and will control the overall significance level using O'Brien-Fleming boundaries (net alpha=0.05 significance, accounting for three interim and one final analysis). The DSMB will also monitor all other SAEs utilizing the same O'Brien-Fleming sequential monitoring boundaries without further adjustment for multiple comparisons, but allowing for flexibility to continue the study if the O'Brien-Fleming boundary is reached on a secondary SAE endpoint.

Table 11. O'Brien-Fleming Monitoring Boundaries

Enrollment	Monitoring p-value: Death
25%	0.000014734
50%	0.0030359
75%	0.016248
100%	0.030701

EXAMPLE SAFETY MONITORING TABLE

Shell Table 1. Closed Report: Serious adverse event (SAE) incidence rate (number of events/number at risk) and threshold for action.

Serious Adverse Events	Group A	Group B	Expected (Range)	Threshold for Action	p-value*
Systemic hypertension			0.2-3.0[151-153]	6%	
Polycythemia			1.0-5.0%[154]	10%	
Disseminated intravascular coagulation (DIC)			11.0-45.0%[3-7]	50%	
Major venous or arterial thrombosis			0-2.0%[4,6,155]	8%	
Pulmonary hypertension			6.0-22.0%[3,4,7,156]	30%	
Intracranial hemorrhage			7.9-31.3%[6,7]	37%	
Cardiopulmonary arrest			0.002-0.13%[157]	5%	

Other unexpected life-threatening event			1.0%[4]	5%	
Death			8.1-35.1%[3-7,155,158,159]	40%	

* P-value calculated for the rate ratio comparing treatment Group A to Group B.

In addition, we will provide a table comparing complications of HIE between the groups.

Data Safety Monitoring Schedule: Our target enrollment is n=500 which is expected to accrue during the first 4.0 years of the trial. Therefore, we will enroll approximately 83 subjects every 6 months. Our planned DSMB safety analyses will occur every 6 months after trial initiation. At the first safety evaluation, we expect to have 83 subjects enrolled, but would only have discharge outcomes on the first 60 subjects. Each subsequent 6-month period would increase the number of babies by 83 leading to the following cumulative number of subjects available for analysis. Safety evaluation will be based on all available follow-up but we expect the majority of SAEs and AEs to occur during the neonatal hospitalization and therefore within one month of age (2-4 weeks) in the majority of cases.

Table 12. Cumulative number of subjects evaluated for safety events and for the long-term efficacy outcome

**Month**	**6**	**12**	**18**	**24**	**30**	**36**	**42**	**48**	**54**	**60**	**66**
**Safety (1 mo)**	60	143	226	309	392	475	500				
**Efficacy (24 mo)**	0	0	0	0	60	143	226	309	392	475	500

10 DATA COLLECTION, SITE MONITORING, AE REPORTING

10.1 RECORDS TO BE KEPT

All data will be entered into CRFs in the HEAL Portal that are developed specifically for the study, using a REDCap platform. These forms will include screening, enrollment and follow-up data for infants. The infant's parent or legal guardian must read, understand, and sign an IRB approved informed consent form. Additionally, it will be recorded in the infant's medical record that the infant is involved in this study. The Investigator will retain the original signed consent form in a secured location. Investigators must provide all information required by the protocol on the CRFs in the HEAL Portal provided for the study.

At the DCC, all data received is identified by a unique subject ID. No subject identifiers are collected other than date of birth

10.1.1 Subject identifiers

Only the clinical site will have the Screening Log that maps the ID number, infant initials and randomization number to the subject's name and contact information at their site. At the clinical sites, all subject data are maintained in locked file cabinet or other secure locations with limited access by researchers and staff. Laboratory samples will be identified by a study ID number. A list of each sites' subjects will be kept separately in password protected or locked files within that site investigator's office.

10.1.2 Documentation

Each site must provide the CCC at the University of California, San Francisco with the following documents prior to study initiation. A copy of these documents must also be maintained throughout the study in the investigator's study files.

- IRB approved informed consent form
- Completed 1572 form, signed and dated CVs, proof of current CITI training or equivalent (Human Subjects Training), Good Clinical Practice certification, financial conflict of interest forms, and proof of current medical licensure for all investigators
- All IRB approvals and correspondence (approved revisions, protocol, advertisements, etc.)
- Copies of all correspondence pertaining to the study (excluding any budgetary matters)
- Copies of all serious adverse events submitted to the IRB
- Copy of all safety reports

The clinical site is responsible for maintaining all records (i.e., case report forms, original data, screening logs, signed informed consent forms, correspondence, etc.) until notified, in writing, by CCC, that these records are no longer needed. The Investigator must notify CCC project director if the site or records are relocated, if the investigator leaves the institution, etc. and a new address for the records must be provided.

10.2 ROLE OF DATA MANAGEMENT

The DCC will be at University of Washington, Seattle WA. The DCC will create an https secured web-based portal specially designed for the purposes of this study. The study portal will provide a highly-structured repository to store and process study data from e-CRFs, as well as to protect its integrity and confidentiality. This tool will assist the CCC by providing efficient protocol management and study enrollment and retention oversight. Access to the study portal will be granted by the DCC with differential access rights based on role. Users at a given site will only have access to data on study participants at their own site.

The DCC is responsible for:

- Providing tools for data collection, management and monitoring for the clinical sites
- Supporting a web page and portal and providing training for research staff
- Providing and managing secure log-in access to the study portal
- Biostatistics and analysis support

The DCC also has the responsibility to:

- Generate and distribute masked randomization assignments to clinical site pharmacies
- Ensure study drug accountability at all sites
- Provide data summaries and statistical analyses for the study
- Receive information about SAEs
- Generate semi-annual data and safety monitoring reports for independent assessment by the study DSMB

The DCC will facilitate user access by sponsoring UW NetIDs for each individual involved with the HEAL project. For each clinical recruitment site, the DCC will provide up to two initial training sessions to familiarize research staff and investigators with the web-based data management portal. As research staff turnover during the course of the study, data management training videos and a FAQ page will be accessible on the portal home page in order to ensure that new staff are facile with the system.

10.2.1 The Clinical Site responsibilities in data collection and management

10.2.1.1 Data Collection Protocol

- For screening and enrollment, the site will access the REDCap study portal via the web, provide screening information about the patient (Screening ID will be generated) and if eligible for randomization, receive the study participant number (Study ID).
- Study-related data, as outlined in the schedule of visits and evaluations above, will be entered online by site personnel directly into the CRFs within the REDCap study portal.

10.2.1.2 Description & Flow of Case Report Forms (CRFs)

- Copies (PDF versions) of all CRFs are printable from the study portal.
- CRFs can be printed and used to capture data, and later entered into the REDCap study portal.

10.2.2 Data Security and Confidentiality

The DCC employs procedural and technical controls to ensure the security, integrity and confidentiality of subject data that are in compliance with established regulations and standards for Information Technology Security. While only limited identifiable data will be collected by the DCC (date of birth, dates of service), this server room meets the technical requirements for HIPAA compliance and hosts other servers containing PHI. Multiple levels of data security are in place designed to prevent unauthorized access and limit authorized access to the computer systems and prevent data corruption and loss. These include firewall and network intrusion detection devices, malware protections, account and system security features, as well as written policies and procedures.

10.3 QUALITY ASSURANCE

We will monitor the accuracy of data entry by the sites both internally and externally. For internal monitoring, completed online CRFs (in the HEAL Portal) are reviewed on a regular basis, and issues are clarified as necessary with site coordinators who act as liaison between the sites and the DCC as needed.

PI's and Study Coordinators from the CCC will perform regular monitoring visits to every site while actively enrolling. Initial monitoring visits will be targeted for after the first 6-8 patients have completed data collection at a given site. A subset of all data points in the online CRFs will be compared with the medical record. Any outstanding data queries will attempt to be resolved with the research coordinator at the time of the visit. After each study site monitoring visit, a report will be prepared and copies sent to the Study File, the DCC, the study PIs, the site PI, the site coordinator and the CCC Study Coordinators.

As part of the overall quality assurance (QA) effort, we will examine various measures of study implementation across sites. In particular, recruitment, retention, data completeness, and measurement

precision will be tabulated and compared across sites and will be included in our web-based reports. QA efforts and site visits will be focused on any sites that show evidence of problems.

The DCC will use a web- and email-based reminder system to identify when patients enter a follow-up evaluation window and when evaluations are complete. Data managers from the DCC will assess data quality by running validation checks on a regular basis. Data fields are verified against documented expectations using suitable legal ranges, field requirements, reports, and other data consistency checks. Any discrepancies are reported back to the clinical sites for further review and correction. The reporting page of the study portal will contain a window-based reporting system that checks for the timely entry of data within the anticipated length of neonatal hospitalization (7-20 days) and the schedule of follow-up visits

10.3.1 Data Audit

After all subject data have been submitted and data queries addressed, the database will be considered ready to lock.

10.4 ADVERSE EXPERIENCE REPORTING

See **Table *5*** above for the flow chart reflecting AE and SAE reporting.

11 HUMAN SUBJECTS

11.1 INSTITUTIONAL REVIEW BOARD REVIEW AND INFORMED CONSENT

This protocol, the informed consent document, and any subsequent modifications will be reviewed and approved by the IRBs responsible for oversight of the study at each study site. Any substantial changes in protocol must be approved by the DSMB. The consent form will describe the purpose of the study, the procedures to be followed, Certificate of Confidentiality language, and the risks and benefits of participation. A list of key elements to be included in the consent will be prepared by the CCC, reviewed by the DCC, and reviewed and ultimately approved by the DSMB. The approved consent at UCSF will be used as a template for preparation of the consent at the sites. A copy of the signed consent form will be given to the parent or legal guardian, a copy placed in the patient's chart, and the original signed consent will be stored by the site coordinator.

11.2 SUBJECT CONFIDENTIALITY

All laboratory specimens, evaluation forms, reports, and other records that leave the site will be identified only by the Study Identification Number (Subject Study ID), as well as date and time for laboratory specimens, to maintain subject confidentiality. All records will be kept in a locked file cabinet. All computer entry and networking programs will be performed using Subject Study ID only. Clinical information will not be released without written permission of the subject, except as necessary for monitoring by the IRB, the FDA, the NINDS, and by the CCC and DCC.

11.3 STUDY MODIFICATION/DISCONTINUATION

The study may be modified or discontinued at any time by a local IRB, the NINDS, the DSMB, or the FDA as part of their duties to ensure that research subjects are protected. Any changes to the protocol require a written protocol amendment that must be approved by the Executive and Steering Committees and by the DSMB prior to implementation. Amendments that affect patient eligibility, study protocol, or consent changes require additional approval by the IRB at each site. These amendments, should they be required, will become a part of the protocol and maintained by the Investigator as part

of the study documentation. For amendments affecting only administrative aspects of the study that do not require formal IRB approval, the IRB at each of the sites must be informed of such changes. Other changes in the study conduct are not permitted. Any unforeseen changes must be recorded in the clinical study report.

12 PUBLICATION OF RESEARCH FINDINGS

Publication of the results of this trial will be governed by the policies and procedures developed by the Executive Committee. Any presentation, abstract, or manuscript will be made available for review by the NINDS prior to submission.

13 REFERENCES

1. Kurinczuk JJ, White-Koning M, Badawi N. Epidemiology of neonatal encephalopathy and hypoxic-ischaemic encephalopathy. *Early Hum Dev.* 2010;86(6):329-338.
2. Black RE, Cousens S, Johnson HL, et al. Global, regional, and national causes of child mortality in 2008: a systematic analysis. *Lancet.* 2010;375(9730):1969-1987.
3. Shankaran S, Laptook AR, Ehrenkranz RA, et al. Whole-body hypothermia for neonates with hypoxic-ischemic encephalopathy. *N Engl J Med.* 2005;353(15):1574-1584.
4. Gluckman PD, Wyatt JS, Azzopardi D, et al. Selective head cooling with mild systemic hypothermia after neonatal encephalopathy: multicentre randomised trial. *Lancet.* 2005;365(9460):663-670.
5. Jacobs SE, Morley CJ, Inder TE, et al. Whole-Body Hypothermia for Term and Near-Term Newborns With Hypoxic-Ischemic Encephalopathy: A Randomized Controlled Trial. *Arch Pediatr Adolesc Med.* 2011;165(8):692-700.
6. Simbruner G, Mittal RA, Rohlmann F, Muche R. Systemic hypothermia after neonatal encephalopathy: outcomes of neo.nEURO.network RCT. *Pediatrics.* 2010;126(4):e771-778.
7. Azzopardi DV, Strohm B, Edwards AD, et al. Moderate hypothermia to treat perinatal asphyxial encephalopathy. *N Engl J Med.* 2009;361(14):1349-1358.
8. Juul S. Erythropoietin in the central nervous system, and its use to prevent hypoxic-ischemic brain damage. *Acta Paediatr Suppl.* 2002;91(438):36-42.
9. Juul SE, McPherson RJ, Bammler TK, Wilkerson J, Beyer RP, Farin FM. Recombinant erythropoietin is neuroprotective in a novel mouse oxidative injury model. *Developmental neuroscience.* 2008;30(4):231-242.
10. Juul S. Recombinant erythropoietin as a neuroprotective treatment: in vitro and in vivo models. *Clin Perinatol.* 2004;31(1):129-142.
11. Demers EJ, McPherson RJ, Juul SE. Erythropoietin protects dopaminergic neurons and improves neurobehavioral outcomes in juvenile rats after neonatal hypoxia-ischemia. *Pediatric research.* 2005;58(2):297-301.
12. Dame C, Juul SE, Christensen RD. The biology of erythropoietin in the central nervous system and its neurotrophic and neuroprotective potential. *Biol Neonate.* 2001;79(3-4):228-235.
13. Reitmeir R, Kilic E, Kilic U, et al. Post-acute delivery of erythropoietin induces stroke recovery by promoting perilesional tissue remodelling and contralesional pyramidal tract plasticity. *Brain.* 2011;134(Pt 1):84-99.
14. McPherson RJ, Demers EJ, Juul SE. Safety of high-dose recombinant erythropoietin in a neonatal rat model. *Neonatology.* 2007;91(1):36-43.
15. Sola A, Rogido M, Lee BH, Genetta T, Wen TC. Erythropoietin after focal cerebral ischemia activates the Janus kinase-signal transducer and activator of transcription signaling pathway and improves brain injury in postnatal day 7 rats. *Pediatric research.* 2005;57(4):481-487.
16. Chang YS, Mu D, Wendland M, et al. Erythropoietin improves functional and histological outcome in neonatal stroke. *Pediatric research.* 2005;58(1):106-111.
17. Gonzalez FF, McQuillen P, Mu D, et al. Erythropoietin enhances long-term neuroprotection and neurogenesis in neonatal stroke. *Developmental neuroscience.* 2007;29(4-5):321-330.
18. Iwai M, Stetler RA, Xing J, et al. Enhanced oligodendrogenesis and recovery of neurological function by erythropoietin after neonatal hypoxic/ischemic brain injury. *Stroke.* 2010;41(5):1032-1037.

19. Kumral A, Uysal N, Tugyan K, et al. Erythropoietin improves long-term spatial memory deficits and brain injury following neonatal hypoxia-ischemia in rats. *Behav Brain Res.* 2004;153(1):77-86.
20. Sargin D, Friedrichs H, El-Kordi A, Ehrenreich H. Erythropoietin as neuroprotective and neuroregenerative treatment strategy: Comprehensive overview of 12 years of preclinical and clinical research. *Best Pract Res Clin Anaesthesiol.* 2010;24(4):573-594.
21. Traudt CM, McPherson RJ, Bauer LA, et al. Concurrent erythropoietin and hypothermia treatment improve outcomes in a term nonhuman primate model of perinatal asphyxia. *Developmental neuroscience.* 2013;35(6):491-503.
22. Zhu C, Kang W, Xu F, et al. Erythropoietin improved neurologic outcomes in newborns with hypoxic-ischemic encephalopathy. *Pediatrics.* 2009;124(2):e218-226.
23. Elmahdy H, El-Mashad AR, El-Bahrawy H, El-Gohary T, El-Barbary A, Aly H. Human recombinant erythropoietin in asphyxia neonatorum: pilot trial. *Pediatrics.* 2010;125(5):e1135-1142.
24. Rogers EE, Bonifacio SL, Glass HC, et al. Erythropoietin and Hypothermia for Hypoxic-Ischemic Encephalopathy. *Pediatr Neurol.* 2014:In Press.
25. Wu YW, Mathur AM, Chang T, et al. High-Dose Erythropoietin and Hypothermia for Hypoxic-Ischemic Encephalopathy: A Phase II Trial. *Pediatrics.* 2016;137(6).
26. Sarnat HB, Sarnat MS. Neonatal encephalopathy following fetal distress. A clinical and electroencephalographic study. *Arch Neurol.* 1976;33(10):696-705.
27. Graham EM, Ruis KA, Hartman AL, Northington FJ, Fox HE. A systematic review of the role of intrapartum hypoxia-ischemia in the causation of neonatal encephalopathy. *Am J Obstet Gynecol.* 2008;199(6):587-595.
28. Wu YW, Backstrand KH, Zhao S, Fullerton HJ, Johnston SC. Declining diagnosis of birth asphyxia in California: 1991-2000. *Pediatrics.* 2004;114(6):1584-1590.
29. Wu YW, Pham TN, Danielsen B, Towner D, Smith L, Johnston SC. Nighttime delivery and risk of neonatal encephalopathy. *Am J Obstet Gynecol.* 2011;204(1):37 e31-36.
30. Shankaran S, Pappas A, McDonald SA, et al. Childhood outcomes after hypothermia for neonatal encephalopathy. *N Engl J Med.* 2012;366(22):2085-2092.
31. Frymoyer A, Juul SE, Massaro AN, Bammler TK, Wu YW. High-dose erythropoietin population pharmacokinetics in neonates with hypoxic-ischemic encephalopathy receiving hypothermia. *Pediatric research.* 2017;81(6):865-872.
32. Villa P, Bigini P, Mennini T, et al. Erythropoietin selectively attenuates cytokine production and inflammation in cerebral ischemia by targeting neuronal apoptosis. *J Exp Med.* 2003;198(6):971-975.
33. Agnello D, Bigini P, Villa P, et al. Erythropoietin exerts an anti-inflammatory effect on the CNS in a model of experimental autoimmune encephalomyelitis. *Brain Res.* 2002;952(1):128-134.
34. Arvin B, Neville LF, Barone FC, Feuerstein GZ. The role of inflammation and cytokines in brain injury. *Neurosci Biobehav Rev.* 1996;20(3):445-452.
35. Wang L, Zhang Z, Wang Y, Zhang R, Chopp M. Treatment of stroke with erythropoietin enhances neurogenesis and angiogenesis and improves neurological function in rats. *Stroke.* 2004;35(7):1732-1737.
36. Yamaji R, Okada T, Moriya M, et al. Brain capillary endothelial cells express two forms of erythropoietin receptor mRNA. *Eur J Biochem.* 1996;239(2):494-500.
37. Chong ZZ, Kang JQ, Maiese K. Angiogenesis and plasticity: role of erythropoietin in vascular systems. *J Hematother Stem Cell Res.* 2002;11(6):863-871.

38. Bernaudin M, Marti HH, Roussel S, et al. A potential role for erythropoietin in focal permanent cerebral ischemia in mice. *J Cereb Blood Flow Metab.* 1999;19(6):643-651.
39. Mu D, Chang YS, Vexler ZS, Ferriero DM. Hypoxia-inducible factor 1alpha and erythropoietin upregulation with deferoxamine salvage after neonatal stroke. *Exp Neurol.* 2005;195(2):407-415.
40. Wallach I, Zhang J, Hartmann A, et al. Erythropoietin-receptor gene regulation in neuronal cells. *Pediatric research.* 2009;65(6):619-624.
41. Sugawa M, Sakurai Y, Ishikawa-Ieda Y, Suzuki H, Asou H. Effects of erythropoietin on glial cell development; oligodendrocyte maturation and astrocyte proliferation. *Neurosci Res.* 2002;44(4):391-403.
42. Nagai A, Nakagawa E, Choi HB, Hatori K, Kobayashi S, Kim SU. Erythropoietin and erythropoietin receptors in human CNS neurons, astrocytes, microglia, and oligodendrocytes grown in culture. *J Neuropathol Exp Neurol.* 2001;60(4):386-392.
43. Chong ZZ, Kang JQ, Maiese K. Erythropoietin fosters both intrinsic and extrinsic neuronal protection through modulation of microglia, Akt1, Bad, and caspase-mediated pathways. *Br J Pharmacol.* 2003;138(6):1107-1118.
44. Bernaudin M, Nedelec AS, Divoux D, MacKenzie ET, Petit E, Schumann-Bard P. Normobaric hypoxia induces tolerance to focal permanent cerebral ischemia in association with an increased expression of hypoxia-inducible factor-1 and its target genes, erythropoietin and VEGF, in the adult mouse brain. *J Cereb Blood Flow Metab.* 2002;22(4):393-403.
45. Vexler ZS, Sharp FR, Feuerstein GZ, et al. Translational stroke research in the developing brain. *Pediatr Neurol.* 2006;34(6):459-463.
46. Yis U, Kurul SH, Kumral A, et al. Effect of erythropoietin on oxygen-induced brain injury in the newborn rat. *Neuroscience letters.* 2008;448(3):245-249.
47. Chong ZZ, Kang JQ, Maiese K. Apaf-1, Bcl-xL, cytochrome c, and caspase-9 form the critical elements for cerebral vascular protection by erythropoietin. *J Cereb Blood Flow Metab.* 2003;23(3):320-330.
48. Wei L, Han BH, Li Y, Keogh CL, Holtzman DM, Yu SP. Cell death mechanism and protective effect of erythropoietin after focal ischemia in the whisker-barrel cortex of neonatal rats. *J Pharmacol Exp Ther.* 2006;317(1):109-116.
49. Sun Y, Calvert JW, Zhang JH. Neonatal hypoxia/ischemia is associated with decreased inflammatory mediators after erythropoietin administration. *Stroke.* 2005;36(8):1672-1678.
50. Juul SE, Beyer RP, Bammler TK, McPherson RJ, Wilkerson J, Farin FM. Microarray analysis of high-dose recombinant erythropoietin treatment of unilateral brain injury in neonatal mouse hippocampus. *Pediatric research.* 2009;65(5):485-492.
51. Kumral A, Tugyan K, Gonenc S, et al. Protective effects of erythropoietin against ethanol-induced apoptotic neurodegenaration and oxidative stress in the developing C57BL/6 mouse brain. *Brain Res Dev Brain Res.* 2005;160(2):146-156.
52. Chattopadhyay A, Choudhury TD, Bandyopadhyay D, Datta AG. Protective effect of erythropoietin on the oxidative damage of erythrocyte membrane by hydroxyl radical. *Biochem Pharmacol.* 2000;59(4):419-425.
53. Genc S, Koroglu TF, Genc K. Erythropoietin and the nervous system. *Brain Res.* 2004;1000(1-2):19-31.
54. Zacharias R, Schmidt M, Kny J, et al. Dose-dependent effects of erythropoietin in propofol anesthetized neonatal rats. *Brain Res.* 2010;1343:14-19.

55. Genc K, Genc S, Baskin H, Semin I. Erythropoietin decreases cytotoxicity and nitric oxide formation induced by inflammatory stimuli in rat oligodendrocytes. *Physiol Res.* 2006;55(1):33-38.
56. Mizuno K, Hida H, Masuda T, Nishino H, Togari H. Pretreatment with low doses of erythropoietin ameliorates brain damage in periventricular leukomalacia by targeting late oligodendrocyte progenitors: a rat model. *Neonatology.* 2008;94(4):255-266.
57. Vitellaro-Zuccarello L, Mazzetti S, Madaschi L, et al. Chronic erythropoietin-mediated effects on the expression of astrocyte markers in a rat model of contusive spinal cord injury. *Neuroscience.* 2008;151(2):452-466.
58. Li L, Jiang Q, Ding G, et al. MRI identification of white matter reorganization enhanced by erythropoietin treatment in a rat model of focal ischemia. *Stroke.* 2009;40(3):936-941.
59. Iwai M, Cao G, Yin W, Stetler RA, Liu J, Chen J. Erythropoietin promotes neuronal replacement through revascularization and neurogenesis after neonatal hypoxia/ischemia in rats. *Stroke.* 2007;38(10):2795-2803.
60. Ransome MI, Turnley AM. Systemically delivered Erythropoietin transiently enhances adult hippocampal neurogenesis. *J Neurochem.* 2007;102(6):1953-1965.
61. Bocker-Meffert S, Rosenstiel P, Rohl C, et al. Erythropoietin and VEGF promote neural outgrowth from retinal explants in postnatal rats. *Invest Ophthalmol Vis Sci.* 2002;43(6):2021-2026.
62. Yang Z, Covey MV, Bitel CL, Ni L, Jonakait GM, Levison SW. Sustained neocortical neurogenesis after neonatal hypoxic/ischemic injury. *Ann Neurol.* 2007;61(3):199-208.
63. Shingo T, Sorokan ST, Shimazaki T, Weiss S. Erythropoietin regulates the in vitro and in vivo production of neuronal progenitors by mammalian forebrain neural stem cells. *J Neurosci.* 2001;21(24):9733-9743.
64. Osredkar D, Sall JW, Bickler PE, Ferriero DM. Erythropoietin promotes hippocampal neurogenesis in in vitro models of neonatal stroke. *Neurobiol Dis.* 2010;38(2):259-265.
65. Dzietko M, Felderhoff-Mueser U, Sifringer M, et al. Erythropoietin protects the developing brain against N-methyl-D-aspartate receptor antagonist neurotoxicity. *Neurobiol Dis.* 2004;15(2):177-187.
66. Wang L, Chopp M, Gregg SR, et al. Neural progenitor cells treated with EPO induce angiogenesis through the production of VEGF. *J Cereb Blood Flow Metab.* 2008;28(7):1361-1368.
67. Yin D, Kawabata H, Tcherniamtchouk O, Huynh T, Black KL, Koeffler HP. Glioblastoma multiforme cells: expression of erythropoietin receptor and response to erythropoietin. *Int J Oncol.* 2007;31(5):1193-1198.
68. Wang L, Zhang ZG, Zhang RL, et al. Matrix metalloproteinase 2 (MMP2) and MMP9 secreted by erythropoietin-activated endothelial cells promote neural progenitor cell migration. *J Neurosci.* 2006;26(22):5996-6003.
69. Digicaylioglu M, Lipton SA. Erythropoietin-mediated neuroprotection involves cross-talk between Jak2 and NF-kappaB signalling cascades. *Nature.* 2001;412(6847):641-647.
70. Lee ST, Chu K, Sinn DI, et al. Erythropoietin reduces perihematomal inflammation and cell death with eNOS and STAT3 activations in experimental intracerebral hemorrhage. *J Neurochem.* 2006;96(6):1728-1739.
71. Park KI, Himes BT, Stieg PE, Tessler A, Fischer I, Snyder EY. Neural stem cells may be uniquely suited for combined gene therapy and cell replacement: Evidence from engraftment of

Neurotrophin-3-expressing stem cells in hypoxic-ischemic brain injury. *Exp Neurol.* 2006;199(1):179-190.
72. Xiong T, Qu Y, Mu D, Ferriero D. Erythropoietin for neonatal brain injury: opportunity and challenge. *Int J Dev Neurosci.* 2011.
73. Kumral A, Ozer E, Yilmaz O, et al. Neuroprotective effect of erythropoietin on hypoxic-ischemic brain injury in neonatal rats. *Biol Neonate.* 2003;83(3):224-228.
74. Aydin A, Genc K, Akhisaroglu M, Yorukoglu K, Gokmen N, Gonullu E. Erythropoietin exerts neuroprotective effect in neonatal rat model of hypoxic-ischemic brain injury. *Brain Dev.* 2003;25(7):494-498.
75. Matsushita H, Johnston MV, Lange MS, Wilson MA. Protective effect of erythropoietin in neonatal hypoxic ischemia in mice. *Neuroreport.* 2003;14(13):1757-1761.
76. Sun Y, Zhou C, Polk P, Nanda A, Zhang JH. Mechanisms of erythropoietin-induced brain protection in neonatal hypoxia-ischemia rat model. *J Cereb Blood Flow Metab.* 2004;24(2):259-270.
77. Spandou E, Papoutsopoulou S, Soubasi V, et al. Hypoxia-ischemia affects erythropoietin and erythropoietin receptor expression pattern in the neonatal rat brain. *Brain Res.* 2004;1021(2):167-172.
78. Solaroglu I, Solaroglu A, Kaptanoglu E, et al. Erythropoietin prevents ischemia-reperfusion from inducing oxidative damage in fetal rat brain. *Childs Nerv Syst.* 2003;19(1):19-22.
79. Hagberg H, Bona E, Gilland E, Puka-Sundvall M. Hypoxia-ischaemia model in the 7-day-old rat: possibilities and shortcomings. *Acta Paediatr Suppl.* 1997;422:85-88.
80. Kellert BA, McPherson RJ, Juul SE. A comparison of high-dose recombinant erythropoietin treatment regimens in brain-injured neonatal rats. *Pediatric research.* 2007;61(4):451-455.
81. Gonzalez FF, Abel R, Almli CR, Mu D, Wendland M, Ferriero DM. Erythropoietin sustains cognitive function and brain volume after neonatal stroke. *Developmental neuroscience.* 2009;31(5):403-411.
82. Larpthaveesarp A, Georgevits M, Ferriero DM, Gonzalez FF. Delayed erythropoietin therapy improves histological and behavioral outcomes after transient neonatal stroke. *Neurobiol Dis.* 2016;93:57-63.
83. Iwai M, Stetler RA, Xing J, et al. Enhanced oligodendrogenesis and recovery of neurological function by erythropoietin after neonatal hypoxic/ischemic brain injury. *Stroke.*41(5):1032-1037.
84. Wu YW, Bauer LA, Ballard RA, et al. Erythropoietin for neuroprotection in neonatal encephalopathy: safety and pharmacokinetics. *Pediatrics.* 2012;130(4):683-691.
85. Statler PA, McPherson RJ, Bauer LA, Kellert BA, Juul SE. Pharmacokinetics of high-dose recombinant erythropoietin in plasma and brain of neonatal rats. *Pediatric research.* 2007;61(6):671-675.
86. Weber A, Dzietko M, Berns M, et al. Neuronal damage after moderate hypoxia and erythropoietin. *Neurobiol Dis.* 2005;20(2):594-600.
87. Plateel M, Teissier E, Cecchelli R. Hypoxia dramatically increases the nonspecific transport of blood-borne proteins to the brain. *J Neurochem.* 1997;68(2):874-877.
88. Brines ML, Ghezzi P, Keenan S, et al. Erythropoietin crosses the blood-brain barrier to protect against experimental brain injury. *Proc Natl Acad Sci U S A.* 2000;97(19):10526-10531.
89. Juul SE, Harcum J, Li Y, Christensen RD. Erythropoietin is present in the cerebrospinal fluid of neonates. *J Pediatr.* 1997;130(3):428-430.

90. Juul SE, McPherson RJ, Farrell FX, Jolliffe L, Ness DJ, Gleason CA. Erytropoietin concentrations in cerebrospinal fluid of nonhuman primates and fetal sheep following high-dose recombinant erythropoietin. *Biol Neonate.* 2004;85(2):138-144.
91. Xenocostas A, Cheung WK, Farrell F, et al. The pharmacokinetics of erythropoietin in the cerebrospinal fluid after intravenous administration of recombinant human erythropoietin. *Eur J Clin Pharmacol.* 2005;61(3):189-195.
92. Economic costs associated with mental retardation, cerebral palsy, hearing loss, and vision impairment--United States, 2003. *MMWR Morb Mortal Wkly Rep.* 2004;53(3):57-59.
93. Azzopardi D, Strohm B, Linsell L, et al. Implementation and Conduct of Therapeutic Hypothermia for Perinatal Asphyxial Encephalopathy in the UK - Analysis of National Data. *PLoS One.* 2012;7(6):e38504.
94. Malla RR, Asimi R, Teli MA, Shaheen F, Bhat MA. Erythropoietin monotherapy in perinatal asphyxia with moderate to severe encephalopathy: a randomized placebo-controlled trial. *J Perinatol.* 2017;37(5):596-601.
95. Ismail N, Ikizler TA. Erythropoietin-induced hypertension. *J Med Liban.* 1997;45(1):25-30.
96. Wolf RF, Gilmore LS, Friese P, Downs T, Burstein SA, Dale GL. Erythropoietin potentiates thrombus development in a canine arterio-venous shunt model. *Thromb Haemost.* 1997;77(5):1020-1024.
97. Wolf RF, Peng J, Friese P, Gilmore LS, Burstein SA, Dale GL. Erythropoietin administration increases production and reactivity of platelets in dogs. *Thromb Haemost.* 1997;78(6):1505-1509.
98. Casadevall N. Pure red cell aplasia and anti-erythropoietin antibodies in patients treated with epoetin. *Nephrol Dial Transplant.* 2003;18 Suppl 8:viii37-41.
99. Ehrenreich H, Weissenborn K, Prange H, et al. Recombinant human erythropoietin in the treatment of acute ischemic stroke. *Stroke.* 2009;40(12):e647-656.
100. Juul S. Erythropoietin in anemia of prematurity. *J Matern Fetal Neonatal Med.* 2012;25(Suppl 5):80-84.
101. Ohlsson A, Aher SM. Early erythropoietin for preventing red blood cell transfusion in preterm and/or low birth weight infants. *Cochrane Database Syst Rev.* 2006;3:CD004863.
102. Doege C, Pritsch M, Fruhwald MC, Bauer J. An association between infantile haemangiomas and erythropoietin treatment in preterm infants. *Arch Dis Child Fetal Neonatal Ed.* 2012;97(1):F45-49.
103. Fauchere JC, Koller BM, Tschopp A, et al. Safety of Early High-Dose Recombinant Erythropoietin for Neuroprotection in Very Preterm Infants. *J Pediatr.* 2015;167(1):52-57 e51-53.
104. Juul SE, Mayock DE, Comstock BA, Heagerty PJ. Neuroprotective potential of erythropoietin in neonates; design of a randomized trial. *Matern Health Neonatol Perinatol.* 2015;1:27.
105. Baserga MC, Beachy JC, Roberts JK, et al. Darbepoetin administration to neonates undergoing cooling for encephalopathy: a safety and pharmacokinetic trial. *Pediatric research.* 2015;78(3):315-322.
106. Juul SE, Mayock DE, Comstock BA, Heagerty PJ. Neuroprotective potential of erythropoietin in neonates; design of a randomized trial. *Maternal Health, Neonatology and Perinatology* 2015;1:27.
107. Juul SE, McPherson RJ, Bauer LA, Ledbetter KJ, Gleason CA, Mayock DE. A phase I/II trial of high-dose erythropoietin in extremely low birth weight infants: pharmacokinetics and safety. *Pediatrics.* 2008;122(2):383-391.

108. Fauchere JC, Dame C, Vonthein R, et al. An approach to using recombinant erythropoietin for neuroprotection in very preterm infants. *Pediatrics.* 2008;122(2):375-382.
109. Andropoulis D. Erythropoetin Neuroprotection for Neonatal Cardiac Surgery. NCT00513240. Available at. Accessed ClinicalTrials.gov, February 2, 2012.
110. Benders MJ, van der Aa NE, Roks M, et al. Feasibility and safety of erythropoietin for neuroprotection after perinatal arterial ischemic stroke. *J Pediatr.* 2014;164(3):481-486 e481-482.
111. Messier AM, Ohls RK. Neuroprotective effects of erythropoiesis-stimulating agents in term and preterm neonates. *Current Opinion in Pediatrics.* 2014;26(2):139-145.
112. Ohls RK, Kamath-Rayne BD, Christensen RD, et al. Cognitive outcomes of preterm infants randomized to darbepoetin, erythropoietin, or placebo. *Pediatrics.* 2014;133(6):1023-1030.
113. Natalucci G, Latal B, Koller B, et al. Effect of Early Prophylactic High-Dose Recombinant Human Erythropoietin in Very Preterm Infants on Neurodevelopmental Outcome at 2 Years: A Randomized Clinical Trial. *JAMA.* 2016;315(19):2079-2085.
114. Widness JA, Veng-Pedersen P, Peters C, Pereira LM, Schmidt RL, Lowe LS. Erythropoietin pharmacokinetics in premature infants: developmental, nonlinearity, and treatment effects. *J Appl Physiol.* 1996;80(1):140-148.
115. Brown MS, Jones MA, Ohls RK, Christensen RD. Single-dose pharmacokinetics of recombinant human erythropoietin in preterm infants after intravenous and subcutaneous administration. *J Pediatr.* 1993;122(4):655-657.
116. Inder TE, Hunt RW, Morley CJ, et al. Randomized trial of systemic hypothermia selectively protects the cortex on MRI in term hypoxic-ischemic encephalopathy. *J Pediatr.* 2004;145(6):835-837.
117. Rutherford M, Ramenghi LA, Edwards AD, et al. Assessment of brain tissue injury after moderate hypothermia in neonates with hypoxic-ischaemic encephalopathy: a nested substudy of a randomised controlled trial. *Lancet Neurol.* 2010;9(1):39-45.
118. Bonifacio SL, Glass HC, Vanderpluym J, et al. Perinatal Events and Early Magnetic Resonance Imaging in Therapeutic Hypothermia. *J Pediatr.* 2010.
119. Bednarek N, Mathur A, Inder T, Wilkinson J, Neil J, Shimony J. Impact of therapeutic hypothermia on MRI diffusion changes in neonatal encephalopathy. *Neurology.* 2012;78(18):1420-1427.
120. Cheong JL, Coleman L, Hunt RW, et al. Prognostic utility of magnetic resonance imaging in neonatal hypoxic-ischemic encephalopathy: substudy of a randomized trial. *Arch Pediatr Adolesc Med.* 2012;166(7):634-640.
121. Msall ME. Measuring functional skills in preschool children at risk for neurodevelopmental disabilities. *Ment Retard Dev Disabil Res Rev.* 2005;11(3):263-273.
122. Msall M, Tremont MR, Ottenbacher KJ. Functional assessment of preschool children: optimizing developmental and family supports in early intervention. *Infants Young Child.* 2001;14:16-66.
123. Shellhaas RA, Chang T, Tsuchida T, et al. The American Clinical Neurophysiology Society's Guideline on Continuous Electroencephalography Monitoring in Neonates. *J Clin Neurophysiol.* 2011;28(6):611-617.
124. Ehrenreich H, Kastner A, Weissenborn K, et al. Circulating damage marker profiles support a neuroprotective effect of erythropoietin in ischemic stroke patients. *Mol Med.* 2011.
125. Douglas-Escobar M, Yang C, Bennett J, et al. A pilot study of novel biomarkers in neonates with hypoxic-ischemic encephalopathy. *Pediatric research.* 2010;68(6):531-536.

126. Florio P, Abella R, Marinoni E, et al. Biochemical markers of perinatal brain damage. *Front Biosci (Schol Ed).* 2010;2:47-72.
127. Douglas-Escobar M, Weiss MD. Biomarkers of brain injury in the premature infant. *Front Neurol.* 2012;3:185.
128. Ment LR, Bada HS, Barnes P, et al. Practice parameter: neuroimaging of the neonate: report of the Quality Standards Subcommittee of the American Academy of Neurology and the Practice Committee of the Child Neurology Society. *Neurology.* 2002;58(12):1726-1738.
129. Kuban KC, Allred EN, O'Shea M, Paneth N, Pagano M, Leviton A. An algorithm for identifying and classifying cerebral palsy in young children. *J Pediatr.* 2008;153(4):466-472.
130. Kuban KC, O'Shea M, Allred E, et al. Video and CD-ROM as a training tool for performing neurologic examinations of 1-year-old children in a multicenter epidemiologic study. *J Child Neurol.* 2005;20(10):829-831.
131. Palisano R, Rosenbaum P, Walter S, Russell D, Wood E, Galuppi B. Development and reliability of a system to classify gross motor function in children with cerebral palsy. *Dev Med Child Neurol.* 1997;39(4):214-223.
132. Bayley N. Bayley scales of infant and toddler development. San Antonio, TX: The Psychological Corporation; 2006.
133. Kuban KC, O'Shea M, Allred E, et al. Video and CD-ROM as a training tool for performing neurologic examinations of 1-year-old children in a multicenter epidemiologic study. *J Child Neurol.* 2005;20(10):829-831.
134. O'Shea TM, Allred EN, Dammann O, et al. The ELGAN study of the brain and related disorders in extremely low gestational age newborns. *Early human development.* 2009;85(11):719-725.
135. Kuban KC, Allred EN, O'Shea M, Paneth N, Pagano M, Leviton A. An algorithm for identifying and classifying cerebral palsy in young children. *The Journal of Pediatrics.* 2008;153(4):466-472.
136. Anderson PJ, De Luca CR, Hutchinson E, Roberts G, Doyle LW. Underestimation of developmental delay by the new Bayley-III Scale. *Arch Pediatr Adolesc Med.* 2010;164(4):352-356.
137. Flynn JT. Neonatal Hypertension: Diagnosis and management. *Pediatr Nephrol.* 2000;14:332-341.
138. Dionne JM, Abitbol CL, Flynn JT. Hypertension in infancy: diagnosis, management and outcome. *Pediatr Nephrol.* 2011.
139. Vohr BR, Stephens BE, Higgins RD, et al. Are Outcomes of Extremely Preterm Infants Improving? Impact of Bayley Assessment on Outcomes. *J Pediatr.* 2012.
140. Moore T, Johnson S, Haider S, Hennessy E, Marlow N. Relationship between Test Scores Using the Second and Third Editions of the Bayley Scales in Extremely Preterm Children. *J Pediatr.* 2012;160(4):553-558.
141. Chalak LF, Dupont TL, Sanchez PJ, et al. Neurodevelopmental outcomes after hypothermia therapy in the era of Bayley-III. *J Perinatol.* 2014.
142. Walsh MC, Hibbs AM, Martin CR, et al. Two-year neurodevelopmental outcomes of ventilated preterm infants treated with inhaled nitric oxide. *J Pediatr.* 2010;156(4):556-561 e551.
143. Achenbach TM, Rescorla LA. Manual for the ASEBA Preschool Forms & Profiles. *Child Behavior Checklist for Ages 1.5 - 5*. Burlington, VT: Research Center for Children, Youth & Families; 2000.
144. Wu YW, Escobar GJ, Grether JK, Croen LA, Greene JD, Newman TB. Chorioamnionitis and cerebral palsy in term and near-term infants. *JAMA.* 2003;290(20):2677-2684.

145. Wu YW, Croen LA, Torres AR, Van De Water J, Grether JK, Hsu NN. Interleukin-6 genotype and risk for cerebral palsy in term and near-term infants. *Ann Neurol.* 2009;66(5):663-670.
146. Yang L, Sameshima H, Ikeda T, Ikenoue T. Lipopolysaccharide administration enhances hypoxic-ischemic brain damage in newborn rats. *J Obstet Gynaecol Res.* 2004;30(2):142-147.
147. Badawi N, Kurinczuk JJ, Keogh JM, et al. Intrapartum risk factors for newborn encephalopathy: the Western Australian case-control study [see comments]. *BMJ.* 1998;317(7172):1554-1558.
148. Diggle PJ, Heagerty PJ, Liang KY, Zeger SL. *Analysis of Longitudinal Data.* Second Edition ed: Oxford University Press; 2002.
149. Traudt CM, Juul SE. Erythropoietin as a neuroprotectant for neonatal brain injury: animal models. *Methods Mol Biol.* 2013;982:113-126.
150. Cheong JL, Coleman L, Hunt RW, et al. Prognostic utility of magnetic resonance imaging in neonatal hypoxic-ischemic encephalopathy: substudy of a randomized trial. *Arch Pediatr Adolesc Med.* 2012;166(7):634-640.
151. Sahu R, Pannu H, Yu R, Shete S, Bricker JT, Gupta-Malhotra M. Systemic hypertension requiring treatment in the neonatal intensive care unit. *J Pediatr.* 2013;163(1):84-88.
152. Friedman AL, Hustead VA. Hypertension in babies following discharge from a neonatal intensive care unit. A 3-year follow-up. *Pediatr Nephrol.* 1987;1(1):30-34.
153. Seliem WA, Falk MC, Shadbolt B, Kent AL. Antenatal and postnatal risk factors for neonatal hypertension and infant follow-up. *Pediatr Nephrol.* 2007;22(12):2081-2087.
154. Vlug RD, Lopriore E, Janssen M, Middeldorp JM, Rath ME, Smits-Wintjens VE. Thrombocytopenia in neonates with polycythemia: incidence, risk factors and clinical outcome. *Expert Rev Hematol.* 2015;8(1):123-129.
155. Zhou WH, Cheng GQ, Shao XM, et al. Selective head cooling with mild systemic hypothermia after neonatal hypoxic-ischemic encephalopathy: a multicenter randomized controlled trial in China. *J Pediatr.* 2010;157(3):367-372, 372 e361-363.
156. Gunn AJ, Gluckman PD, Gunn TR. Selective head cooling in newborn infants after perinatal asphyxia: a safety study. *Pediatrics.* 1998;102(4 Pt 1):885-892.
157. Herlenius E, Kuhn P. Sudden unexpected postnatal collapse of newborn infants: a review of cases, definitions, risks, and preventive measures. *Transl Stroke Res.* 2013;4(2):236-247.
158. Tagin MA, Woolcott CG, Vincer MJ, Whyte RK, Stinson DA. Hypothermia for neonatal hypoxic ischemic encephalopathy: an updated systematic review and meta-analysis. *Arch Pediatr Adolesc Med.* 2012;166(6):558-566.
159. Edwards AD, Brocklehurst P, Gunn AJ, et al. Neurological outcomes at 18 months of age after moderate hypothermia for perinatal hypoxic ischaemic encephalopathy: synthesis and meta-analysis of trial data. *BMJ.* 2010;340:c363.

14 Appendices

14.1 ACRONYMS

AE	Adverse Event
aEEG	Amplitude-integrated EEG
AIMS	Alberta Infant Motor Scale
ANCOVA	Analysis of Covariance
AUC	Area Under the Curve
Bayley III	Bayley Scales of Infant and Toddler Development, 3rd Edition
BDNF	Brain-Derived Neurotrophic Factor
BEAM	Beneficial Effects of Antenatal Magnesium Sulfate
BGT	Basal Ganglia and Thalamus
CBCL	Child Behavior Checklist
CCC	Clinical Coordinating Center
cEEG	Continuous EEG
CNS	Central Nervous System
CONSORT	Consolidated Standards of Reporting Trials
CP	Cerebral Palsy
CPAP	Continuous Positive Airway Pressure
CRF	Case Report Form
CSF	Cerebrospinal Fluid
DCC	Data Coordinating Center
DIC	Disseminated Intravascular Coagulation
DSMB	Data Safety Monitoring Board
DSMP	Data Safety Monitoring Plan
DTI	Diffusion Tensor Imaging
ECMO	Extracorporeal Membrane Oxygenation
EEG	Electroencephalogram
ELGAN	Extremely Low Gestational Age Newborns
Epo	Erythropoietin
Epo-R	Erythropoietin Receptor
ERK1	Extracellular Signal-Related Kinase

ETT	Endotracheal Tube
FDA	Food and Drug Administration
GDNF	Glial cell derived neurotrophic factor
GFAP	Glial Fibrillary Acidic Protein
GMFCS	Gross Motor Function Classification System
HCT	Hematocrit
HIE	Hypoxic-Ischemic Encephalopathy
HIPAA	Health Insurance Portability and Accountability Act
HT	Hypothermia
HUS	Head Ultrasound
ICE Trial	Infant Cooling Evaluation Trial
IL	Interleukin
IND	Investigational New Drug
IP	Intraperitoneal
IRB	Institutional Review Board
IV	Intravenous
JAK2	Janus Kinase 2
MAR	Missing at Random
mITT	Modified Intent to Treat
MCAR	Missing Completely at Random
MRI	Magnetic Resonance Imaging
MRS	Magnetic Resonance Spectroscopy
MSD	Meso Scale Discovery
NDI	Neurodevelopmental Impairment
NEAT	Neonatal Erythropoietin in Asphyxiated Term Newborns
NEATO	Neonatal Erythropoietin and Therapeutic Hypothermia Outcomes in Newborn Brain Injury
NINDS	National Institute of Neurological Disorders and Stroke
NS	Normal Saline
NSE	Neuron-Specific Encolase
OT	Occupational Therapy

PENUT	Preterm Erythropoietin Neuroprotection Trial
PI	Principal Investigator
PI3K	Phosphatidylinositol-3 kinase
PT	Physical Therapy
QA	Quality Assurance
SAE	Serious Adverse Event
SC	Subcutaneous
SD	Standard Deviation
SOP	Standard Operating Procedure
ST	Speech Therapy
STAT5	Signal Transducer and Activator of Transcription 5
TNF-α	Tumor Necrosis Factor-α
TOLSURF	Trial of Late Surfactant
UCH-L1	Ubiquitin C-terminal Hydrolase-L1
VEGF	Vascular Endothelial Growth Factor
WIDEA	Warner Initial Developmental Evaluation of Adaptive and Functional Skills

Changes from Version 2.7 -> Version 2.8

Administrative Changes

- Changes to order of Neuroimaging Core personnel. Jessica Wisnowski, PhD has moved into the role of Neuroimaging Core PI. Amit Mathur, MD has moved institutions.
- Clarification that CVs provided to the CCC should be signed and dated.

Procedure Updates

- Addition of the Extended Contact phone calls every 6 months from 2.5 through 8 years of age.
