# Supplementary material for: Association of High-Dose Erythropoietin With Circulating Biomarkers and Neurodevelopmental Outcomes Among Neonates With Hypoxic Ischemic Encephalopathy: A Secondary Analysis of the HEAL Randomized Clinical Trial
Source: JAMA Netw Open. 2023 Jul 7;6(7):e2322131. doi: 10.1001/jamanetworkopen.2023.22131 (PMC10329214; doi:10.1001/jamanetworkopen.2023.22131)
Supplement: Supplement 2. — eTable 1. Univariate Relative Risks (RRs) for the Association Between Each Biomarker Measure and the Primary Outcome of Death or Neurodevelopmental Impairment eFigure 1. Selection of Biomarkers by LASSO Within Each Time Point eFigure 2. ROC Curves eTable 2. Predictive Ability of Biomarker Data Sets eTable 3. AUC From Multivariable Models of Biomarker Concentrations and Their Association With Death or NDI eTable 4. Adjusted P Values for Comparisons of AUC Between Multivariable Models of Biomarker Concentrations and Their Association With Death or NDI eTable 5. Median Biomarker Values by Treatment Group and Rank-Sum Differences Between Groups [file jamanetwopen-e2322131-s002.pdf]

Supplemental Online Content

Juul SE, Voldal E, Comstock BA, et al; HEAL consortium. Association of high-dose erythropoietin with circulating biomarkers and neurodevelopmental outcomes among neonates with hypoxic ischemic encephalopathy: a secondary analysis of the HEAL randomized clinical trial. *JAMA Netw Open.* 2023;6(7):e2322131. doi:10.1001/jamanetworkopen.2023.22131

eTable 1. Univariate Relative Risks (RRs) for the Association Between Each Biomarker Measure and the Primary Outcome of Death or Neurodevelopmental Impairment

eFigure 1. Selection of Biomarkers by LASSO Within Each Time Point

eFigure 2. ROC Curves

eTable 2. Predictive Ability of Biomarker Data Sets

eTable 3. AUC From Multivariable Models of Biomarker Concentrations and Their Association With Death or NDI

eTable 4. Adjusted *P* Values for Comparisons of AUC Between Multivariable Models of Biomarker Concentrations and Their Association With Death or NDI

eTable 5. Median Biomarker Values by Treatment Group and Rank-Sum Differences Between Groups

This supplemental material has been provided by the authors to give readers additional information about their work.

© 2023 Juul SE et al. *JAMA Network Open.*

eTable 1. Univariate Relative Risks (RRs) for the Association Between Each Biomarker Measure and the Primary Outcome of Death or Neurodevelopmental Impairment

Biomarker measures were log2-transformed and winsorized. P-values marked with an asterisk remain <.05 after adjusting for multiple comparisons.

Biomarker	Timepoint	No death/NDI median (IQR)	Death/NDI median (IQR)	RR (95% CI)	Unadjusted p-value
**Anti-Inflammatory**					
IL-1RA	Baseline	5240.4 (12222.9)	8517.7 (19461.3)	1.07 (0.99, 1.16)	0.08
	Day 2	2420.4 (4008.2)	2273.4 (2933.7)	1.05 (0.95, 1.16)	0.31
	Day 4	2396.9 (3051.7)	2690.2 (4322.5)	1.1 (1, 1.22)	0.05
**IL-10**	**Baseline**	**43.3 (73.9)**	**94.2 (510.8)**	**1.13 (1.06, 1.21)**	**<0.001***
	Day 2	25.0 (26.2)	31.9 (81.3)	1.15 (1.05, 1.26)	0.002
	**Day 4**	**12.3 (10.1)**	**15.54(16.4)**	**1.27 (1.11, 1.44)**	**<0.001***
IL-13	Baseline	0.8 (4.2)	2.0 (8.5)	1.06 (1, 1.11)	0.04
	Day 2	0.8 (3.5)	2.2 (9.2)	1.07 (1.02, 1.13)	0.005
	Day 4	0.6 (3.0)	1.5 (8.8)	1.08 (1.03, 1.14)	0.002
IL-33	Baseline	5.1 (3.8)	5.9 (4.1)	1.17 (1.01, 1.36)	0.03
	Day 2	4.7 (2.4)	5.1 (3.6)	1.15 (0.96, 1.38)	0.13
	Day 4	5.2 (2.8)	5.4 (3.3)	1.07 (0.9, 1.28)	0.43
**Pro-inflammatory**					
**IL-8**	**Baseline**	**102.7 (140.4)**	**199.3 (442.5)**	**1.16 (1.07, 1.26)**	**<0.001***
	Day 2	89.1 (157.2)	108.44 (242.1)	1.18 (1.06, 1.31)	0.003
	**Day 4**	**65.8 (68.1)**	**93.64 (124.5)**	**1.24 (1.12, 1.37)**	**<0.001***
C5a	Baseline	24074.9 (15597.2)	24703.8 (18545.6)	0.84 (0.72, 0.99)	0.03
	Day 2	24720.9 (15214.5)	22860.6 (15735.6)	0.82 (0.7, 0.97)	0.02
	Day 4	18700.1 (8102.2)	18615.5 (13468.7)	0.89 (0.73, 1.09)	0.26
**Fractalkine**	Baseline	96.7 (73.3)	132.7 (154.7)	1.28 (1.11, 1.48)	0.001
	Day 2	89.8 (63)	107 (131.8)	1.27 (1.07, 1.51)	0.006
	**Day 4**	**111.6 (66.3)**	**132.5 (86.7)**	**1.51 (1.22, 1.88)**	**<0.001***
ICAM-1	Baseline	472688 (263565)	567677 (369987)	1.16 (0.94, 1.41)	0.16

© 2023 Juul SE et al. *JAMA Network Open.*

	Day 2	559796 (248581)	601280 (314263)	1.14 (0.9, 1.45)	0.27
	Day 4	624453 (212340)	655784 (224818)	1.12 (0.86, 1.46)	0.41
IFN-g	Baseline	4.03 (5.06)	5.34 (8.79)	1.1 (1, 1.22)	0.04
	Day 2	3.91 (4.11)	4.51 (11.5)	1.09 (0.99, 1.2)	0.08
	Day 4	4.05 (5.09)	4.95 (11.33)	1.16 (1.05, 1.28)	0.004
**IL-1b**	Baseline	0.47 (0.55)	0.7 (1.71)	1.11 (1.01, 1.22)	0.03
	Day 2	0.49 (0.43)	0.57 (1.27)	1.17 (1.05, 1.29)	0.004
	**Day 4**	**0.49 (0.51)**	**0.66 (1.16)**	**1.22 (1.1, 1.36)**	**<0.001***
IL-6	Baseline	30.8 (73.7)	62.2 (220.7)	1.11 (1.04, 1.18)	0.001
	Day 2	42.3 (52.1)	40.5 (80.0)	1.1 (1.01, 1.2)	0.03
	Day 4	12.65 (21.4)	13.22 (23.8)	1.07 (0.98, 1.16)	0.13
**IL-12p70**	Baseline	0.5 (0.4)	0.76 (0.8)	1.15 (1.05, 1.27)	0.003
	Day 2	0.57 (0.5)	0.77 (1.8)	1.1 (1, 1.21)	0.05
	**Day 4**	**0.59 (0.6)**	**0.89 (0.93)**	**1.25 (1.1, 1.41)**	**<0.001***
**IL-17A**	Baseline	1.13 (1.05)	1.48 (2.07)	1.17 (1.05, 1.3)	0.006
	Day 2	1.21 (1.03)	1.68 (2.55)	1.18 (1.06, 1.31)	0.002
	**Day 4**	**1.39 (1.05)**	**2.03 (3.13)**	**1.25 (1.12, 1.4)**	**<0.001***
MCP-1	Baseline	267.5 (327.6)	364.1 (479.5)	1.13 (1.03, 1.23)	0.009
	Day 2	754.5 (640.5)	626.6 (776.7)	1.06 (0.94, 1.19)	0.33
	Day 4	572.2 (428)	677.8 (552.9)	1.21 (1.05, 1.39)	0.007
MIP-1a	Baseline	17.9 (19.8)	19.4 (21.4)	1.04 (0.97, 1.11)	0.30
	Day 2	26.8 (18.8)	27.4 (22.8)	1.12 (1.01, 1.25)	0.03
	Day 4	20.9 (18.6)	23.8 (22.8)	1.03 (0.96, 1.11)	0.41
MIP-1b	Baseline	53 (35)	61.7 (47.4)	1.02 (0.85, 1.22)	0.83
	Day 2	75.5 (41.5)	61.8 (43)	0.85 (0.71, 1.03)	0.10
	Day 4	61.7 (36)	54.74(39.9)	0.84 (0.69, 1.01)	0.06
VCAM-1	Baseline	4760323 (1459255)	4388853(1868927)	0.85 (0.63, 1.15)	0.30
	Day 2	2328579 (1459946)	2651081(1938964)	1.23 (1, 1.51)	0.05
	Day 4	3510876 (2393847)	4047734(1931618)	1.19 (0.91, 1.57)	0.21
TNF-a	Baseline	24.9 (15.5)	27 (17.3)	1.06 (0.89, 1.27)	0.50
	Day 2	34.3 (16.6)	28.7 (19.7)	0.97 (0.79, 1.19)	0.78
	Day 4	31.9 (17.4)	27.1 (15.5)	0.83 (0.65, 1.06)	0.13
**Brain specific**					

© 2023 Juul SE et al. *JAMA Network Open.*

GFAP	Baseline	828.2 (712.3)	1066.3 (1026.9)	1.08 (0.96, 1.23)	0.21
	Day 2	637.2 (448.3)	1145 (2033)	1.21 (1.08, 1.36)	0.001
	Day 4	1141.6 (886)	1743 (3023)	1.14 (1.04, 1.24)	0.005
NSE*	Baseline	44232 (25619)	48512 (35595)	1.17 (0.92, 1.49)	0.19
	Day 2	42488 (24194)	51830.5 (26324.8)	1.34 (1.07, 1.67)	0.01
	Day 4	29719 (18785)	39192.4 (27063.2)	1.25 (1.01, 1.53)	0.04
**S100b**	**Baseline**	**1285.5 (1974.6)**	**3060.6 (7528.4)**	**1.12 (1.05, 1.2)**	**0.001***
	Day 2	813.1 (1268.4)	1670.3 (2602.8)	1.18 (1.07, 1.3)	0.001
	Day 4	978.4 (837.6)	1284.1 (1416.7)	1.19 (1.06, 1.33)	0.003
**UCHL1**	**Baseline**	**1956.1 (1353.5)**	**2500.6 (3246.9)**	**1.15 (1.06, 1.25)**	**0.001***
	Day 2	1673.8 (735.2)	2018.1 (1158.8)	1.11 (0.99, 1.24)	0.07
	Day 4	1486.4 (832)	1993.8 (1014.6)	1.31 (1.05, 1.64)	0.02
**Tau**	Baseline	173.5 (129.5)	224.8 (328.4)	1.08 (0.95, 1.22)	0.26
	**Day 2**	**121.7 (99.0)**	**275.6 (563.1)**	**1.32 (1.17, 1.49)**	**<0.001***
	**Day 4**	**126.2 (107.7)**	**375.6 (1352.3)**	**1.28 (1.17, 1.4)**	**<0.001***
**Other Growth Factors**					
**BDNF**	Baseline	247.6 (380.4)	248.2 (388.1)	1 (0.92, 1.09)	0.98
	Day 2	147.7 (234.8)	142.6 (242.1)	1.03 (0.94, 1.14)	0.54
	Day 4	305.8 (597.9)	311.5 (541.1)	1.01 (0.93, 1.1)	0.80
**Epo**	Baseline	26.6 (39.6)	44 (272.9)	1.05 (1, 1.1)	0.03
**NCAM-1**	Baseline	307426.9 (53158.2)	311411 (87537)	0.69 (0.44, 1.09)	0.11
	Day 2	302430.7 (69229.1)	300049.4 (84018)	0.71 (0.42, 1.19)	0.19
	Day 4	291749.6 (61037)	281575.2 (74692)	0.54 (0.34, 0.87)	0.01
**NGB**	Baseline	10.0 (8.7)	12.2 (9.8)	1.17 (1.04, 1.33)	0.01
	Day 2	9.7 (6.5)	10.4 (8.1)	1.24 (1.04, 1.47)	0.01
	Day 4	9.7 (5.8)	10.7 (6.5)	1.26 (1.05, 1.52)	0.02
**VEGF**	Baseline	32.8 (19.3)	25.6 (20)	0.88 (0.76, 1.02)	0.09
	Day 2	40.5 (14.8)	34.6 (26.8)	0.88 (0.76, 1.01)	0.08
	Day 4	44.2 (20.4)	45.6 (25.9)	1.05 (0.85, 1.3)	0.65

Abbreviations: BDNF: brain-derived neurotrophic factor; C5a: complement component 5a; CI: confidence interval; EPO: erythropoietin; GFAP: glial fibrillary acidic protein; ICAM-1: intercellular adhesion molecule 1; IFN-g: Interferon-g; IL: Interleukin; IL-1RA: IL-1 receptor antagonist; MCP-1: monocyte chemoattractant protein-1; IQR: Interquartile range; MIP: macrophage inflammatory protein; NCAM-1: neural cell adhesion molecule; NGB: neuroregulin-1-b-1; NSE: neuron-specific enolase; RR: Relative risk; s100b: S100 calcium-binding protein B; TNF-a: tumor necrosis factor-a; UCHL1: ubiquitin carboxy-terminal hydrolase-L1; VCAM-1: vascular cell adhesion molecule 1; VEGF: vascular endothelial growth factor.

© 2023 Juul SE et al. *JAMA Network Open.*

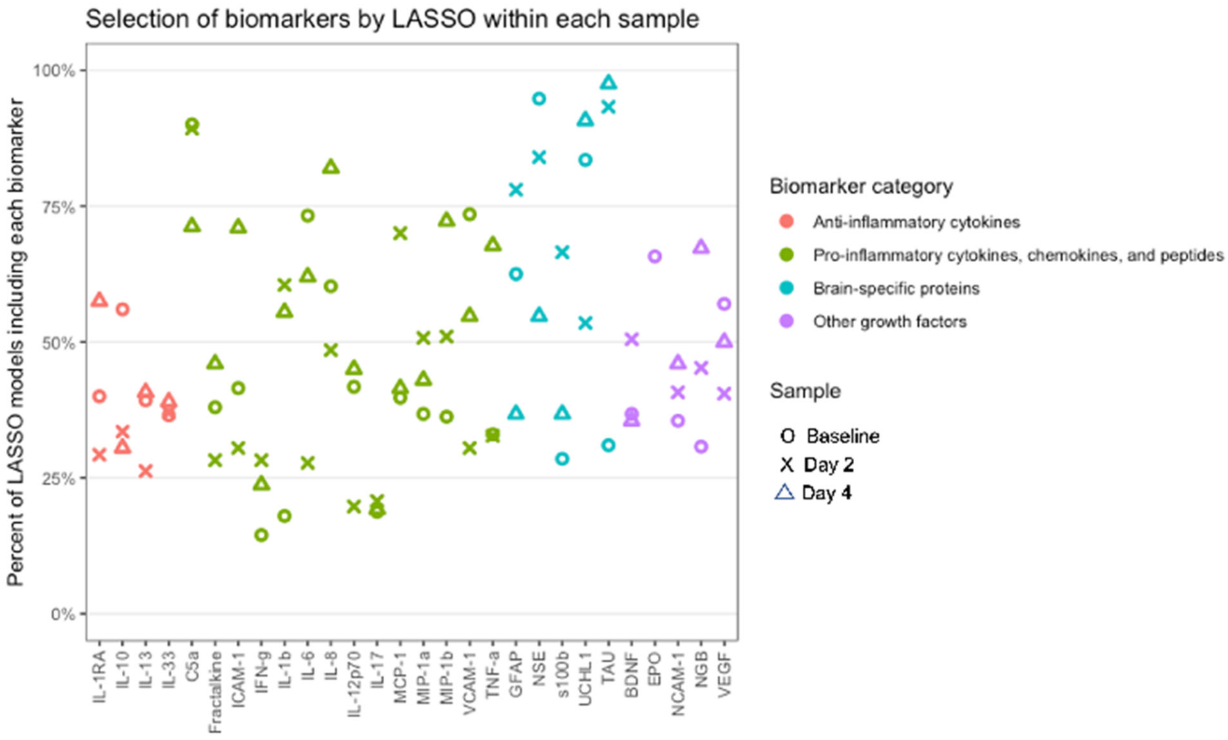

eFigure 1. Selection of Biomarkers by LASSO Within Each Time Point

Biomarkers within the pro-inflammatory cytokines/chemokines/peptides and brain-specific protein categories were most often selected by LASSO. Note: p-values are not indicative of effect size.

© 2023 Juul SE et al. *JAMA Network Open.*

eFigure 2. ROC Curves

ROC curves are compared for baseline clinical data (BCD), BCD with biomarker concentrations at baseline, day 2, and day 4 timepoints, and finally, BCD with a curated set of biomarkers. The addition of biomarker data from baseline, day 4, or a curated set to BCD improved area under the curve (AUC) scores compared to BCD alone.

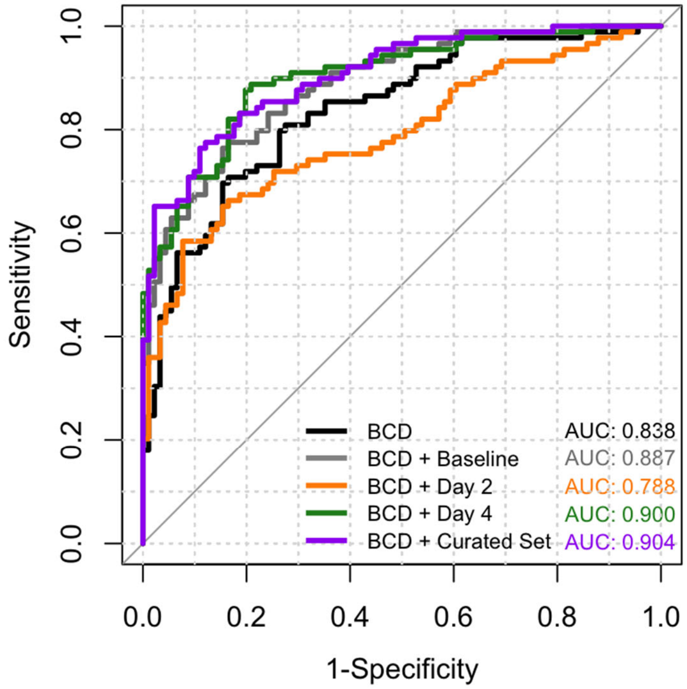

© 2023 Juul SE et al. *JAMA Network Open.*

eTable 2. Predictive Ability of Biomarker Data Sets

Biomarker	RR (95% CI) from model with clinical biomarkers	RR (95% CI) from model with experimental biomarkers	RR (95% CI) from full model
Female Sex	0.93 (0.70, 1.23)		1.00 (0.75, 1.35)
5-minute Apgar	0.96 (0.89, 1.03)		0.99 (0.92, 1.07)
Continue resuscitation at 10 min[a]	1.47 (0.68, 3.19)		1.36 (0.64, 2.9)
Worst base deficit[b]	0.99 (0.96, 1.02)		1.01 (0.98, 1.04)
Lowest pH[b]	0.62 (0.21,1.89)		0.67 (0.26, 1.78)
HIE Severity	1.00 (0.75-1.32)	1.34 (1-1.81)	1.28 (0.93-1.78)
C5a (baseline)		0.82 (0.71, 0.94)	0.82 (0.71, 0.94)
IL-6 (baseline)		1.08 (1.01, 1.16)	1.08 (1.0, 1.16)
NSE (baseline)		1.19 (1.02, 1.4)	1.21 (1.01, 1.45)
IL-8 (day 4)		1.09 (0.96, 1.23)	1.09 (0.96, 1.24)
Tau (day 4)		1.16 (1.06, 1.28)	1.16 (1.04, 1.28)
UCHL1 (day 4)		1.14 (0.97, 1.35)	1.15 (0.96, 1.37)

[a] Ongoing resuscitation with chest compressions, mechanical ventilation, or both was warranted at 10 minutes of age.
[b] Shown is the lowest pH or worst base deficit among cord arterial, cord venous, or arterial blood gas samples obtained before 60 minutes of age.
Abbreviations: C5a: complement component 5a; CI: confidence interval; IL: Interleukin; NSE: neuron-specific enolase; RR: relative risk; UCHL1: ubiquitin carboxy-terminal hydrolase-L1.

© 2023 Juul SE et al. *JAMA Network Open.*

eTable 3. AUC From Multivariable Models of Biomarker Concentrations and Their Association With Death or NDI

Adjusted for HIE severity, treatment assignment, and site, pooled over multiply imputed data.

**Model**	**Data**	**AUC (95% CI)**
Baseline clinical data	HIE severity, treatment assignment, site, baseline clinical variables[a]	0.73 (0.70-0.75)
Top 5 biomarkers at baseline	Baseline clinical data + baseline biomarkers[b]	0.77 (0.75-0.80)
Top 5 biomarkers at day 2	Baseline clinical data + day 2 biomarkers[b]	0.76 (0.73-0.78)
Top 5 biomarkers at day 4	Baseline clinical data + day 4 biomarkers[b]	0.79 (0.77-0.81)
Curated set of biomarkers	Baseline clinical data + curated set of biomarkers from baseline and day 4[c]	0.78 (0.75-0.80)

[a]Baseline clinical data includes infant sex, 5-minute Apgar score, continued resuscitation at 10 minutes, lowest pH, and worst base deficit.
[b] Five most-selected biomarkers at each timepoint after bootstrapped LASSO.
[c] Curated set of biomarkers includes concentrations of C5a, IL-6, and NSE at baseline as well as IL-8 and Tau at day 4.

© 2023 Juul SE et al. *JAMA Network Open.*

eTable 4. Adjusted *P* Values for Comparisons of AUC Between Multivariable Models of Biomarker Concentrations and Their Association With Death or NDI

Adjusted for HIE severity, treatment assignment, and site, pooled over multiply imputed data.

	Baseline clinical data[a]	Baseline clinical data[a] + Baseline biomarkers[b]	Baseline clinical data[a] + Day 2 biomarkers[b]	Baseline clinical data[a] + Day 4 biomarkers[b]	Baseline clinical data[a] + Curated set[c]
Baseline clinical data [a]	**.**	**0.04**	0.63	**0.003**	**0.03**
Baseline clinical data + Baseline biomarkers[b]	**.**	**.**	1.0	1.0	0.89
Baseline clinical data + Day 2 biomarkers[b]	**.**	**.**	**.**	1.0	0.30
Baseline clinical data + Day 4 biomarkers[b]	**.**	**.**	**.**	**.**	0.83

[a]Baseline clinical data includes infant sex, 5-minute Apgar score, continued resuscitation at 10 minutes, lowest pH, and worst base deficit.
[b]Five most-selected biomarkers at each timepoint after bootstrapped LASSO.
[c]Curated set of biomarkers includes concentrations of C5a, IL-6, and NSE at baseline as well as IL-8 and Tau at day 4.

© 2023 Juul SE et al. *JAMA Network Open.*

eTable 5. Median Biomarker Values by Treatment Group and Rank-Sum Differences Between Groups

Biomarker	Timepoint	Epo	Placebo	Difference (95% CI)	P value
**Anti-Inflammatory**					
IL-1RA	Baseline	6292.1 (14906.5)	6010.3 (14825.5)	371.8 (-1276.0, 2115.1)	0.59
	Day 2	2273.4 (3331.7)	2374.0 (2871.2)	156.7 (-366.7, 691.6)	0.59
	Day 4	2431.0 (3334.3)	2603.6 (3621.6)	54.0 (-461.7, 635.9)	0.85
IL-10	Baseline	75.9 (202.6)	50.7 (97.7)	-9.3 (-30.0, 5.3)	0.22
	Day 2	26.4 (45.2)	31.4 (39.3)	3.6 (-2.0, 9.3)	0.20
	Day 4	14.9 (12.8)	12.3 (11.6)	-1.9 (-4.4, 0.5)	0.13
IL-13	Baseline	1.8 (7.0)	0.8 (4.3)	-0.0 (-0.6, 0.0)	0.42
	Day 2	1.0 (5.5)	1.1 (6.6)	0.0 (-0.2, 0.2)	0.91
	Day 4	1.4 (4.9)	0.8 (5.9)	0.0 (-0.1, 0.1)	0.96
IL-33	Baseline	5.2 (3.7)	6.0 (3.5)	0.5 (-0.3, 1.3)	0.24
	Day 2	4.5 (2.9)	5.2 (3.2)	0.2 (-0.5, 0.9)	0.56
	Day 4	5.2 (2.9)	5.6 (3.0)	0.1 (-0.6, 0.9)	0.70
**Pro-inflammatory**					
IL-8	Baseline	119.3 (219.9)	162.6 (311.7)	8.1 (-25.9, 52.8)	0.68
	Day 2	86.6 (172.3)	106.2 (203.1)	10.7 (-11.2, 35.1)	0.32
	Day 4	74.4 (84.1)	76.6 (93.9)	-0.2 (-16.0, 15.0)	0.97
C5a	Baseline	24650.5 (15979.3)	25089.3 (20858.4)	1580.2 (-2310.1, 5414.3)	0.42
	Day 2	22995.5 (14242.9)	24997.2 (17682.5)	1976.8 (-1336.9, 5360.4)	0.22
	Day 4	19188.7 (12700.5)	18672.7 (10909.0)	-893.5 (-3563.3, 1592.1)	0.49
Fractalkine	Baseline	110.0 (98.3)	115.3 (99.5)	3.1 (-15.3, 22.7)	0.75
	Day 2	98.9 (80.7)	91.5 (92.2)	3.3 (-11.2, 17.8)	0.65
	Day 4	127.0 (75.5)	101.5 (67.2)	-15.5 (-31.9, -1.2)	0.03
ICAM-1	Baseline	478728.8 (352762.3)	538608.8 (312167.1)	12642.7 (-51633.0, 80657.7)	0.74
	Day 2	554166.0 (336392.4)	598993.6 (274063.2)	13116.1 (-52038.9, 77580.7)	0.67
	Day 4	622106.7 (247463.3)	639304.2 (208309.0)	-1413.2 (-59723.8, 55838.6)	0.96
IFN-g	Baseline	4.5 (6.8)	4.5 (6.6)	0.1 (-0.9, 1.1)	0.91
	Day 2	4.1 (5.0)	4.1 (7.1)	0.1 (-0.8, 1.0)	0.85
	Day 4	5.0 (7.7)	4.0 (7.3)	-0.1 (-1.2, 0.9)	0.80
IL-1b	Baseline	0.6 (1.0)	0.5 (0.8)	-0.0 (-0.2, 0.1)	0.90
	Day 2	0.5 (0.6)	0.5 (0.8)	0.0 (-0.1, 0.1)	0.84
	Day 4	0.6 (0.8)	0.5 (0.7)	-0.1 (-0.3, 0.0)	0.10
IL-6	Baseline	44.4 (75.2)	45.3 (123.8)	-3.0 (-15.3, 11.0)	0.66
	Day 2	41.1 (54.8)	40.9 (76.9)	-2.0 (-12.1, 8.2)	0.72
	Day 4	13.4 (19.8)	12.0 (26.0)	-1.3 (-4.8, 2.0)	0.41

© 2023 Juul SE et al. *JAMA Network Open.*

IL-12p70	Baseline	0.6 (0.7)	0.6 (0.6)	0.0 (-0.1, 0.2)	0.51
	Day 2	0.6 (0.9)	0.7 (0.8)	0.0 (-0.1, 0.2)	0.89
	Day 4	0.8 (0.8)	0.7 (0.7)	-0.1 (-0.2, 0.0)	0.15
IL-17A	Baseline	1.4 (1.7)	1.3 (1.6)	-0.0 (-0.3, 0.2)	0.78
	Day 2	1.4 (1.7)	1.3 (1.6)	-0.0 (-0.3, 0.2)	0.70
	Day 4	1.7 (2.8)	1.4 (1.6)	-0.3 (-0.6, -0.1)	0.02
MCP-1	Baseline	270.6 (342.2)	364.1 (486.3)	40.2 (-24.3, 120.1)	0.23
	Day 2	715.0 (645.2)	683.0 (784.6)	10.3 (-131.2, 155.5)	0.87
	Day 4	613.8 (453.9)	591.8 (552.9)	-26.2 (-132.1, 83.5)	0.61
MIP-1a	Baseline	22.2 (21.6)	17.8 (17.1)	-0.9 (-6.2, 3.7)	0.63
	Day 2	26.8 (23.6)	27.0 (18.2)	1.0 (-4.3, 6.2)	0.71
	Day 4	22.6 (20.5)	21.8 (18.3)	-1.1 (-6.1, 3.7)	0.61
MIP-1b	Baseline	52.2 (38.7)	60.8 (39.4)	7.3 (-1.0, 15.7)	0.09
	Day 2	65.5 (43.3)	70.5 (46.6)	2.4 (-7.7, 12.6)	0.62
	Day 4	61.6 (38.0)	56.6 (36.9)	-4.9 (-13.7, 3.8)	0.24
VCAM-1	Baseline	4614152.8 (1597457.9)	4483612.9 (1827712.9)	-163637.7 (-556143.5, 215673.3)	0.37
	Day 2	2503972.0 (1777412.6)	2402529.7 (1557905.0)	-18506.0 (-373570.1, 338334.9)	0.90
	Day 4	4029849.9 (2420664.7)	3760650.1 (2092870.3)	-136420.5 (-575535.0, 327152.4)	0.59
TNF-a	Baseline	27.1 (14.9)	24.2 (18.0)	-2.0 (-5.5, 1.8)	0.32
	Day 2	31.1 (19.3)	32.3 (17.8)	-0.0 (-4.4, 3.9)	1.00
	Day 4	32.6 (16.2)	29.0 (14.1)	-2.1 (-5.8, 1.4)	0.23
**Brain specific**					
GFAP	Baseline	930.2 (1049.5)	920.3 (719.5)	-47.2 (-214.1, 122.5)	0.58
	Day 2	734.2 (821.4)	808.9 (1069.6)	85.3 (-64.2, 237.4)	0.24
	Day 4	1193.9 (1224.1)	1368.5 (1501.0)	130.5 (-148.4, 411.1)	0.35
NSE	Baseline	44699.3 (25181.4)	46894.5 (29760.3)	1672.9 (-3836.7, 7729.9)	0.54
	Day 2	47207.7 (24833.6)	47452.8 (29819.7)	1809.7 (-4453.3, 7943.0)	0.57
	Day 4	33944.1 (22685.2)	34049.6 (18993.5)	-295.0 (-5257.3, 4560.2)	0.90
S100b	Baseline	1659.0 (4453.6)	1540.6 (5437.2)	148.5 (-250.1, 553.4)	0.44
	Day 2	943.2 (1978.2)	1214.6 (1836.2)	125.3 (-95.8, 369.9)	0.25
	Day 4	1055.2 (1286.3)	1078.7 (1112.1)	48.4 (-161.4, 248.4)	0.64
UCHL1	Baseline	2129.9 (1969.1)	2194.0 (2673.3)	8.4 (-345.4, 422.2)	0.95
	Day 2	1901.0 (988.4)	1667.8 (1190.6)	-139.4 (-395.0, 127.9)	0.29
	Day 4	1703.1 (893.2)	1638.2 (1081.4)	-100.4 (-339.0, 149.3)	0.40
Tau	Baseline	177.9 (167.4)	204.1 (193.0)	15.3 (-25.2, 54.6)	0.46
	Day 2	147.3 (191.5)	204.0 (349.4)	24.6 (-11.4, 68.2)	0.18
	Day 4	142.0 (200.9)	249.5 (902.2)	66.5 (15.4, 157.7)	0.01
**Other Growth Factors**					
BDNF	Baseline	224.5 (378.6)	303.9 (461.4)	65.6 (4.7, 137.9)	0.04
	Day 2	139.6 (248.7)	145.0 (217.5)	11.4 (-32.3, 46.0)	0.53

© 2023 Juul SE et al. *JAMA Network Open.*

	Day 4	429.6 (871.4)	275.7 (419.5)	-57.8 (-177.4, 27.8)	0.20
Epo	Baseline	32.7 (169.9)	36.0 (84.5)	-2.6 (-9.4, 3.7)	0.41
	Day 2	1530.2 (4543.6)	34.4 (73.8)	-1466.4 (-1663.1, -1329.2)	<0.001
	Day 4	2681.7 (2447.5)	20.5 (46.6)	-2620.6 (-2866.1, -2496.3)	<0.001
NCAM-1	Baseline	306254.2 (63214.0)	319136.5 (71095.7)	5782.5 (-6698.3, 18799.6)	0.39
	Day 2	297713.4 (76831.1)	301854.5 (73192.3)	3111.8 (-12089.7, 18217.8)	0.68
	Day 4	288952.3 (63329.2)	283207.9 (68907.3)	3130.4 (-11812.1, 17082.7)	0.71
NGB	Baseline	11.6 (10.7)	11.6 (8.4)	0.1 (-1.8, 2.2)	0.84
	Day 2	9.4 (6.3)	10.5 (6.8)	0.7 (-0.9, 2.2)	0.43
	Day 4	10.7 (6.9)	10.0 (5.7)	-1.6 (-3.1, 0.0)	0.05
VEGF	Baseline	27.3 (19.8)	33.4 (23.5)	3.5 (-1.2, 8.4)	0.14
	Day 2	35.8 (21.0)	37.8 (19.0)	2.3 (-2.8, 7.4)	0.35
	Day 4	47.0 (24.8)	41.8 (21.6)	-2.2 (-7.4, 3.4)	0.43

© 2023 Juul SE et al. *JAMA Network Open.*
