# Supplementary material for: Association of High-Dose Erythropoietin With Circulating Biomarkers and Neurodevelopmental Outcomes Among Neonates With Hypoxic Ischemic Encephalopathy: A Secondary Analysis of the HEAL Randomized Clinical Trial
Source: JAMA Netw Open. 2023 Jul 7;6(7):e2322131. doi: 10.1001/jamanetworkopen.2023.22131 (PMC10329214; doi:10.1001/jamanetworkopen.2023.22131)
Supplement: Supplement 3. — Nonauthor Collaborators [file jamanetwopen-e2322131-s003.pdf]

Supplemental Online Content: Nonauthor Collaborators
*First name, last name, and suffix (if applicable) are required and will appear in PubMed.

*Group Name(s): the HEAL consortium

*First Name and Middle Initial(s)	*Last Name	*Suffix (eg, Jr, III)	Academic Degrees	Institution	Location (city, state/province, country)	Role or Contribution, eg, chair, principal investigator	Group (if more than 1 Group listed in the byline) and/or Subgroup (eg, Steering Committee)
Kaashif	Ahmad		MD	Pediatrix Medical Group of San Ar	San Antonio, TX	site PI	HEAL Consortium
Mariana	Baserga		MD	University of Utah	Salt Lake City, UT	site PI	HEAL Consortium
Ellen	Bendel-Stenzel		MD	Mayo Clinic	Rochester, MN	site PI	HEAL Consortium
Kristen	Benninger		MD	Nationwide Children's Hospital	Columbus, OH	site PI	HEAL Consortium
Lina	Chalak		MD	University of Texas Southwestern	Dallas, TX	site PI	HEAL Consortium
Taeun	Chang		MD	Children's National Hospital	Washington, DC	site PI	HEAL Consortium
John	Flibotte		MD	Children's Hospital of Philadelphia	Philadelphia, PA	site PI	HEAL Consortium
Fernando	Gonzalez		MD	University of California, San Franc	San Francisco, CA	site PI	HEAL Consortium
Andrea	Lampland		MD	Children's Minnesota	Minneapolis/St. Paul, MI	site PI	HEAL Consortium
Nathalie	Maitre		MD	Children's Healthcare of Atlanta a	Atlanta, GA	site PI	HEAL Consortium
Amit	Mathur		MD	Saint Louis University School of M	St. Louis, MO	site PI	HEAL Consortium
Stephanie	Merhar		MD	Cincinnati Children's Hospital	Cincinnati, OH	site PI	HEAL Consortium
Ulrike	Mietzsch		MD	University of Washington	Seattle, WA	site PI	HEAL Consortium
Brenda	Poindexter		MD	Children's Healthcare of Atlanta a	Atlanta, GA	site PI	HEAL Consortium
Rakesh	Rao		MD	Washington University in St. Louis	St. Louis, MO	site PI	HEAL Consortium
David	Riley		MD	Cook Children's Medical Center	Ft. Worth, TX	site PI	HEAL Consortium
Christopher	Smyser		MD	Washington University in St. Louis	St. Louis, MO	site PI	HEAL Consortium
Gregory	Sokol		MD	Indiana University School of Medi	Indianapolis, IN	site PI	HEAL Consortium
Krisa	Van Meurs		MD	Stanford University	Stanford, CA	site PI	HEAL Consortium
Joern-Hendrik	Weitkamp		MD	Vanderbilt University Medical Cen	Nashville, TN	site PI	HEAL Consortium
Tai-Wei	Wu		MD	Children's Hospital Los Angeles	Los Angeles, CA	site PI	HEAL Consortium
Toby	Yanowitz		MD	University of Pittsburgh School of Me	Pittsburgh, PA	site PI	HEAL Consortium
