# Supplementary material for: Association of High-Dose Erythropoietin With Circulating Biomarkers and Neurodevelopmental Outcomes Among Neonates With Hypoxic Ischemic Encephalopathy: A Secondary Analysis of the HEAL Randomized Clinical Trial
Source: JAMA Netw Open. 2023 Jul 7;6(7):e2322131. doi: 10.1001/jamanetworkopen.2023.22131 (PMC10329214; doi:10.1001/jamanetworkopen.2023.22131)
Supplement: Supplement 4. — Data Sharing Statement [file jamanetwopen-e2322131-s004.pdf]

Data Sharing Statement

Juul. Association of High-Dose Erythropoietin With Circulating Biomarkers and Neurodevelopmental Outcomes Among Neonates With Hypoxic Ischemic Encephalopathy. *JAMA Netw Open*. Published July 07, 2023. doi:10.1001/jamanetworkopen.2023.22131

Data
Data available: Yes
Data types: Deidentified participant data
How to access data: Data Sharing Statement: We will prepare and share a final research data set that the accepted primary pragmatic trial publication is based upon. The final data set will be structured to maximize future scientific value while protecting patient and health system privacy. The HEAL Data Coordinating Center (DCC) will remove or de-identify all HIPAA-specified direct identifiers. The aim of our data sharing policy is to strive for the least restrictive plan possible while providing appropriate protection for participant privacy, health system privacy, and scientific integrity. Within 9 months of the end of the final year of funding, a final study data set will be accessible via a supervised private data enclave managed by the National Institute of Neurological Disorder and Stroke (NINDS) at: https://www.ninds.nih.gov/Current-Research/Research-Funded-NINDS/Clinical-Research/Archived-Clinical-Research-Datasets. The shared data set will contain all data collected under both the HEAL Trial protocol and HEAL ancillary studies.
When available: beginning date: 07-01-2023

Supporting Documents
Document types: None

Additional Information
Who can access the data: Access will be limited to registered users who submit proposed specific questions or analysis plans and sign a data use agreement according to NINDS guidelines.
Types of analyses: Access will be limited to registered users who submit proposed specific questions or analysis plans and sign a data use agreement according to NINDS guidelines.
Mechanisms of data availability: Within 9 months of the end of the final year of funding, a final study data set will be accessible via a supervised private data enclave managed by the National Institute of Neurological Disorder and Stroke (NINDS) at: https://www.ninds.nih.gov/Current-Research/Research-Funded-NINDS/Clinical-Research/Archived-Clinical-Research-Datasets. The shared data set will contain all data collected under both the HEAL Trial protocol and HEAL ancillary studies.
Any additional restrictions: “Supervised” indicates that individual requests are reviewed to protect the intellectual property rights of the project investigative team by restricting external development of manuscripts using the study data that substantially overlap with those that are already in development by study investigators.
